# Forty Years of Evidence on the Efficacy and Safety of Oral and Injectable Antibiotics for Treating Lyme Disease of Adults and Children: A Network Meta-Analysis

**DOI:** 10.1128/Spectrum.00761-21

**Published:** 2021-11-10

**Authors:** Jiaru Yang, Shiyuan Wen, Jing Kong, Peng Yue, Wenjing Cao, Xin Xu, Yu Zhang, Jingjing Chen, Meixiao Liu, Yuxin Fan, Lisha Luo, Taigui Chen, Lianbao Li, Bingxue Li, Yan Dong, Suyi Luo, Guozhong Zhou, Aihua Liu, Fukai Bao

**Affiliations:** a The Institute for Tropical Medicine, Kunming Medical University, Kunming, China; b Yunnan Province Key Laboratory of Children’s Major Diseases Research, The Affiliated Children’s Hospital, Kunming Medical University, Kunming, China; Peking University People’s Hospital

**Keywords:** *Borrelia burgdorferi*, Lyme arthritis, Lyme disease, Lyme neuroborreliosis, antibiotic treatment, erythema migrans, network meta-analysis

## Abstract

Lyme disease (LD) is a heavy public health burden. The most common manifestations of LD include erythema migrans (EM), Lyme neuroborreliosis (LNB), and Lyme arthritis (LA). The efficacy and safety of antibiotics for treating LD is still controversial. Thus, we performed a network meta-analysis (NMA) to obtain more data and tried to solve this problem. We searched studies in the databases of Embase and PubMed from the date of their establishments until 22 April 2021. Odds ratios (ORs) were used to assess dichotomous outcomes. A total of 31 randomized controlled trials (RCTs) involving 2,748 patients and 11 antibiotics were included. Oral amoxicillin (1.5 g/day), oral azithromycin (0.5 g/day), injectable ceftriaxone, and injectable cefotaxime were effective for treating LD (range of ORs, 1.02 to 1,610.43). Cefuroxime and penicillin were safe for treating LD (range of ORs, 0.027 to 0.98). Amoxicillin was effective for treating EM (range of ORs, 1.18 to 25.66). Based on the results, we thought oral amoxicillin (1.5 g/day), oral azithromycin (0.5 g/day), injectable ceftriaxone, and injectable cefotaxime were effective for treating LD. Cefuroxime and penicillin were safe for treating LD. Amoxicillin was effective for treating EM. We did not observe evidence proving the advantage of doxycycline in efficacy and safety for treating LD, LA, LNB, and EM of children or adults. We did not have sufficient data to prove the significant difference of efficacy for treating LA and LNB in adults and LD in children, the significant difference of safety of oral drugs for treating LD, and the significant difference of safety of drugs for treating EM.

**IMPORTANCE** Some previous studies investigated the efficacy and safety of antibiotics for treating Lyme disease (LD). However, due to technical limitations, several questions regarding the routes of drug administration and the dosages of drug are still unclear, which might be causing problems for clinicians. Hence, we performed network meta-analysis (NMA) to quantitatively analyze the clinical data published during the last 40 years. Here, we demonstrate the evidence regarding the efficacy and safety of antibiotics commonly used for treating LD in adults and children. We found that amoxicillin, azithromycin, ceftriaxone, and cefotaxime were effective for treating LD, but we did not observe significant efficacy and safety of doxycycline for treating LD.

## INTRODUCTION

Lyme disease (LD) was first confirmed in the United States in 1976 in Lyme, Connecticut. It is caused by Borrelia burgdorferi in the United States and Borrelia afzelii and Borrelia garinii in Europe (1, 2) and is transmitted by ticks of the genus *Ixodes* (3). Lyme disease is a heavy public health burden, especially in countries of the Northern hemisphere, including Europe, North America, and most of Asia (4–6). In the United States alone, there are 224,000 to 444,000 diagnosed cases per year (5). The number of reported cases in the United States is still going up over time. A total of 85,000 cases were confirmed in 2006 in Europe. Thus far, this number is estimated to be around 230,000 in western Europe, which is considered to be an underestimation. The incidence in some countries has reached 350 per 100,000 population and is still increasing (3). The highest morbidity occurred in southern Sweden with 464 per 100,000 person-years (4).

The most common manifestations of LD include erythema migrans (EM), Lyme neuroborreliosis (LNB), and Lyme arthritis (LA). EM was detected in 70 to 90% patients with LD, while the percentage of patients presenting with LNB and LA was 10 to 15% and ∼30%, respectively (4, 5, 7, 8). Antibiotics have been used to treat LD for many years. Commonly, the first-line antibiotics for LD treatment are amoxicillin, oral cefuroxime, doxycycline, and intravenous ceftriaxone (7, 9). Cefuroxime, amoxicillin, azithromycin, doxycycline, and oral cephalosporin are considered effective for treating EM (2, 8, 10). Intravenous ceftriaxone, oral doxycycline, oral amoxicillin, oral cefuroxime, penicillin, and cefotaxime are recommended for treating LNB (1–3, 7, 8, 10). Some articles suggested that oral doxycycline, oral amoxicillin, intravenous ceftriaxone, and oral cefuroxime can be used for treating LA (2, 7, 8, 10).

However, some questions regarding the treatment of LD are still unclear, for instance, do different routes of drug administration (oral versus injection) determine their efficacy? Does antibiotics present stable effects on treating various manifestations such as EM, LNB, and LA? Should clinicians use different dosages for treating different manifestations? Should different dosages be used when clinicians use different routes of administration? Is an increased dosage more likely to induce adverse reactions? It is necessary to comprehensively analyze previous studies and data for answering such questions.

However, due to technical limitations, common meta-analysis and review articles are unable to quantitatively compare the efficacy and safety of various antibiotics, which may induce the inaccurate assessments of those antibiotics in previous studies. Hence, we performed a network meta-analysis (NMA) of randomized controlled trials (RCTs) to systematically and quantitively compare the efficacy and safety of oral and injected antibiotics for treating LD, EM, LNB, and LA. Hopefully, our results can supplement previous studies and provide more evidence to help clinicians choose therapeutic schedules.

## RESULTS

### Study characteristics.

**FIG 1:**
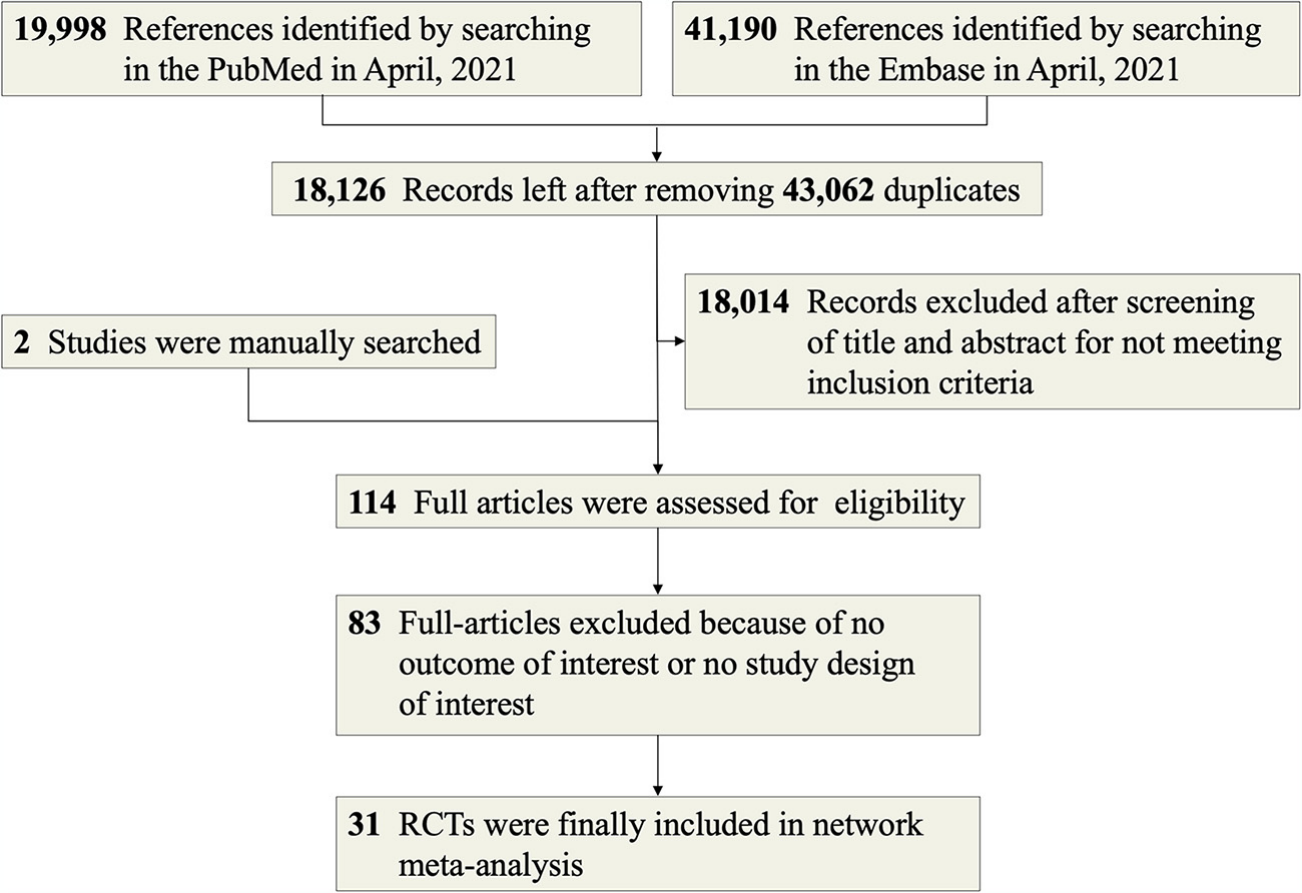
PRISMA flow diagram.

We conducted three searches as explained in Materials and Methods and obtained 19,998 articles from PubMed and 41,190 articles from Embase. After excluding 43,062 duplicates and 18,041 ineligible studies, we selected 29 studies. A further manual search resulted in two more studies; thus, 31 RCTs (11–41) were included in this NMA (Fig. 1). All included studies were published between 1983 and 2018; they involved 2,748 patients and compared 11 antibiotics and 4 treatments (each combined two different drugs). The risk of bias assessment of each included study is shown in Table S1 in the supplemental material. Most studies were at low risk in the assessment, indicating good quality. All characteristics of included studies are shown in Table S2. All detailed results of the pairwise comparisons are shown in Fig. S1 to S18. The results of the significant difference in pairwise comparisons are presented in Table 1.

**TABLE 1:** Pairwise comparisons of efficacy and safety of drugs for treating adults’ LD, LA, and EM^*a*^

Comparison	Network meta-analysis		Test of inconsistency (*P* value)^*g*^
	OR (95% CI)^*b*^	Certainty of evidence^*c*^	
Efficacy of antibiotics for treating Lyme disease^*h*^			
Amoxicillin vs penicillin	4.97 (1.15–21.55)	†††○^*d*^	0.8216
Efficacy of different daily dosages for treating Lyme disease^*i*^			
Amoxicillin (1.5 g) vs azithromycin (0.5 g)	2.14 (1.05–4.36)	†††○^*d*^	0.2514
Amoxicillin (1.5 g) vs cefotaxime (6 g)	18.94 (1.99–180.18)	††○○^*e*^‡	NA
Amoxicillin (1.5 g) vs ceftriaxone (2 g)	14.20 (2.86–70.51)	††○○^*e*^	NA
Amoxicillin (1.5 g) vs doxycycline (0.1 g)	71.85 (5.08–1016.43)	†○○^*e*^	NA
Amoxicillin (1.5 g) vs doxycycline (0.2 g)	7.97 (1.96–32.44)	†††○^*d*^	0.2514
Amoxicillin (1.5 g) vs penicillin (1 megaunit)	17.02 (2.67–108.31)	†††○^*d*^	NA
Amoxicillin (1.5 g) vs penicillin (20 megaunits)	67.20 (6.13–736.85)	††○○^*e*^	NA
Amoxicillin (1.5 g) + probenecid (1.5 g) vs Doxycycline (0.1 g)	20.16 (1.44–282.43)	††○○^*e*^	NA
Amoxicillin (1.5 g) + probenecid (1.5 g) vs penicillin (20 megaunits)	18.86 (1.74–204.53)	††○○^*e*^	NA
Azithromycin (0.25 g) vs doxycycline (0.1 g)	17.79 (1.47–215.73)	††○○^*e*^	NA
Azithromycin (0.25 g) vs penicillin (1 megaunit)	4.21 (1.11–15.95)	†††○^*d*^	0.9802
Azithromycin (0.25 g) vs penicillin (20 megaunit)	16.64 (1.80–153.67)	††○○^*e*^	NA
Azithromycin (0.5 g) vs cefotaxime (6 g)	8.86 (1.02–76.87)	††○○^*e*^	NA
Azithromycin (0.5 g) vs ceftriaxone (2 g)	6.64 (1.53–28.90)	††○○^*e*^	NA
Azithromycin (0.5 g) vs doxycycline (0.1 g)	33.61 (2.57–439.84)	††○○^*e*^	NA
Azithromycin (0.5 g) vs doxycycline (0.2 g)	3.73 (1.07–13.02)	†††○^*d*^	0.2514
Azithromycin (0.5 g) vs penicillin (1 megaunit)	7.96 (1.40–45.24)	††○○^*e*^	NA
Azithromycin (0.5 g) vs penicillin (20 megaunits)	31.44 (3.13–316.13)	††○○^*e*^	NA
Cefotaxime (6 g) vs penicillin (20 megaunits)	3.55 (1.58–7.99)	††○○^*e*^	NA
Ceftriaxone (1 g) vs ceftriaxone (2 g)	18.89 (1.95–182.97)	††○○^*e*^	NA
Ceftriaxone (1 g) vs doxycycline (0.1 g)	95.56 (4.31–2120.64)	††○○^*e*^	NA
Ceftriaxone (1 g) vs doxycycline (0.2 g)	10.60 (1.25–89.66)	††○○^*e*^	NA
Ceftriaxone (1 g) vs penicillin (1 megaunit)	22.63 (1.95–262.89)	††○○^*e*^	NA
Ceftriaxone (1 g) vs penicillin (20 megaunit)	89.38 (4.99–1600.16)	††○○^*e*^	NA
Ceftriaxone (2 g) vs penicillin (3 megaunits)	0.10 (0.01–0.67)	††○○^*e*^	NA
Doxycycline (0.1 g) vs penicillin (3 megaunits)	0.019 (0.001–0.334)	††○○^*e*^	NA
Doxycycline (0.2 g) vs penicillin (20 megaunits)	8.43 (1.21–58.68)	††○○^*e*^	NA
Penicillin (1 megaunit) vs penicillin (3 megaunits)	0.08 (0.01–0.69)	††○○^*e*^	NA
Penicillin (20 megaunit) vs penicillin (3 megaunits)	0.020 (0.001–0.282)	††○○^*e*^	NA
Safety of antibiotics for treating Lyme disease^*j*^			
Amoxicillin vs minocycline	0.16 (0.03–0.77)	†○○○^*f*^	NA
Amoxicillin + probenecid vs cefuroxime	4.77 (1.32–17.17)	†○○○^*f*^	NA
Amoxicillin + probenecid vs penicillin	4.36 (1.22–15.52)	†○○○^*f*^	NA
Azithromycin vs penicillin	1.90 (1.02–3.54)	†○○○^*f*^	0.3088
Cefotaxime vs minocycline	0.10 (0.01–0.74)	††○○^*e*^	NA
Ceftriaxone vs cefuroxime	2.50 (1.25–4.98)	†○○○^*f*^	NA
Ceftriaxone vs doxycycline	1.6306 (1.0003–2.6581)	†††○^*d*^	0.8933
Ceftriaxone vs penicillin	2.29 (1.11–4.72)	††○○^*e*^	NA
Ceftriaxone + doxycycline vs cefuroxime	3.23 (1.45–7.17)	†○^*f*^	NA
Ceftriaxone + doxycycline vs doxycycline	2.10 (1.12–3.96)	††††	NA
Ceftriaxone + doxycycline vs penicillin	2.95 (1.27–6.88)	†○○○^*f*^	NA
Cefuroxime vs minocycline	0.11 (0.02–0.56)	†○○○^*f*^	NA
Doxycycline vs minocycline	0.17 (0.03–0.79)	†○○○^*f*^	NA
Minocycline vs penicillin	8.50 (1.96–36.79)	††○○^*e*^	NA
Safety of different daily dosages for treating Lyme disease^*k*^			
Azithromycin (0.5 g) vs penicillin (1 megaunit)	11.125 (1.033–119.785)	††○○^*e*^	NA
Doxycycline (0.2 g) vs penicillin (1 megaunit)	16.385 (1.208–222.31)	†††○^*d*^	NA
Efficacy of injectable antibiotics for treating Lyme disease^*l*^			
Cefotaxime vs penicillin	3.61 (1.52–8.57)	††○○^*e*^	0.6869
Ceftriaxone vs doxycycline	6.46 (1.09–38.21)	††○○^*e*^	
Ceftriaxone vs penicillin	6.05 (1.73–21.17)	††○○^*e*^	0.6869
Efficacy of oral antibiotics for treating Lyme disease^*m*^			
Amoxicillin vs doxycycline	4.38 (1.06–18.11)	†††○^*d*^	0.4215
Efficacy of different daily oral dosages for treating Lyme disease^*n*^			
Amoxicillin (1.5 g) vs azithromycin (0.5 g)	2.14 (1.05–4.38)	†††○^*d*^	NA
Amoxicillin (1.5 g) vs doxycycline (0.2 g)	7.32 (1.44–37.28)	†††○^*d*^	NA
Amoxicillin (1.5 g) vs penicillin (1 megaunit)	15.64 (2.06–118.55)	†††○^*d*^	NA
Azithromycin (0.25 g) vs penicillin (1 megaunit)	4.21 (1.11–15.95)	†††○^*d*^	0.9802
Azithromycin (0.5 g) vs penicillin (1 megaunit)	7.30 (1.05–50.49)	†††○^*d*^	NA
Penicillin (1 megaunit) vs penicillin (3 megaunit)	0.09 (0.01–0.88)	†††○^*d*^	NA
Safety of oral antibiotics for treating Lyme disease^*o*^			
Amoxicillin + probenecid vs penicillin	4.78 (1.06–21.44)	††○○^*e*^	NA
Efficacy of antibiotics for treating Lyme arthritis^*p*^			
Ceftriaxone vs penicillin	7.37 (1.49–36.44)	††○○^*e*^	NA
Efficacy of antibiotics for treating erythema migrans^*q*^			
Amoxicillin vs ceftriaxone + doxycycline	5.44 (1.19–24.92)	†††○^*d*^	NA
Amoxicillin vs cefuroxime	5.51 (1.18–25.66)	†○○○^*f*^	NA
Amoxicillin vs doxycycline	4.70 (1.42–15.60)	†††○^*d*^	0.4023
Amoxicillin vs penicillin	3.91 (1.14–13.46)	†††○^*d*^	0.9423
Efficacy of different daily dosages for treating erythema migrans^*r*^			
Amoxicillin (1.5 g) vs azithromycin (0.5 g)	2.30 (1.11–4.75)	†††○^*d*^	NA
Amoxicillin (1.5 g) vs doxycycline (0.2 g)	10.91 (2.43–49.03)	†††○^*d*^	NA
Azithromycin (0.25 g) vs penicillin (1 megaunit)	4.21 (1.11–15.95)	†††○^*d*^	NA
Azithromycin (0.5 g) vs doxycycline (0.2 g)	4.74 (1.27–17.70)	†††○^*d*^	NA
Ceftriaxone (1 g) vs doxycycline (0.2 g)	13.48 (1.53–118.67)	††○○^*e*^	NA
Doxycycline (0.2 g) vs penicillin (3 megaunits)	0.13 (0.02–0.84)	††○○^*e*^	NA
Safety of antibiotics for treating erythema migrans^*s*^			
Amoxicillin + probenecid vs penicillin	4.82 (1.17–19.90)	††○○^*e*^	NA
Ceftriaxone vs penicillin	3.17 (1.03–9.72)	†††○^*d*^	0.8323
Ceftriaxone + doxycycline vs cefuroxime	3.15 (1.04–9.52)	†○○○^*f*^	NA
Ceftriaxone + doxycycline vs penicillin	3.65 (1.14–11.71)	†††○^*d*^	NA
Cefuroxime vs minocycline	0.14 (0.02–0.90)	†○○○^*f*^	NA
Minocycline vs penicillin	8.50 (1.73–41.78)	††○○^*e*^	NA

a MD, mean deviation; OR, odds ratio; NA, not available; 95% CI, 95% confident interval.

b If the range of 95% CI includes the threshold value (the threshold values of OR and MD are 1 and 0, respectively), it indicates that the difference of comparison is not significant.

c The certainty of the evidence (according to GRADE) is incorporated in this table and categorized as high (††††), moderate (†††○), low (††○○), or very low (†○○○).

d Downgraded once for study limitations (risk of bias).

e Downgraded twice for study limitations (risk of bias).

f Downgraded three times for study limitations (risk of bias).

g The results of the test for inconsistency are incorporated in this table. *P* < 0.05 indicates the existence of inconsistency.

h Pairwise comparison of efficacy of drugs for treating LD.

i Pairwise comparison of efficacy of different daily dosages for treating LD.

j Pairwise comparison of safety of antibiotics for treating LD.

k Pairwise comparison of safety of different daily dosages for treating LD.

l Pairwise comparison of efficacy of injectable antibiotics for treating LD.

m Pairwise comparison of efficacy of oral antibiotics for treating LD.

n Pairwise comparison of efficacy of different daily oral dosages for treating LD.

o Pairwise comparison of safety of oral antibiotics for treating LD.

p Pairwise comparison of efficacy of antibiotics for treating LA.

q Pairwise comparison of efficacy of antibiotics for treating EM.

r Pairwise comparison of efficacy of different daily dosages for treating EM.

s Pairwise comparison of safety of antibiotics for treating EM.

### Comparing the efficacy and safety of antibiotics for treating adults and children with LD.

**FIG 2:**
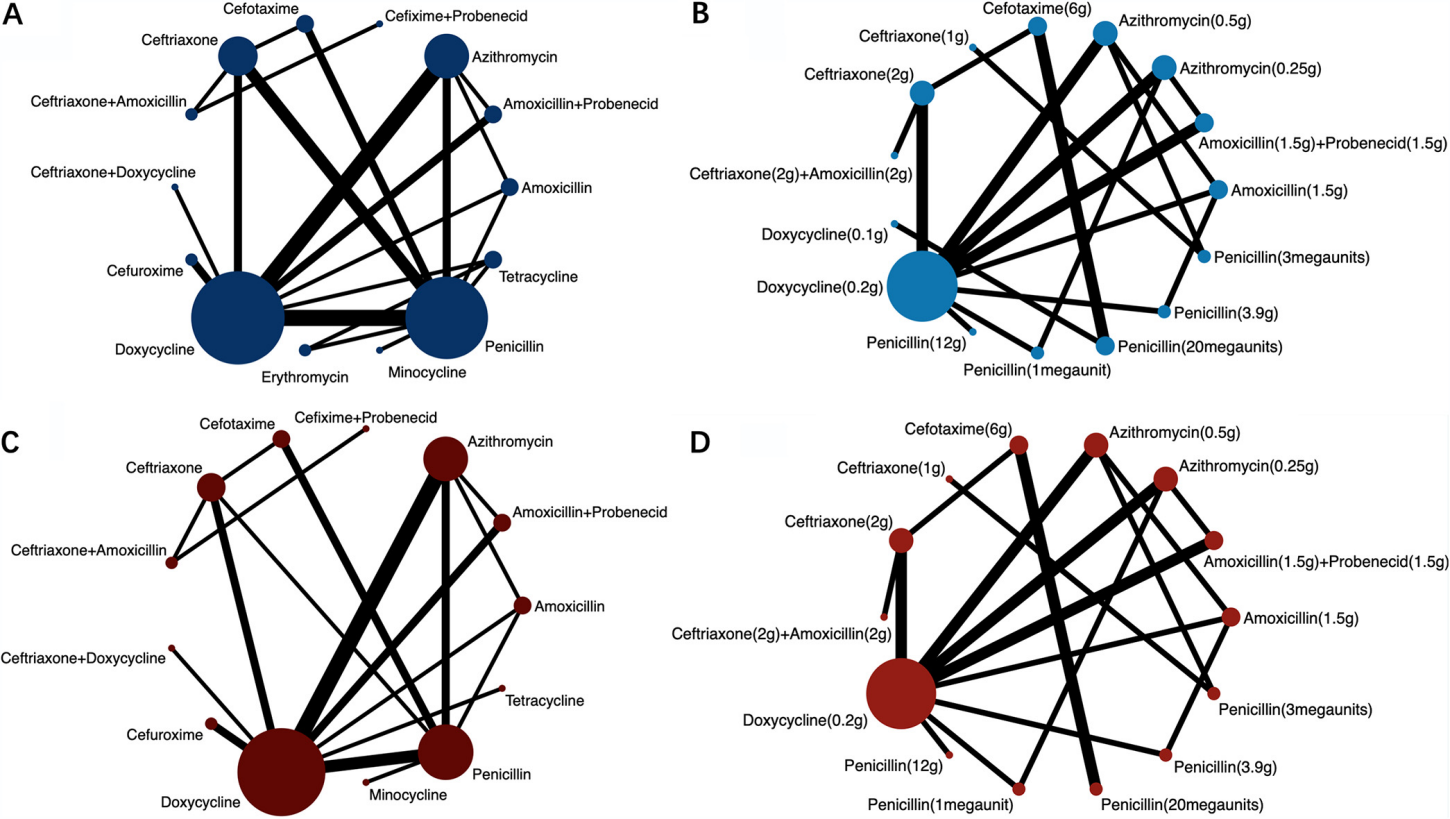
Network meta-analysis graphs of efficacy and safety of different antibiotics and daily dosages for treating LD. Line width is proportional to the number of studies comparing every pair of treatments. The size of every circle is proportional to the number of patients. (A) Network meta-analysis (NMA) graph of comparison of drugs’ efficacy for treating LD. (B) NMA graph of comparison of efficacy of different daily dosages for treating LD. (C) NMA graph of comparison of drug’s safety for treating LD. (D) NMA graph of comparison of safety of different daily dosages for treating LD.

**FIG 3:**
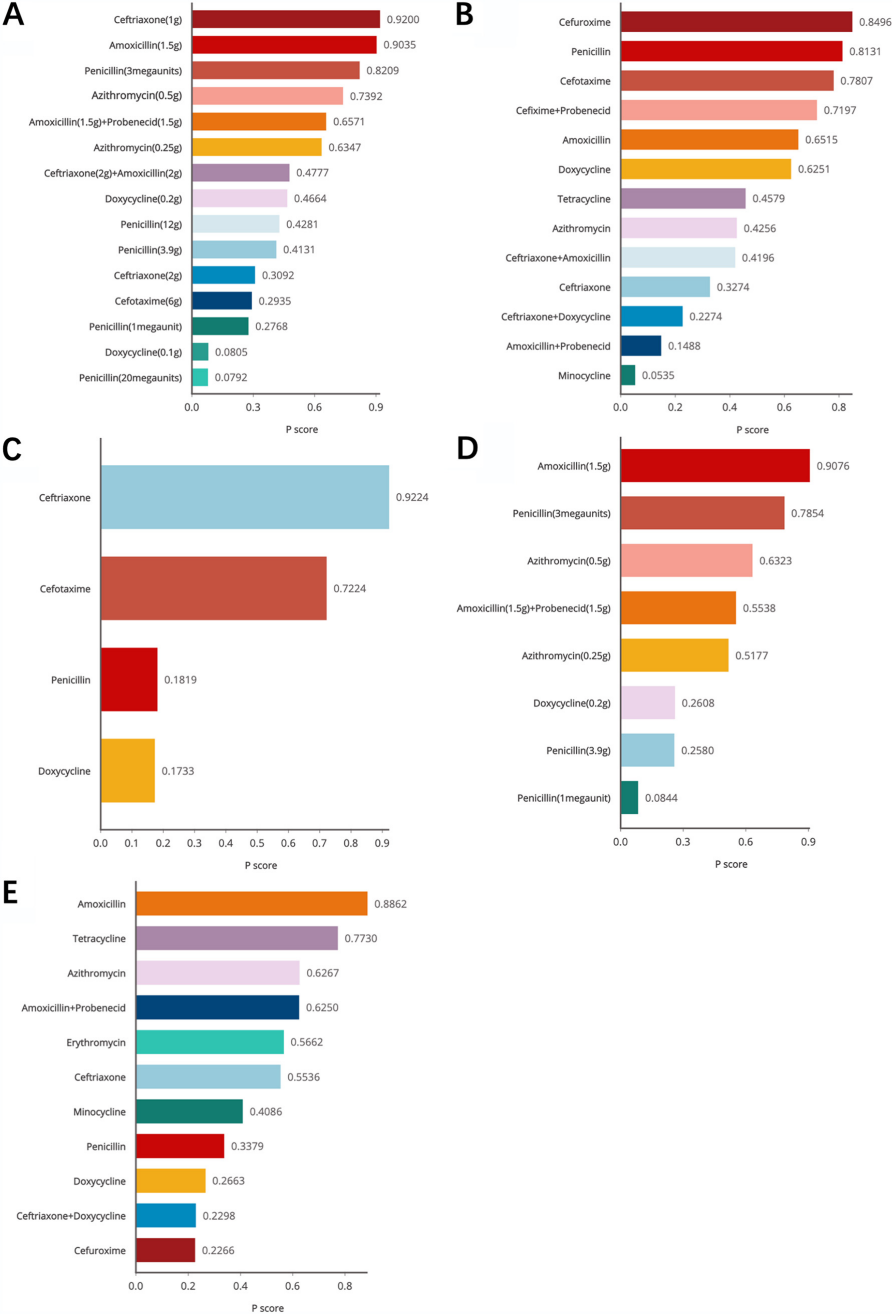
Rankings of efficacy and safety of antibiotics and different daily dosages for treating LD and EM. The P score is an indicator of ranking from a scale of 0 to 1. A higher P score indicates higher ranking of antibiotics. (A) The ranking of efficacy of different dosages for treating LD. (B) The ranking of drugs’ safety for treating LD. (C) The ranking of injectable antibiotic efficacy for treating LD. (D) The ranking of efficacy of different oral dosages for treating LD. (E) The ranking of antibiotic efficacy for treating EM.

By analyzing the efficacy of antibiotics treating adults’ LD (Fig. 2A), we found that amoxicillin was better than penicillin (odds ratio [OR], 4.97; 95% confidence interval [CI], 1.15 to 21.55) (Table 1A). We further analyzed the efficacy of different daily dosages for treating LD (Fig. 2B and Table 1). The efficacy of amoxicillin (1.5 g) was better than that of azithromycin (0.5 g) (OR, 2.14; 95% CI, 1.05 to 4.36), cefotaxime at 6 g (OR, 18.94; 95% CI, 1.99 to 180.18), ceftriaxone at 2 g (OR, 14.20; 95% CI, 2.86 to 70.51), doxycycline at 0.1 g (OR, 71.85; 95% CI, 5.08 to 1,016.43), doxycycline at 0.2 g (OR, 7.97; 95% CI, 1.96 to 32.44), penicillin at 1 megaunit (OR, 17.02; 95% CI, 2.67 to 108.31), and penicillin at 20 megaunits (OR, 67.20; 95% CI, 6.13 to 736.85). Amoxicillin (1.5 g) plus probenecid (1.5 g) was more effective than doxycycline (0.1 g) (OR, 20.16; 95% CI, 1.44 to 282.43) and penicillin (20 megaunits) (OR, 18.86; 95% CI, 1.74 to 204.53). Compared with doxycycline (0.1 g) (OR, 17.79; 95% CI, 1.47 to 215.73), penicillin at 1 megaunit (OR, 4.21; 95% CI, 1.11 to 15.95), penicillin at 20 megaunits (OR, 16.64; 95% CI, 1.80 to 153.67) and azithromycin (0.25 g) were more effective. Moreover, the efficacy of azithromycin (0.5 g) was also better than that of cefotaxime (6 g) (OR, 8.86; 95% CI, 1.02 to 76.87), ceftriaxone (2 g) (OR, 6.64; 95% CI, 1.53 to 28.90), doxycycline at 0.1 g (OR, 33.61; 95% CI, 2.57 to 439.84), doxycycline at 0.2 g (OR, 3.73; 95% CI, 1.07 to 13.02), penicillin at 1egaunit (OR, 7.96; 95% CI, 1.40 to 45.24), and penicillin at 20 megaunits (OR, 31.44; 95% CI, 3.13 to 316.13). Cefotaxime (6 g) was better than penicillin (20 megaunits) (OR, 3.55; 95% CI, 1.58 to 7.99). Ceftriaxone (1 g) was more effective than ceftriaxone (2 g) (OR, 18.89; 95% CI, 1.95 to 182.97), doxycycline at 0.1 g (OR, 95.56; 95% CI, 4.31 to 2,120.64), doxycycline at 0.2 g (OR, 10.60; 95% CI, 1.25 to 89.66), penicillin at 1 megaunit (OR, 22.63; 95% CI, 1.95 to 262.89), and penicillin at 20 megaunits (OR, 89.38; 95% CI, 4.99 to 1,600.16). Compared with penicillin (3 megaunits), ceftriaxone (2 g) (OR, 0.10; 95% CI, 0.01 to 0.67), doxycycline (0.1 g) (OR, 0.019; 95% CI, 0.001 to 0.334), penicillin at 1 megaunit (OR, 0.08, 95% CI, 0.01 to 0.69) and penicillin at 20 megaunits (OR, 0.020; 95% CI, 0.001 to 0.282) showed less efficacy. Doxycycline (0.2 g) was better than penicillin (20 megaunits) (OR, 8.43; 95% CI, 1.21 to 58.68). According to the ranking of efficacy, ceftriaxone (1 g) (P score = 0.92), amoxicillin (1.5 g) (P score = 0.9035), penicillin (3 megaunits) (P score = 0.8209), and azithromycin (0.5 g) (P score = 0.7392) were effective for treating LD (Fig. 3A).

As for the safety of drugs (Fig. 2C), amoxicillin (OR, 0.16; 95% CI, 0.03 to 0.77), cefotaxime (OR, 0.10; 95% CI, 0.01 to 0.74), cefuroxime (OR, 0.11; 95% CI, 0.02 to 0.56) and doxycycline (OR, 0.17; 95% CI, 0.03 to 0.79) were safer than minocycline (Table 1). Cefuroxime (Table 1) was safer than amoxicillin plus probenecid (OR, 4.77; 95% CI, 1.32 to 17.17), ceftriaxone (OR, 2.50; 95% CI, 1.25 to 4.98), ceftriaxone plus doxycycline (OR, 3.23; 95% CI, 1.45 to 7.17). Ceftriaxone (OR, 1.63; 95% CI, 1.0003 to 2.66), and ceftriaxone plus doxycycline (OR, 2.10; 95% CI, 1.12 to 3.96) were more likely to cause adverse reactions than doxycycline. Moreover, amoxicillin plus probenecid (OR, 4.36; 95% CI, 1.22 to 15.52), azithromycin (OR, 1.9; 95% CI, 1.02 to 3.54), ceftriaxone (OR, 2.29; 95% CI, 1.11 to 4.72), ceftriaxone plus doxycycline (OR, 2.95; 95% CI, 1.27 to 6.88), and minocycline (OR, 8.50; 95% CI, 1.96 to 36.79) were more likely to cause side effect than penicillin. In general, cefuroxime (P score = 0.8496), penicillin (P score = 0.8131), and cefotaxime (P score = 0.7807) were less likely to cause adverse reactions (Fig. 3B). The significant differences in safety of daily dosages (Fig. 2D) were only observed in comparison of azithromycin (0.5 g) (OR, 11.125; 95% CI, 1.033 to 119.785) and doxycycline (0.2 g) (OR, 16.385; 95% CI, 1.208 to 2,223.1) versus penicillin (1 megaunit) (Table 1).

Next, we analyzed the efficacy of antibiotics for treating children’s LD (Fig. S19A); we did not observe significant differences of efficacy among these drugs (Fig. S5).

### Comparing the efficacy and safety of different routes of administration for treating adults’ LD.

For injectable drugs (Fig. S19B), cefotaxime (OR, 3.61; 95% CI, 1.52 to 8.57) and ceftriaxone (OR, 6.05; 95% CI, 1.73 to 21.17) were more effective than penicillin. Ceftriaxone (OR, 6.46; 95% CI, 1.09 to 38.21) was more effective than doxycycline (Table 1). Thus, ceftriaxone (P score = 0.9224) and cefotaxime (P score = 0.7224) were at the first and second positions in the ranking (Fig. 3C).

As for the efficacy of oral drugs (Fig. S20A), the only significant difference was observed in the comparison of amoxicillin versus doxycycline (OR, 4.38; 95% CI, 1.06 to 18.11) (Table 1). Subsequently, we assessed the efficacy of different daily dosages (Fig. S20B). We found that amoxicillin (1.5 g) was more effective than azithromycin (0.5 g) (OR, 2.14; 95% CI, 1.05 to 4.38), doxycycline (0.2 g) (OR, 7.32; 95% CI, 1.44 to 37.28), and penicillin (1 megaunit) (OR, 15.64; 95% CI, 2.06 to 118.55). Also, azithromycin at 0.25 g (OR, 4.21; 95% CI, 1.11 to 15.95) and azithromycin at 0.5 g (OR, 7.30; 95% CI, 1.05 to 50.49) were more effective than penicillin (1 megaunit). Penicillin at 1 megaunit was less effective than penicillin at 3 megaunits (OR, 0.09; 95% CI, 0.01 to 0.88) (Table 1). Based on the ranking (Fig. 3D), amoxicillin (1.5 g) (P score = 0.9076) was the most effective oral drug for treating LD.

Next, we assessed the safety of oral drugs (Fig. S20C) and different oral dosages (Fig. S20D). For oral drugs; amoxicillin plus probenecid was more likely to induce adverse reactions than penicillin (OR, 4.78; 95% CI, 1.49 to 36.44) (Table 1). Conversely, there was no significant difference of safety in oral daily dosages (Fig. S10).

### Comparing the efficacy and safety of different drugs and daily dosages for treating adults’ LA, LNB, and EM.

By assessing the efficacy of drugs for treating LA (Fig. S19C), we found that ceftriaxone was better than penicillin (OR, 7.37; 95% CI, 1.49 to 36.44) (Table 1).

Next, we compared the efficacy of drugs (Fig. S19D) and different dosages (Fig. S19E) for treating LNB, but we did not observe significant differences among them (Fig. S12 and S13).

As for the efficacy of drugs for treating EM (Fig. S21A), amoxicillin was more effective than ceftriaxone plus doxycycline (OR, 5.44; 95% CI, 1.19 to 24.92), cefuroxime (OR, 5.51; 95% CI, 1.18 to 25.66), doxycycline (OR, 4.70; 95% CI, 1.42 to 15.60), and penicillin (OR, 3.91; 95% CI, 1.14 to 13.46) (Table 1). The ranking indicated that amoxicillin was the best drug for treating EM (Fig. 3E). We further evaluated the efficacy of different dosages for treating EM (Fig. S21B and Table 1); amoxicillin (1.5 g) was more effective than azithromycin (0.5 g) (OR, 2.30; 95% CI, 1.11 to 4.75) and doxycycline (0.2 g) (OR, 10.91; 95% CI, 2.43 to 49.03). Azithromycin (0.25 g) (OR, 4.21; 95% CI, 1.11 to 15.95) was better than penicillin (1 megaunit). Azithromycin (0.5 g) (OR, 4.74; 95% CI, 1.27 to 17.70) and ceftriaxone (1 g) (OR, 13.48; 95% CI, 1.53 to 118.67) were better than doxycycline (0.2 g). Doxycycline (0.2 g) (OR, 0.13; 95% CI, 0.02 to 0.84) was less effective than penicillin (3 megaunits). We also compared the therapy duration of drugs for treating EM (Fig. S21C), but we found that there was no significant difference among those drugs (Fig. S16).

Safety of antibiotics treating EM was also evaluated (Fig. S21D). Amoxicillin plus pebenecid (OR, 4.82; 95% CI, 1.17 to 19.90), ceftriaxone (OR, 3.17; 95% CI, 1.03 to 9.72), ceftriaxone plus doxycycline (OR, 3.65; 95% CI, 1.14 to 11.71), and minocycline (OR, 8.50; 95% CI, 1.73 to 41.78) were more likely to cause adverse reactions than penicillin (Table 1). Ceftriaxone plus doxycycline (OR, 3.15; 95% CI, 1.04 to 9.52) showed more risk than cefuroxime, while cefuroxime was safer than minocycline (OR, 0.14; 95% CI, 0.02 to 0.90) (Table 1).

### The certainty of evidence and testing of inconsistency.

All results of test of inconsistency are shown in Table 1 and Fig. S1 to S18. No significant difference (*P* < 0.05) was observed, as shown in Table 1 and Fig. S1 to S18.

## DISCUSSION

The choice of treatment for LD has attracted considerable research interest for many years. Nevertheless, owing to the limitations of analysis techniques, common review articles and meta-analyses have been unable to conduct quantitively indirect comparisons to comprehensively assess the efficacy and safety of various antibiotics. A previous study used NMA to assess the efficacy and safety of antibiotics for treating EM. The results suggested that neither the antibiotic agent nor the treatment modality contributed to comparative effectiveness or drug-related adverse outcomes (4). However, that analysis included only 17 studies for comparing 9 treatments, indicating a relatively small sample size. Furthermore, it did not discuss the efficacy and safety of different routes of administration. Another study discussed the effects of antibiotics for treatment of LNB, but that study only discussed seven RCTs (42). Consequently, we searched for more articles to perform a more comprehensive and systematic NMA to supplement previous studies.

Generally, amoxicillin, ceftriaxone, cefotaxime, cefuroxime, penicillin, and doxycycline are regarded as the most effective antibiotics for treating LD. (4, 7, 43) Based on our data, we thought amoxicillin was an effective drug for treating adults’ LD and EM. Moreover, 1.5 g oral amoxicillin per day was an effective dosage for treating adults’ LD. This result was the same as the clinical practice guideline of the Infectious Diseases Society of America (IDSA) (43). Moreover, we thought that injectable ceftriaxone and cefotaxime were also effective for treating LD. These two drugs were also recommended by the IDSA guideline and other previous studies for treating LD (7, 8, 43). According to the IDSA guideline, 2 g injectable ceftriaxone per day was an effective dosage for treating LD, but we found that 1 gram ceftriaxone per day was probably also an effective dosage. Penicillin and cefuroxime showed good safety for treating LD, but their efficacy was moderate. Doxycycline was recommended by the IDSA guideline for treating LD, EM, LNB, and LA (43). However, we observed that its efficacy and safety were moderate. In fact, we did not observe evidence proving the advantage of doxycycline in efficacy and safety for treating LD, LA, LNB, and EM of children and adults. Considering the various limitations in our study, we thought its efficacy and safety might be underestimated, implying that we need more research on its efficacy and safety.

Furthermore, we noticed that the efficacy of oral azithromycin (0.5 g/day) for treating LD was a little bit better than that of other drugs. Hence, we assumed it could be an alternative antibiotic for treating LD. The IDSA guideline also recommended oral azithromycin (0.5 g/day) as an alternative drug for treating LD (43).

As for other pairwise comparisons, we did not have sufficient data to prove a significant difference of efficacy among drugs for treating adults’ LA and LNB and children’s LD, the significant difference of safety of oral drugs for treating LD, and the significant difference of safety of drugs for treating EM.

Compared with previous reviews, we included more articles to assess the efficacy and safety of antibiotics for treating LD. Further, we obtained more data regarding antibiotics treating LD to supplement previous studies. However, we still highlight that more research is needed in the future to validate these findings and further discuss potential issues.

There were some limitations in our study. First, the number of included studies was still relatively small, which may have caused the NMA results to deviate from the true results. Second, the number of patients treated with some of the mentioned drugs was relatively small, resulting in the analysis results having a wide range of 95% CI. Most of the nonsignificant differences in pairwise comparisons were caused by a wide range of 95% CI. Third, some included studies were open-label studies; such study results might be exaggerated or underestimated. Fourth, we included older studies in which the diagnosis might be less valid. Last, the included studies used diverse therapy durations, which may influence the results.

Overall, oral amoxicillin (1.5 g/day), azithromycin (0.5 g/day), and injectable ceftriaxone and cefotaxime were effective for treating LD. Cefuroxime and penicillin were safe for treating LD. Amoxicillin was effective for treating EM. We did not observe evidence proving the advantage of doxycycline in efficacy and safety for treating LD, LA, LNB, and EM of children or adults. We did not have sufficient data to prove a significant difference of efficacy among drugs for treating adults’ LA and LNB and children’s LD, a significant difference of safety of oral drugs for treating LD, or a significant difference of safety of drugs for treating EM.

## MATERIALS AND METHODS

This NMA was conducted according to the criteria of the Preferred Reporting Items for Systematic Reviews and Meta-Analyses (PRISMA) (44). The Grading of Recommendations Assessment, Development, and Evaluation (GRADE) system was used to assess the certainty of evidence derived from the results of the NMA. GRADE is an evaluation system that can assess the quality of an NMA results across four levels—high, moderate, low, and very low (45).

### Search strategies and inclusion criteria.

We performed three searches in the databases of Embase and PubMed from the date of their establishments until 22 April 2021. For the first search, we used the terms “Lyme,” “*Borrelia*,” and “*burgdorferi*” combined with a list of antibiotics. We hoped to analyze as many antibiotics as possible; therefore, we expanded the list to 30 antibiotics, namely, doxycycline, amoxicillin, cefuroxime, azithromycin, erythromycin, clarithromycin, ceftriaxone, cefotaxime, chloramphenicol, penicillin, kanamycin, norfloxacin, ofloxacin, pefloxacin, cephaloridine, enoxacin, cefoxitin, fleroxacin, spectinomycin, cefixime, cefpodoxime, sitafloxacin, ciprofloxacin, tetracycline, rifampicin, telithromycin, levofloxacin, minocycline, aureomycin, and cephalosporin. In the second search, we used “Lyme,” “*Borrelia*,” “*burgdorferi*,” “therapy,” “treatment,” “cure,” “drug,” “antibiotic,” and “antimicrobial” as search terms. In the final search, the search terms were “Lyme,” “*Borrelia*,” “*burgdorferi*,” “randomized controlled trial,” “controlled clinical trial,” “random allocation,” “double-blind,” “single-blind,” “survival,” “treatment,” “therapy,” “comparison,” “comparative,” “effective,” and “efficacy.”

We included RCTs that compared the efficacy or safety of antibiotics treating LD, LNB, or LA. All patients in these RCTs were required to have a clinically confirmed diagnosis of LD. The diagnosis and confirmation should have been based on patients’ clinical symptoms and the results of laboratory tests such as enzyme-linked immunosorbent assay (ELISA), Western blot, or PCR.

All included studies were evaluated independently by two reviewers (J.Y. and S.W.). Any disagreement was addressed by discussion with a third reviewer (F.B. or A.L.) until a consensus was reached.

### Data extraction and outcomes.

The first outcome was efficacy, which was defined as patients completely recovered from LD after one course of treatment with no recurrence. For assessing this outcome, we extracted two categories of data that included the number of patients treated with a certain antibiotic and the number of patients whose manifestation completely disappeared and did not recur after treatment with this drug.

The second outcome was the antibiotic therapy duration for EM, since this was an essential indicator to assess the efficacy of antibiotics. The therapy duration was defined as the period from the onset of therapy to the disappearance of EM; the measuring unit was days. We recorded the number of patients treated with a certain drug as well as the mean and standard deviation (SD) of the therapy duration. Some studies reported median and range of therapy duration rather than mean and SD; hence, we used the method proposed by McGrath et al. (46) to calculate the mean and SD.

The third outcome was safety, defined as the rate of patients who had adverse reactions after or during treating with an antibiotic. A lower rate indicates higher safety for a drug. We recorded the number of patients receiving antibiotics and the number of patients who complained of adverse reactions during or after antibiotic use to assess drug safety.

### Statistical analysis.

We used a tool invented by the Cochrane Collaboration to assess the risk of bias of all included studies (47). Next, we utilized NMA to analyze all extracted data, as it provides a generalization of pairwise meta-analysis that compares all pairs of interventions within several treatments for the same condition (48, 49), enabling us to systematically assess the efficacy, safety, and therapy duration of antibiotics for treating LD (50, 51).

A frequentist approach was used for conducting this NMA. Such an approach has been utilized in several NMAs and its efficacy proven (4, 52–54). ORs and 95% CIs were used to assess dichotomous outcomes (i.e., efficacy and safety), while mean deviation (MD) and 95% CI were used to assess continuous outcomes (i.e., therapy duration). The study effect sizes were synthesized by a random-effects model.

Inconsistency, being an indispensable indicator for evaluating the quality of an NMA, refers to the difference of estimate of effect between direct evidence and indirect evidence. Therefore, we utilized the back-calculation method to quantitively evaluate the inconsistency of this NMA. Such a method is based on the Z test and provides the *P* to help determine the inconsistency. Namely, *P* < 0.05 indicates the existence of inconsistency in an NMA (55). Next, we ranked the efficacy, safety, and therapy duration of antibiotics by calculating their P score, which is used to measure the extent of certainty that a treatment is better than others, averaged over all competing treatments (56). The scale of the P score is from 0 (worst) to 1 (best). Namely, if a drug has a high P score, it means the drug has better efficacy or safety.

This NMA was conducted using the Netmeta package of R version 3.6.0 (R Foundation) and Stata version 14.0 (StataCorp). This study was registered with PROSPERO, no. CRD42020177184.

### Data availability.

All data generated or analyzed during this study are included in this published article and the supplemental material.
