# Supplementary material for: Forty Years of Evidence on the Efficacy and Safety of Oral and Injectable Antibiotics for Treating Lyme Disease of Adults and Children: A Network Meta-Analysis
Source: Microbiol Spectr. 2021 Nov 10;9(3):e00761-21. doi: 10.1128/Spectrum.00761-21 (PMC8579938; doi:10.1128/Spectrum.00761-21)
Supplement: SUPPLEMENTAL FILE 1 — Supplemental material. Download SPECTRUM00761-21_Supp_1_seq8.pdf, PDF file, 3.2 MB [file spectrum00761-21_supp_1_seq8.pdf]

Supplemental Material for Forty years of evidence on the efficacy and safety of oral and injectable antibiotics for treating Lyme Disease of adults and children: A network meta-analysis

Table S1 Assessment of risk of bias
Table S2 Characteristics of all included studies
FIG S1 Pairwise comparison and inconsistency test of efficacy for antibiotics treating LD
FIG S2 Pairwise comparison and inconsistency test of efficacy for different daily dosages of antibiotics treating LD
FIG S3 Pairwise comparison and inconsistency test of safety for antibiotics treating LD
FIG S4 Pairwise comparison and inconsistency test of safety for different daily dosages of antibiotics treating LD
FIG S5 Pairwise comparison and inconsistency test of efficacy for antibiotics treating children's LD
FIG S6 Pairwise comparison and inconsistency test of efficacy for injectable antibiotics treating LD
FIG S7 Pairwise comparison and inconsistency test of efficacy for oral antibiotics treating LD
FIG S8 Pairwise comparison and inconsistency test of efficacy for different daily dosages of oral antibiotics treating LD
FIG S9 Pairwise comparison and inconsistency test of safety for oral antibiotics treating LD
FIG S10 Pairwise comparison and inconsistency test of safety for different daily dosages of oral antibiotics treating LD
FIG S11 Pairwise comparison and inconsistency test of efficacy for antibiotics treating LA
FIG S12 Pairwise comparison and inconsistency test of efficacy for antibiotics treating LNB
FIG S13 Pairwise comparison and inconsistency test of efficacy for different daily dosages of antibiotics treating LNB
FIG S14 Pairwise comparison and inconsistency test of efficacy for antibiotics treating EM
FIG S15 Pairwise comparison and inconsistency test of efficacy for different daily dosages of antibiotics treating EM
FIG S16 Pairwise comparison and inconsistency test of therapy duration for antibiotics treating EM
FIG S17 Pairwise comparison and inconsistency test of safety for antibiotics treating EM
FIG S18. Pairwise comparison and inconsistency test of safety for different daily dosages of antibiotics treating EM
FIG S19 Network Meta-analysis Graphs of Treatments.
FIG S20 Network Meta-analysis Graphs of Oral Drugs.
FIG S21 Network Meta-analysis Graphs of Drugs Treating EM.
Reference S

	Random sequence generation (selection bias)	Allocation concealment (selection bias)	Blinding of participants and personnel (performance bias)	Blinding of outcome assessment (detection bias)	Incomplete outcome data (attrition bias)	Selective reporting (reporting bias)	Other bias
Maja Arnez, 1999(1)	Low risk	Unclear	Unclear	Unclear	Low risk	Low risk	Low risk
Benjamin J. Luft, 1988(2)	Low risk	Unclear	Unclear	Unclear	Low risk	Low risk	Unclear
Unn Ljøstad, 2008(3)	Low risk	Unclear	Low risk	Low risk	Low risk	Low risk	Low risk
R. R. Mtillegger, 1991(4)	Low risk	Unclear	Unclear	Unclear	Low risk	Low risk	Low risk
Hans-Walter Pfister, 1991(5)	Low risk	Unclear	High risk	High risk	Low risk	Low risk	High risk
Tea Nizicˇ, 2012(6)	Low risk	Unclear	Unclear	Unclear	Low risk	Low risk	Low risk
Elena M. Massarotti, 1992(7)	Low risk	Unclear	Unclear	Unclear	Low risk	Low risk	Low risk
Raymond J. Dattwyler, 1988(8)	Low risk	Unclear	Unclear	Unclear	Low risk	Low risk	Low risk
W. Kohlhepp, 1989(9)	Low risk	Unclear	Unclear	Unclear	Low risk	Low risk	Low risk
Allen C. Steere, 1983(10)	Low risk	Unclear	Unclear	Unclear	Low risk	Low risk	Low risk
K. Weber, 1990(11)	Low risk	Unclear	Unclear	Unclear	Low risk	Low risk	Unclear
Raymond J. Dattwyler, 1990(12)	Low risk	Unclear	Unclear	Unclear	Low risk	Low risk	Unclear
Benjamin J. Luft, 1996(13)	Low risk	Low risk	Low risk	Low risk	Low risk	Low risk	Low risk
Maja Arnež, 2015(14)	Low risk	Unclear	Unclear	Unclear	Low risk	Low risk	Unclear
F. Strle, 1993(15)	Low risk	Unclear	Unclear	Unclear	Low risk	Low risk	High risk
D. Hassler, 1990(16)	Low risk	Unclear	Unclear	Unclear	Low risk	Low risk	Unclear
Hans-Walter Pfister, 1989(17)	Low risk	Unclear	Unclear	Unclear	Low risk	Low risk	Unclear

Raymond J. Dattwyler, 1997(18)	Low risk	Unclear	High risk	Unclear	Low risk	Low risk	Unclear
Stephen C. Eppes, 2002(19)	Low risk	Unclear	High risk	Unclear	Low risk	Low risk	Unclear
B. Barsic, 2000(20)	low risk	Unclear	High risk	Unclear	Low risk	Low risk	Unclear
Robert B. Nadelman, 1992(21)	low risk	Unclear	High risk	low risk	Low risk	Low risk	Unclear
Steven W. Luger, 1995(22)	low risk	Unclear	High risk	low risk	Low risk	Low risk	Unclear
M. Karlsson, 1994(23)	low risk	Unclear	Unclear	Unclear	Low risk	Low risk	Unclear
J. Oksi, 1998(24)	low risk	Unclear	Unclear	Unclear	Low risk	Low risk	Unclear
Knut Eirik Eliassen, 2018(25)	low risk	High risk	High risk	Low risk	Low risk	Low risk	Unclear
John Nowakowski, 1995(26)	Low risk	Unclear	High risk	Unclear	Low risk	Low risk	High risk
Gary P. Wormser, 2003(27)	Low risk	Low risk	Low risk	Low risk	Low risk	Low risk	Unclear
J. Oksi, 2007(28)	Low risk	Low risk	Low risk	Low risk	Low risk	Low risk	Low risk
Franc Strle, 1992(29)	Low risk	Unclear	Unclear	Unclear	Low risk	Low risk	Unclear
E Breier, 1996(30)	Low risk	High risk	High risk	Unclear	Low risk	Low risk	Unclear
K. Weber, 1993(31)	Low risk	Unclear	Unclear	Unclear	Low risk	Low risk	Unclear

Table S1 Assessment of risk of bias

Study ID	Methods of diagnose	Therapy duration	Age	Intervene 1				Intervene 2				Intervene 3			
				Drug	Dosage	Route of administration	Adverse reaction	Drug	Dosage	Route of administration	Adverse reaction	Drug	Dosage	Route of administration	Adverse reaction
Maja Arnez, 1999(1)	History of ECM, clinical signs and symptoms, antibody	14 d	Children	Cefuroxime	30mg/K g/d	Oral	Yes	Penicillin	100000 IU/Kg/d	Oral	Yes	N/A	N/A	N/A	N/A
Benjamin J. Luft, 1988(2)	History of ECM,clinical evidence,immunological evidence	10d &14 d	Unspecified	Penicillin	18 x 10^6 units/d	Injection	Unspecified	Ceftriaxone	4 g/d	Injection	Unspecified	N/A	N/A	N/A	N/A
Unn Ljøstad, 2008(3)	Clinical signs and symptoms, CSF white-cell count ＞ 5 per mL / intrathecal production of specific Borrelia burgdorferi antibodies / acrodermatitis chronicum atrophicans	14 d	Adult	Doxycycline	200mg/d	Oral	Yes	Ceftriaxone	2g/d	Injection	Yes	N/A	N/A	N/A	N/A
R. R. Mtillegger, 1991(4)	Clinical signs and symptoms, CSF laboratory tests (ELISA or direct cultivation of B.b)	14 d	Children	Penicillin	400000 - 500000 I.U./kg/d	Injection	Yes	Ceftriaxone	75-93 mg/kg/d	Injection	Yes	N/A	N/A	N/A	N/A
Hans-Walter Pfister, 1991(5)	Clinical signs and symptoms, history, serum or CSF antibodies, or isolation from CSF	10 d	Adult	Ceftriaxone	2g/d	Injection	Yes	Cefotaxime	6g/d	Injection	Yes	N/A	N/A	N/A	N/A
Tea Nizicˇ, 2012(6)	Clinical signs and symptoms, history, antibodies, isolation, laboratory tests	14 d	Children	Clarithromyci n	15 mg/kg/d	Unspecified	Yes	Amoxicillin	50 mg/kg/d	Unspecified	Yes	N/A	N/A	N/A	N/A
Elena M. Massarotti, 1992(7)	Clinical symptoms (EM, Flu-like), antibodies	5-10 d	Adult	Azithromycin	1g/d	Oral	Yes	Amoxicillin	500 mg tid	Oral	Unspecified	Doxycycline	100 mg bid	Oral	Yes

Raymond J. Dattwyler, 1988(8)	Clinical signs and symptoms (EM,objective evidence of involvement of two or more organ systems), evidence of specific immunological reactivity to B. burgdorferi	10-14 d	Adult	Ceftriaxone	4g/d	Injection	Unspecified	Penicillin	24 million U /d	Injection	Unspecified	N/A	N/A	N/A	N/A
W. Kohlhepp, 1989(9)	Clinical findings, Laboratory findings, Elevated IgM, IgA, IgG index in the CSF	10 d	Unspecified	Penicillin	20 mega units/d	Injection	Unspecified	Doxycycline	100mg/d	Injection	Unspecified	N/A	N/A	N/A	N/A
Allen C. Steere, 1983(10)	Clinical signs and symptoms (skin lesion)	10 d	Adult	Penicillin	1g/d	Oral	Unspecified	Erythromycin	1g/d	Oral	Unspecified	Tetracycline	1g/d	Oral	Unspecified
K. Weber, 1990(11)	Clinical manifestation and serology immunofluorescence test	12 d	Adult	Penicillin	3 million U/d	Oral	Yes	Ceftriaxone	1g/d	Injection	Yes	N/A	N/A	N/A	N/A
Raymond J. Dattwyler, 1990(12)	Clinical manifestation and serology immunofluorescence test	21 d	Adult	Amoxicillin	1.5g/d	Unspecified	Yes	Doxycycline	200mg/d	Unspecified	Yes	N/A	N/A	N/A	N/A
Benjamin J. Luft, 1996(13)	Clinical evaluations and Serology (ELISA)	7 or 20 d	Adult	Amoxicillin	1.5g/d	Oral	Yes	Azithromycin	500mg/d	Oral	Yes	N/A	N/A	N/A	N/A
Maja Arnež, 2015(14)	Unspecified	Unspecified	Children	Azithromycin	10mg/kg /d	Unspecified	Yes	Amoxicillin	50mg/kg/d	Unspecified	Yes	N/A	N/A	N/A	N/A
F. Strle, 1993(15)	Clinical manifestation IgM and IgG antibody titers against B. burgdorferi	14 d	Adult	Azithromycin	500mg/d	Unspecified	Yes	Doxycycline	200mg/d	Unspecified	Yes	N/A	N/A	N/A	N/A
D. Hassler, 1990(16)	Clinical manifestation and serology immunofluorescence test	10 d	Adult	Cefotaxime	6g/d	Unspecified	Yes	Penicillin	2×10 megaunits/ d	Unspecified	Yes	N/A	N/A	N/A	N/A
Hans-Walter Pfister, 1989(17)	Clinical manifestation IgM and IgG antibody titers against B. burgdorferi	10 d	Adult	Penicillin	4×5 million U/d	Injection	Yes	Cefotaxime	3×2g/d	Injection	Yes	N/A	N/A	N/A	N/A
Raymond J. Dattwyler, 1997(18)	Clinical manifestation and ELISA or Western blot	14-21 d	Adult	Ceftriaxone	2g/d	Injection	Yes	Doxycycline	200mg/d	Oral	Yes	N/A	N/A	N/A	N/A
Stephen C. Eppes, 2002(19)	Clinical manifestation and ELISA or Western blot	20 d	Children	Amoxicillin	50mg/kg /d	Unspecified	Yes	Cefuroxime	30mg/kg/d	Unspecified	Yes	N/A	N/A	N/A	N/A

B. Barsic, 2000(20)	Clinical manifestation and ELISA or Western blot	5 & 14 d	Adult	Azithromycin	500mg/d	Oral	Yes	Doxycycline	100mg/d	Oral	Yes	N/A	N/A	N/A	N/A
Robert B. Nadelman,1992(21)	Clinical manifestation and ELISA or Western blot	20 d	Adult	Cefuroxime	1g/d	Oral	Yes	Doxycycline	300mg/d	Oral	Yes	N/A	N/A	N/A	N/A
Steven W. Luger, 1995(22)	Clinical manifestation and ELISA or Western blot	20 d	Adult	Cefuroxime	1g/d	Oral	Yes	Doxycycline	300mg/d	Oral	Yes	N/A	N/A	N/A	N/A
M. Karlsson, 1994(23)	Dectect antibody against Borrelia burgdorferi in serum or CSF, culture Borrelia burgdorferi in CSF	14 d	Adult	Penicillin	3g/d	Injection	Yes	Doxycycline	200mg/d	Oral	Yes	N/A	N/A	N/A	N/A
J. Oksi, 1998(24)	Clinical manifestation and ELISA or Western blot	100 d	Unspecified	Cefixime	600mg/d	Oral	Yes	Ceftriaxone+ Amoxicillin	1.5g/d	Oral	Yes	N/A	N/A	N/A	N/A
Knut Eirik Eliassen, 2018(25)	Clinical manifestation and ELISA or Western blot	14 d	Adult	Penicillin	1950mg/d	Oral	Yes	Amoxicillin	1.5g/d	Oral	Yes	Doxycycline	200mg/d	Oral	Yes
John Nowakowski, 1995(26)	Unspecified	30 d	Adult	Tetracycline	2g/d	Oral	Yes	Doxycycline	300mg/d	Oral	Yes	N/A	N/A	N/A	N/A
Gary P. Wormser, 2003(27)	Unspecified	20 d	Adult	Doxycycline+ Ceftriaxone	200mg+ 2g/d	Oral and injection	Yes	Doxycycline	200mg/d	Oral	Yes	N/A	N/A	N/A	N/A
J. Oksi, 2007(28)	Clinical manifestation	121 d	Adult	Ceftriaxone+ Amoxicillin	2g/d	Oral and injection	Yes	Ceftriaxone	Unspecified	Injection	Yes	N/A	N/A	N/A	N/A
Franc Strle, 1992(29)	Clinical manifestation IgM and IgG antibody titers against B. burgdorferi	10 & 14 d	Adult	Doxycycline	200mg/d	Unspecified	Yes	Penicillin	100 million IU tid	Unspecified	Yes	Azithromycin	250mg bid	Oral	Yes
E Breier, 1996(30)	Clinical manifestation IgM and IgG antibody titers against B. burgdorferi	21 d	Adult	Penicillin	1.5 million IU thrice/d	Unspecified	Yes	Minocycline	200mg/d	Unspecified	Yes	N/A	N/A	N/A	N/A
K. Weber, 1993(31)	Unspecified	10 d	Adults	Azithromycin	500mg/d	Orally	Yes	Penicillin	3megaunits/d	Orally	Yes	N/A	N/A	N/A	N/A

Table S2 Characteristics of all included studies

Random effects model:

comparison	k	prop	nma	95%-CI	direct	95%-CI	indir.	95%-CI	RoR	95%-CI	z	p-value
Amoxicillin:Amoxicillin+Probenecid	0	0	1.79	[0.23; 14.02]	.	.	1.79	[0.23; 14.02]	.	.	.	.
Amoxicillin:Azithromycin	1	0.87	2.07	[0.61; 7.02]	2.30	[0.62; 8.50]	1.04	[0.04; 30.44]	2.22	[0.06; 83.30]	0.43	0.6662
Amoxicillin:Cefixime+Probenecid	0	0	3.13	[0.14; 71.94]	.	.	3.13	[0.14; 71.94]	.	.	.	.
Amoxicillin:Cefotaxime	0	0	2.02	[0.34; 11.92]	.	.	2.02	[0.34; 11.92]	.	.	.	.
Amoxicillin:Ceftriaxone	0	0	2.65	[0.53; 13.39]	.	.	2.65	[0.53; 13.39]	.	.	.	.
Amoxicillin:Ceftriaxone+Amoxicillin	0	0	1.45	[0.14; 15.09]	.	.	1.45	[0.14; 15.09]	.	.	.	.
Amoxicillin:Ceftriaxone+Doxycycline	0	0	4.39	[0.63; 30.46]	.	.	4.39	[0.63; 30.46]	.	.	.	.
Amoxicillin:Cefuroxime	0	0	4.34	[0.68; 27.50]	.	.	4.34	[0.68; 27.50]	.	.	.	.
Amoxicillin:Doxycycline	1	0.12	3.79	[0.89; 16.05]	0.93	[0.02; 54.96]	4.63	[0.99; 21.68]	0.20	[0.00; 15.72]	-0.72	0.4699
Amoxicillin:Erythromycin	0	0	2.76	[0.37; 20.67]	.	.	2.76	[0.37; 20.67]	.	.	.	.
Amoxicillin:Minocycline	0	0	5.78	[0.07; 456.38]	.	.	5.78	[0.07; 456.38]	.	.	.	.
Amoxicillin:Penicillin	1	0.19	4.97	[1.15; 21.55]	3.50	[0.12; 104.72]	5.39	[1.06; 27.38]	0.65	[0.01; 28.07]	-0.23	0.8216
Amoxicillin:Tetracycline	0	0	1.54	[0.24; 9.96]	.	.	1.54	[0.24; 9.96]	.	.	.	.
Amoxicillin+Probenecid:Azithromycin	1	0.67	1.16	[0.21; 6.27]	1.23	[0.16; 9.74]	1.02	[0.05; 19.12]	1.20	[0.03; 43.36]	0.10	0.9196
Amoxicillin+Probenecid:Cefixime+Probenecid	0	0	1.75	[0.07; 45.05]	.	.	1.75	[0.07; 45.05]	.	.	.	.
Amoxicillin+Probenecid:Cefotaxime	0	0	1.13	[0.15; 8.33]	.	.	1.13	[0.15; 8.33]	.	.	.	.
Amoxicillin+Probenecid:Ceftriaxone	0	0	1.48	[0.24; 9.24]	.	.	1.48	[0.24; 9.24]	.	.	.	.
Amoxicillin+Probenecid:Ceftriaxone+Amoxicillin	0	0	0.81	[0.07; 9.80]	.	.	0.81	[0.07; 9.80]	.	.	.	.
Amoxicillin+Probenecid:Ceftriaxone+Doxycycline	0	0	2.45	[0.31; 19.36]	.	.	2.45	[0.31; 19.36]	.	.	.	.
Amoxicillin+Probenecid:Cefuroxime	0	0	2.42	[0.33; 17.58]	.	.	2.42	[0.33; 17.58]	.	.	.	.
Amoxicillin+Probenecid:Doxycycline	2	0.90	2.12	[0.42; 10.62]	2.15	[0.39; 11.79]	1.88	[0.01; 281.89]	1.14	[0.01; 227.69]	0.05	0.9604
Amoxicillin+Probenecid:Erythromycin	0	0	1.54	[0.17; 14.08]	.	.	1.54	[0.17; 14.08]	.	.	.	.
Amoxicillin+Probenecid:Minocycline	0	0	3.23	[0.04; 281.80]	.	.	3.23	[0.04; 281.80]	.	.	.	.
Amoxicillin+Probenecid:Penicillin	0	0	2.78	[0.49; 15.88]	.	.	2.78	[0.49; 15.88]	.	.	.	.
Amoxicillin+Probenecid:Tetracycline	0	0	0.86	[0.11; 6.80]	.	.	0.86	[0.11; 6.80]	.	.	.	.
Azithromycin:Cefixime+Probenecid	0	0	1.51	[0.08; 28.03]	.	.	1.51	[0.08; 28.03]	.	.	.	.
Azithromycin:Cefotaxime	0	0	0.98	[0.25; 3.83]	.	.	0.98	[0.25; 3.83]	.	.	.	.
Azithromycin:Ceftriaxone	0	0	1.28	[0.40; 4.05]	.	.	1.28	[0.40; 4.05]	.	.	.	.
Azithromycin:Ceftriaxone+Amoxicillin	0	0	0.70	[0.09; 5.42]	.	.	0.70	[0.09; 5.42]	.	.	.	.
Azithromycin:Ceftriaxone+Doxycycline	0	0	2.12	[0.45; 10.06]	.	.	2.12	[0.45; 10.06]	.	.	.	.
Azithromycin:Cefuroxime	0	0	2.09	[0.49; 8.87]	.	.	2.09	[0.49; 8.87]	.	.	.	.
Azithromycin:Doxycycline	4	0.75	1.83	[0.77; 4.37]	2.90	[1.06; 7.95]	0.47	[0.08; 2.64]	6.21	[0.84; 46.01]	1.79	0.0740
Azithromycin:Erythromycin	0	0	1.33	[0.25; 7.05]	.	.	1.33	[0.25; 7.05]	.	.	.	.
Azithromycin:Minocycline	0	0	2.79	[0.04; 189.88]	.	.	2.79	[0.04; 189.88]	.	.	.	.
Azithromycin:Penicillin	2	0.57	2.40	[0.94; 6.13]	1.49	[0.43; 5.16]	4.51	[1.08; 18.81]	0.33	[0.05; 2.20]	-1.14	0.2525
Azithromycin:Tetracycline	0	0	0.75	[0.17; 3.27]	.	.	0.75	[0.17; 3.27]	.	.	.	.
Cefixime+Probenecid:Cefotaxime	0	0	0.65	[0.03; 12.16]	.	.	0.65	[0.03; 12.16]	.	.	.	.
Cefixime+Probenecid:Ceftriaxone	0	0	0.85	[0.06; 12.40]	.	.	0.85	[0.06; 12.40]	.	.	.	.
Cefixime+Probenecid:Ceftriaxone+Amoxicillin	1	1.00	0.46	[0.06; 3.74]	0.46	[0.06; 3.74]	.	.	.	.	.	.
Cefixime+Probenecid:Ceftriaxone+Doxycycline	0	0	1.40	[0.06; 31.65]	.	.	1.40	[0.06; 31.65]	.	.	.	.
Cefixime+Probenecid:Cefuroxime	0	0	1.38	[0.06; 29.59]	.	.	1.38	[0.06; 29.59]	.	.	.	.
Cefixime+Probenecid:Doxycycline	0	0	1.21	[0.07; 20.65]	.	.	1.21	[0.07; 20.65]	.	.	.	.
Cefixime+Probenecid:Erythromycin	0	0	0.88	[0.04; 20.43]	.	.	0.88	[0.04; 20.43]	.	.	.	.
Cefixime+Probenecid:Minocycline	0	0	1.85	[0.01; 271.09]	.	.	1.85	[0.01; 271.09]	.	.	.	.
Cefixime+Probenecid:Penicillin	0	0	1.59	[0.09; 26.67]	.	.	1.59	[0.09; 26.67]	.	.	.	.
Cefixime+Probenecid:Tetracycline	0	0	0.49	[0.02; 10.45]	.	.	0.49	[0.02; 10.45]	.	.	.	.
Cefotaxime:Ceftriaxone	1	0.38	1.31	[0.40; 4.31]	0.75	[0.11; 5.12]	1.86	[0.41; 8.42]	0.40	[0.04; 4.66]	-0.73	0.4680
Cefotaxime:Ceftriaxone+Amoxicillin	0	0	0.72	[0.09; 5.67]	.	.	0.72	[0.09; 5.67]	.	.	.	.
Cefotaxime:Ceftriaxone+Doxycycline	0	0	2.17	[0.36; 12.99]	.	.	2.17	[0.36; 12.99]	.	.	.	.
Cefotaxime:Cefuroxime	0	0	2.14	[0.40; 11.64]	.	.	2.14	[0.40; 11.64]	.	.	.	.
Cefotaxime:Doxycycline	0	0	1.87	[0.54; 6.46]	.	.	1.87	[0.54; 6.46]	.	.	.	.
Cefotaxime:Erythromycin	0	0	1.36	[0.24; 7.82]	.	.	1.36	[0.24; 7.82]	.	.	.	.
Cefotaxime:Minocycline	0	0	2.86	[0.04; 199.21]	.	.	2.86	[0.04; 199.21]	.	.	.	.
Cefotaxime:Penicillin	2	0.77	2.46	[0.87; 6.94]	3.04	[0.93; 9.96]	1.23	[0.14; 10.45]	2.47	[0.21; 28.54]	0.73	0.4680
Cefotaxime:Tetracycline	0	0	0.76	[0.16; 3.76]	.	.	0.76	[0.16; 3.76]	.	.	.	.
Ceftriaxone:Ceftriaxone+Amoxicillin	1	1.00	0.55	[0.10; 2.97]	0.55	[0.10; 2.97]	.	.	.	.	.	.
Ceftriaxone:Ceftriaxone+Doxycycline	0	0	1.65	[0.34; 8.08]	.	.	1.65	[0.34; 8.08]	.	.	.	.
Ceftriaxone:Cefuroxime	0	0	1.63	[0.37; 7.14]	.	.	1.63	[0.37; 7.14]	.	.	.	.
Ceftriaxone:Doxycycline	2	0.55	1.43	[0.57; 3.58]	0.59	[0.17; 2.02]	4.30	[1.09; 17.04]	0.14	[0.02; 0.87]	-2.11	0.0349
Ceftriaxone:Erythromycin	0	0	1.04	[0.20; 5.35]	.	.	1.04	[0.20; 5.35]	.	.	.	.
Ceftriaxone:Minocycline	0	0	2.18	[0.03; 146.21]	.	.	2.18	[0.03; 146.21]	.	.	.	.
Ceftriaxone:Penicillin	3	0.52	1.88	[0.79; 4.47]	3.49	[1.05; 11.59]	0.95	[0.27; 3.34]	3.68	[0.65; 20.90]	1.47	0.1420
Ceftriaxone:Tetracycline	0	0	0.58	[0.14; 2.50]	.	.	0.58	[0.14; 2.50]	.	.	.	.
Ceftriaxone+Amoxicillin:Ceftriaxone+Doxycycline	0	0	3.02	[0.30; 30.61]	.	.	3.02	[0.30; 30.61]	.	.	.	.
Ceftriaxone+Amoxicillin:Cefuroxime	0	0	2.98	[0.32; 28.07]	.	.	2.98	[0.32; 28.07]	.	.	.	.
Ceftriaxone+Amoxicillin:Doxycycline	0	0	2.61	[0.38; 17.83]	.	.	2.61	[0.38; 17.83]	.	.	.	.
Ceftriaxone+Amoxicillin:Erythromycin	0	0	1.90	[0.18; 19.94]	.	.	1.90	[0.18; 19.94]	.	.	.	.
Ceftriaxone+Amoxicillin:Minocycline	0	0	3.98	[0.04; 369.76]	.	.	3.98	[0.04; 369.76]	.	.	.	.
Ceftriaxone+Amoxicillin:Penicillin	0	0	3.42	[0.51; 22.86]	.	.	3.42	[0.51; 22.86]	.	.	.	.
Ceftriaxone+Amoxicillin:Tetracycline	0	0	1.06	[0.11; 9.88]	.	.	1.06	[0.11; 9.88]	.	.	.	.
Ceftriaxone+Doxycycline:Cefuroxime	0	0	0.99	[0.17; 5.58]	.	.	0.99	[0.17; 5.58]	.	.	.	.
Ceftriaxone+Doxycycline:Doxycycline	1	1.00	0.86	[0.24; 3.14]	0.86	[0.24; 3.14]	.	.	.	.	.	.
Ceftriaxone+Doxycycline:Erythromycin	0	0	0.63	[0.08; 4.76]	.	.	0.63	[0.08; 4.76]	.	.	.	.
Ceftriaxone+Doxycycline:Minocycline	0	0	1.32	[0.02; 105.38]	.	.	1.32	[0.02; 105.38]	.	.	.	.
Ceftriaxone+Doxycycline:Penicillin	0	0	1.13	[0.25; 5.11]	.	.	1.13	[0.25; 5.11]	.	.	.	.
Ceftriaxone+Doxycycline:Tetracycline	0	0	0.35	[0.05; 2.26]	.	.	0.35	[0.05; 2.26]	.	.	.	.
Cefuroxime:Doxycycline	2	1.00	0.87	[0.28; 2.77]	0.87	[0.28; 2.77]	.	.	.	.	.	.
Cefuroxime:Erythromycin	0	0	0.64	[0.09; 4.42]	.	.	0.64	[0.09; 4.42]	.	.	.	.
Cefuroxime:Minocycline	0	0	1.33	[0.02; 102.57]	.	.	1.33	[0.02; 102.57]	.	.	.	.
Cefuroxime:Penicillin	0	0	1.15	[0.29; 4.59]	.	.	1.15	[0.29; 4.59]	.	.	.	.
Cefuroxime:Tetracycline	0	0	0.36	[0.06; 2.08]	.	.	0.36	[0.06; 2.08]	.	.	.	.
Doxycycline:Erythromycin	0	0	0.73	[0.15; 3.46]	.	.	0.73	[0.15; 3.46]	.	.	.	.
Doxycycline:Minocycline	0	0	1.53	[0.02; 100.43]	.	.	1.53	[0.02; 100.43]	.	.	.	.
Doxycycline:Penicillin	4	0.54	1.31	[0.61; 2.84]	1.51	[0.53; 4.31]	1.11	[0.36; 3.48]	1.36	[0.29; 6.40]	0.39	0.6983
Doxycycline:Tetracycline	1	0.32	0.41	[0.11; 1.55]	0.10	[0.01; 1.04]	0.80	[0.16; 4.04]	0.13	[0.01; 2.17]	-1.43	0.1534
Erythromycin:Minocycline	0	0	2.10	[0.03; 162.51]	.	.	2.10	[0.03; 162.51]	.	.	.	.
Erythromycin:Penicillin	1	0.94	1.80	[0.44; 7.40]	1.40	[0.33; 5.99]	117.69	[0.32; 43313.14]	0.01	[0.00; 5.22]	-1.43	0.1534
Erythromycin:Tetracycline	1	0.94	0.56	[0.14; 2.30]	0.72	[0.17; 3.09]	0.01	[0.00; 3.16]	83.98	[0.19; 36814.68]	1.43	0.1534
Minocycline:Penicillin	1	1.00	0.86	[0.01; 52.72]	0.86	[0.01; 52.72]	.	.	.	.	.	.
Minocycline:Tetracycline	0	0	0.27	[0.00; 19.57]	.	.	0.27	[0.00; 19.57]	.	.	.	.
Penicillin:Tetracycline	1	0.76	0.31	[0.09; 1.06]	0.52	[0.13; 2.11]	0.06	[0.01; 0.77]	8.00	[0.46; 138.93]	1.43	0.1534

Legend:
comparison - Treatment comparison
k - Number of studies providing direct evidence
prop - Direct evidence proportion
nma - Estimated treatment effect (OR) in network meta-analysis
direct - Estimated treatment effect (OR) derived from direct evidence
indir. - Estimated treatment effect (OR) derived from indirect evidence
RoR - Ratio of Ratios (direct versus indirect)
z - z-value of test for disagreement (direct versus indirect)
p-value - p-value of test for disagreement (direct versus indirect)
>

FIG S1 Pairwise comparison and inconsistency test of efficacy for antibiotics treating LD

Random effects model:

comparison	k	prop	nma	95%-CI	direct	95%-CI	indir.	95%-CI	RoR	95%-CI	z	p-value
Amoxicillin(1.5g):Amoxicillin(1.5g)+Probenecid(1.5g)	0	0	3.56	[0.50; 25.62]	.	.	3.56	[0.50; 25.62]	.	.	.	.
Amoxicillin(1.5g):Azithromycin(0.25g)	0	0	4.04	[0.68; 23.83]	.	.	4.04	[0.68; 23.83]	.	.	.	.
Amoxicillin(1.5g):Azithromycin(0.5g)	1	0.97	2.14	[1.05; 4.36]	2.30	[1.11; 4.75]	0.20	[0.00; 12.39]	11.76	[0.17; 794.06]	1.15	0.2514
Amoxicillin(1.5g):Cefotaxime(6g)	0	0	18.94	[1.99; 180.18]	.	.	18.94	[1.99; 180.18]	.	.	.	.
Amoxicillin(1.5g):Ceftriaxone(1g)	0	0	0.75	[0.12; 4.89]	.	.	0.75	[0.12; 4.89]	.	.	.	.
Amoxicillin(1.5g):Ceftriaxone(2g)	0	0	14.20	[2.86; 70.51]	.	.	14.20	[2.86; 70.51]	.	.	.	.
Amoxicillin(1.5g):Ceftriaxone(2g)+Amoxicillin(2g)	0	0	7.78	[0.99; 60.98]	.	.	7.78	[0.99; 60.98]	.	.	.	.
Amoxicillin(1.5g):Doxycycline(0.1g)	0	0	71.85	[5.08; 1016.43]	.	.	71.85	[5.08; 1016.43]	.	.	.	.
Amoxicillin(1.5g):Doxycycline(0.2g)	1	0.13	7.97	[1.96; 32.44]	0.93	[0.02; 47.42]	10.91	[2.43; 49.03]	0.09	[0.00; 5.74]	-1.15	0.2514
Amoxicillin(1.5g):Penicillin(12g)	0	0	10.68	[0.16; 710.73]	.	.	10.68	[0.16; 710.73]	.	.	.	.
Amoxicillin(1.5g):Penicillin(1megaunit)	0	0	17.02	[2.67; 108.31]	.	.	17.02	[2.67; 108.31]	.	.	.	.
Amoxicillin(1.5g):Penicillin(20megaunits)	0	0	67.20	[6.13; 736.85]	.	.	67.20	[6.13; 736.85]	.	.	.	.
Amoxicillin(1.5g):Penicillin(3.9g)	1	0.67	10.25	[0.73; 144.34]	3.50	[0.14; 87.57]	95.03	[0.92; 9781.53]	0.04	[0.00; 10.39]	-1.15	0.2514
Amoxicillin(1.5g):Penicillin(3megaunits)	0	0	1.36	[0.32; 5.84]	.	.	1.36	[0.32; 5.84]	.	.	.	.
Amoxicillin(1.5g)+Probenecid(1.5g):Azithromycin(0.25g)	1	0.77	1.13	[0.24; 5.33]	1.23	[0.21; 7.15]	0.86	[0.03; 22.05]	1.44	[0.04; 57.87]	0.19	0.8472
Amoxicillin(1.5g)+Probenecid(1.5g):Azithromycin(0.5g)	0	0	0.60	[0.09; 3.88]	.	.	0.60	[0.09; 3.88]	.	.	.	.
Amoxicillin(1.5g)+Probenecid(1.5g):Cefotaxime(6g)	0	0	5.31	[0.57; 49.98]	.	.	5.31	[0.57; 49.98]	.	.	.	.
Amoxicillin(1.5g)+Probenecid(1.5g):Ceftriaxone(1g)	0	0	0.21	[0.02; 2.69]	.	.	0.21	[0.02; 2.69]	.	.	.	.
Amoxicillin(1.5g)+Probenecid(1.5g):Ceftriaxone(2g)	0	0	3.99	[0.82; 19.47]	.	.	3.99	[0.82; 19.47]	.	.	.	.
Amoxicillin(1.5g)+Probenecid(1.5g):Ceftriaxone(2g)+Amoxicillin(2g)	0	0	2.18	[0.28; 16.90]	.	.	2.18	[0.28; 16.90]	.	.	.	.
Amoxicillin(1.5g)+Probenecid(1.5g):Doxycycline(0.1g)	0	0	20.16	[1.44; 282.43]	.	.	20.16	[1.44; 282.43]	.	.	.	.
Amoxicillin(1.5g)+Probenecid(1.5g):Doxycycline(0.2g)	2	0.95	2.24	[0.56; 8.94]	2.23	[0.54; 9.23]	2.41	[0.01; 1087.63]	0.92	[0.00; 490.68]	-0.02	0.9802
Amoxicillin(1.5g)+Probenecid(1.5g):Penicillin(12g)	0	0	3.00	[0.05; 198.21]	.	.	3.00	[0.05; 198.21]	.	.	.	.
Amoxicillin(1.5g)+Probenecid(1.5g):Penicillin(1megaunit)	0	0	4.78	[0.82; 27.90]	.	.	4.78	[0.82; 27.90]	.	.	.	.
Amoxicillin(1.5g)+Probenecid(1.5g):Penicillin(20megaunits)	0	0	18.86	[1.74; 204.53]	.	.	18.86	[1.74; 204.53]	.	.	.	.
Amoxicillin(1.5g)+Probenecid(1.5g):Penicillin(3.9g)	0	0	2.88	[0.15; 56.95]	.	.	2.88	[0.15; 56.95]	.	.	.	.
Amoxicillin(1.5g)+Probenecid(1.5g):Penicillin(3megaunits)	0	0	0.38	[0.04; 3.65]	.	.	0.38	[0.04; 3.65]	.	.	.	.
Azithromycin(0.25g):Azithromycin(0.5g)	0	0	0.53	[0.10; 2.77]	.	.	0.53	[0.10; 2.77]	.	.	.	.
Azithromycin(0.25g):Cefotaxime(6g)	0	0	4.69	[0.59; 37.14]	.	.	4.69	[0.59; 37.14]	.	.	.	.
Azithromycin(0.25g):Ceftriaxone(1g)	0	0	0.19	[0.02; 2.04]	.	.	0.19	[0.02; 2.04]	.	.	.	.
Azithromycin(0.25g):Ceftriaxone(2g)	0	0	3.52	[0.93; 13.33]	.	.	3.52	[0.93; 13.33]	.	.	.	.
Azithromycin(0.25g):Ceftriaxone(2g)+Amoxicillin(2g)	0	0	1.93	[0.30; 12.33]	.	.	1.93	[0.30; 12.33]	.	.	.	.
Azithromycin(0.25g):Doxycycline(0.1g)	0	0	17.79	[1.47; 215.73]	.	.	17.79	[1.47; 215.73]	.	.	.	.
Azithromycin(0.25g):Doxycycline(0.2g)	2	0.99	1.97	[0.67; 5.84]	2.01	[0.68; 5.98]	0.16	[0.00; 39706.49]	12.55	[0.00; 3260978.07]	0.40	0.6909
Azithromycin(0.25g):Penicillin(12g)	0	0	2.64	[0.04; 159.94]	.	.	2.64	[0.04; 159.94]	.	.	.	.
Azithromycin(0.25g):Penicillin(1megaunit)	1	0.79	4.21	[1.11; 15.95]	4.25	[0.95; 19.07]	4.08	[0.23; 72.75]	1.04	[0.04; 26.84]	0.02	0.9802
Azithromycin(0.25g):Penicillin(20megaunits)	0	0	16.64	[1.80; 153.67]	.	.	16.64	[1.80; 153.67]	.	.	.	.
Azithromycin(0.25g):Penicillin(3.9g)	0	0	2.54	[0.15; 44.27]	.	.	2.54	[0.15; 44.27]	.	.	.	.
Azithromycin(0.25g):Penicillin(3megaunits)	0	0	0.34	[0.04; 2.72]	.	.	0.34	[0.04; 2.72]	.	.	.	.
Azithromycin(0.5g):Cefotaxime(6g)	0	0	8.86	[1.02; 76.87]	.	.	8.86	[1.02; 76.87]	.	.	.	.
Azithromycin(0.5g):Ceftriaxone(1g)	0	0	0.35	[0.06; 1.99]	.	.	0.35	[0.06; 1.99]	.	.	.	.
Azithromycin(0.5g):Ceftriaxone(2g)	0	0	6.64	[1.53; 28.90]	.	.	6.64	[1.53; 28.90]	.	.	.	.
Azithromycin(0.5g):Ceftriaxone(2g)+Amoxicillin(2g)	0	0	3.64	[0.51; 25.78]	.	.	3.64	[0.51; 25.78]	.	.	.	.
Azithromycin(0.5g):Doxycycline(0.1g)	0	0	33.61	[2.57; 439.84]	.	.	33.61	[2.57; 439.84]	.	.	.	.
Azithromycin(0.5g):Doxycycline(0.2g)	2	0.90	3.73	[1.07; 13.02]	4.74	[1.27; 17.70]	0.40	[0.01; 22.03]	11.76	[0.17; 794.06]	1.15	0.2514
Azithromycin(0.5g):Penicillin(12g)	0	0	5.00	[0.08; 316.66]	.	.	5.00	[0.08; 316.66]	.	.	.	.
Azithromycin(0.5g):Penicillin(1megaunit)	0	0	7.96	[1.40; 45.24]	.	.	7.96	[1.40; 45.24]	.	.	.	.
Azithromycin(0.5g):Penicillin(20megaunits)	0	0	31.44	[3.13; 316.13]	.	.	31.44	[3.13; 316.13]	.	.	.	.
Azithromycin(0.5g):Penicillin(3.9g)	0	0	4.80	[0.34; 68.18]	.	.	4.80	[0.34; 68.18]	.	.	.	.
Azithromycin(0.5g):Penicillin(3megaunits)	1	1.00	0.64	[0.18; 2.27]	0.64	[0.18; 2.27]	.	.	.	.	.	.
Cefotaxime(6g):Ceftriaxone(1g)	0	0	0.04	[0.00; 0.63]	.	.	0.04	[0.00; 0.63]	.	.	.	.
Cefotaxime(6g):Ceftriaxone(2g)	1	1.00	0.75	[0.15; 3.65]	0.75	[0.15; 3.65]	.	.	.	.	.	.
Cefotaxime(6g):Ceftriaxone(2g)+Amoxicillin(2g)	0	0	0.41	[0.05; 3.17]	.	.	0.41	[0.05; 3.17]	.	.	.	.
Cefotaxime(6g):Doxycycline(0.1g)	0	0	3.79	[0.94; 15.30]	.	.	3.79	[0.94; 15.30]	.	.	.	.
Cefotaxime(6g):Doxycycline(0.2g)	0	0	0.42	[0.07; 2.45]	.	.	0.42	[0.07; 2.45]	.	.	.	.
Cefotaxime(6g):Penicillin(12g)	0	0	0.56	[0.01; 42.86]	.	.	0.56	[0.01; 42.86]	.	.	.	.
Cefotaxime(6g):Penicillin(1megaunit)	0	0	0.90	[0.11; 7.60]	.	.	0.90	[0.11; 7.60]	.	.	.	.
Cefotaxime(6g):Penicillin(20megaunits)	2	1.00	3.55	[1.58; 7.99]	3.55	[1.58; 7.99]	.	.	.	.	.	.
Cefotaxime(6g):Penicillin(3.9g)	0	0	0.54	[0.02; 12.99]	.	.	0.54	[0.02; 12.99]	.	.	.	.
Cefotaxime(6g):Penicillin(3megaunits)	0	0	0.07	[0.01; 0.88]	.	.	0.07	[0.01; 0.88]	.	.	.	.
Ceftriaxone(1g):Ceftriaxone(2g)	0	0	18.89	[1.95; 182.97]	.	.	18.89	[1.95; 182.97]	.	.	.	.
Ceftriaxone(1g):Ceftriaxone(2g)+Amoxicillin(2g)	0	0	10.35	[0.76; 141.16]	.	.	10.35	[0.76; 141.16]	.	.	.	.
Ceftriaxone(1g):Doxycycline(0.1g)	0	0	95.56	[4.31; 2120.64]	.	.	95.56	[4.31; 2120.64]	.	.	.	.
Ceftriaxone(1g):Doxycycline(0.2g)	0	0	10.60	[1.25; 89.66]	.	.	10.60	[1.25; 89.66]	.	.	.	.
Ceftriaxone(1g):Penicillin(12g)	0	0	14.20	[0.16; 1273.13]	.	.	14.20	[0.16; 1273.13]	.	.	.	.
Ceftriaxone(1g):Penicillin(1megaunit)	0	0	22.63	[1.95; 262.89]	.	.	22.63	[1.95; 262.89]	.	.	.	.
Ceftriaxone(1g):Penicillin(20megaunits)	0	0	89.38	[4.99; 1600.16]	.	.	89.38	[4.99; 1600.16]	.	.	.	.
Ceftriaxone(1g):Penicillin(3.9g)	0	0	13.63	[0.57; 324.20]	.	.	13.63	[0.57; 324.20]	.	.	.	.
Ceftriaxone(1g):Penicillin(3megaunits)	1	1.00	1.81	[0.56; 5.89]	1.81	[0.56; 5.89]	.	.	.	.	.	.
Ceftriaxone(2g):Ceftriaxone(2g)+Amoxicillin(2g)	1	1.00	0.55	[0.15; 2.00]	0.55	[0.15; 2.00]	.	.	.	.	.	.
Ceftriaxone(2g):Doxycycline(0.1g)	0	0	5.06	[0.61; 41.73]	.	.	5.06	[0.61; 41.73]	.	.	.	.
Ceftriaxone(2g):Doxycycline(0.2g)	2	1.00	0.56	[0.26; 1.21]	0.56	[0.26; 1.21]	.	.	.	.	.	.
Ceftriaxone(2g):Penicillin(12g)	0	0	0.75	[0.01; 42.34]	.	.	0.75	[0.01; 42.34]	.	.	.	.
Ceftriaxone(2g):Penicillin(1megaunit)	0	0	1.20	[0.29; 5.02]	.	.	1.20	[0.29; 5.02]	.	.	.	.
Ceftriaxone(2g):Penicillin(20megaunits)	0	0	4.73	[0.80; 28.05]	.	.	4.73	[0.80; 28.05]	.	.	.	.
Ceftriaxone(2g):Penicillin(3.9g)	0	0	0.72	[0.05; 11.35]	.	.	0.72	[0.05; 11.35]	.	.	.	.
Ceftriaxone(2g):Penicillin(3megaunits)	0	0	0.10	[0.01; 0.67]	.	.	0.10	[0.01; 0.67]	.	.	.	.
Ceftriaxone(2g)+Amoxicillin(2g):Doxycycline(0.1g)	0	0	9.23	[0.78; 109.57]	.	.	9.23	[0.78; 109.57]	.	.	.	.
Ceftriaxone(2g)+Amoxicillin(2g):Doxycycline(0.2g)	0	0	1.02	[0.23; 4.61]	.	.	1.02	[0.23; 4.61]	.	.	.	.
Ceftriaxone(2g)+Amoxicillin(2g):Penicillin(12g)	0	0	1.37	[0.02; 94.53]	.	.	1.37	[0.02; 94.53]	.	.	.	.
Ceftriaxone(2g)+Amoxicillin(2g):Penicillin(1megaunit)	0	0	2.19	[0.32; 15.04]	.	.	2.19	[0.32; 15.04]	.	.	.	.
Ceftriaxone(2g)+Amoxicillin(2g):Penicillin(20megaunits)	0	0	8.63	[0.96; 77.84]	.	.	8.63	[0.96; 77.84]	.	.	.	.
Ceftriaxone(2g)+Amoxicillin(2g):Penicillin(3.9g)	0	0	1.32	[0.06; 27.61]	.	.	1.32	[0.06; 27.61]	.	.	.	.
Ceftriaxone(2g)+Amoxicillin(2g):Penicillin(3megaunits)	0	0	0.18	[0.02; 1.80]	.	.	0.18	[0.02; 1.80]	.	.	.	.
Doxycycline(0.1g):Doxycycline(0.2g)	0	0	0.11	[0.01; 1.05]	.	.	0.11	[0.01; 1.05]	.	.	.	.
Doxycycline(0.1g):Penicillin(12g)	0	0	0.15	[0.00; 14.06]	.	.	0.15	[0.00; 14.06]	.	.	.	.
Doxycycline(0.1g):Penicillin(1megaunit)	0	0	0.24	[0.02; 3.03]	.	.	0.24	[0.02; 3.03]	.	.	.	.
Doxycycline(0.1g):Penicillin(20megaunits)	1	1.00	0.94	[0.30; 2.91]	0.94	[0.30; 2.91]	.	.	.	.	.	.
Doxycycline(0.1g):Penicillin(3.9g)	0	0	0.14	[0.00; 4.59]	.	.	0.14	[0.00; 4.59]	.	.	.	.
Doxycycline(0.1g):Penicillin(3megaunits)	0	0	0.02	[0.00; 0.33]	.	.	0.02	[0.00; 0.33]	.	.	.	.
Doxycycline(0.2g):Penicillin(12g)	1	1.00	1.34	[0.03; 70.05]	1.34	[0.03; 70.05]	.	.	.	.	.	.
Doxycycline(0.2g):Penicillin(1megaunit)	1	0.90	2.14	[0.64; 7.13]	2.12	[0.60; 7.57]	2.24	[0.05; 104.09]	0.95	[0.02; 54.25]	-0.02	0.9802
Doxycycline(0.2g):Penicillin(20megaunits)	0	0	8.43	[1.21; 58.68]	.	.	8.43	[1.21; 58.68]	.	.	.	.
Doxycycline(0.2g):Penicillin(3.9g)	1	0.67	1.29	[0.09; 18.11]	3.77	[0.15; 94.40]	0.14	[0.00; 14.29]	27.20	[0.10; 7689.14]	1.15	0.2514
Doxycycline(0.2g):Penicillin(3megaunits)	0	0	0.17	[0.03; 1.02]	.	.	0.17	[0.03; 1.02]	.	.	.	.
Penicillin(12g):Penicillin(1megaunit)	0	0	1.59	[0.03; 99.65]	.	.	1.59	[0.03; 99.65]	.	.	.	.
Penicillin(12g):Penicillin(20megaunits)	0	0	6.29	[0.08; 515.70]	.	.	6.29	[0.08; 515.70]	.	.	.	.
Penicillin(12g):Penicillin(3.9g)	0	0	0.96	[0.01; 111.92]	.	.	0.96	[0.01; 111.92]	.	.	.	.
Penicillin(12g):Penicillin(3megaunits)	0	0	0.13	[0.00; 9.78]	.	.	0.13	[0.00; 9.78]	.	.	.	.
Penicillin(1megaunit):Penicillin(20megaunits)	0	0	3.95	[0.40; 38.77]	.	.	3.95	[0.40; 38.77]	.	.	.	.
Penicillin(1megaunit):Penicillin(3.9g)	0	0	0.60	[0.03; 11.02]	.	.	0.60	[0.03; 11.02]	.	.	.	.
Penicillin(1megaunit):Penicillin(3megaunits)	0	0	0.08	[0.01; 0.69]	.	.	0.08	[0.01; 0.69]	.	.	.	.
Penicillin(20megaunits):Penicillin(3.9g)	0	0	0.15	[0.01; 4.05]	.	.	0.15	[0.01; 4.05]	.	.	.	.
Penicillin(20megaunits):Penicillin(3megaunits)	0	0	0.02	[0.00; 0.28]	.	.	0.02	[0.00; 0.28]	.	.	.	.
Penicillin(3.9g):Penicillin(3megaunits)	0	0	0.13	[0.01; 2.52]	.	.	0.13	[0.01; 2.52]	.	.	.	.

Legend:
- comparison - Treatment comparison
- k - Number of studies providing direct evidence
- prop - Direct evidence proportion
- nma - Estimated treatment effect (OR) in network meta-analysis
- direct - Estimated treatment effect (OR) derived from direct evidence
- indir. - Estimated treatment effect (OR) derived from indirect evidence
- RoR - Ratio of Ratios (direct versus indirect)
- z - z-value of test for disagreement (direct versus indirect)
- p-value - p-value of test for disagreement (direct versus indirect)

FIG S2 Pairwise comparison and inconsistency test of efficacy for different daily dosages of antibiotics treating LD

Random effects model:

comparison	k	prop	nma	95%-CI	direct	95%-CI	indir.	95%-CI	RoR	95%-CI	z	p-value
Amoxicillin:Amoxicillin+Probenecid	0	0	0.31	[0.09; 1.06]	.	.	0.31	[0.09; 1.06]	.	.	.	.
Amoxicillin:Azithromycin	1	0.72	0.71	[0.44; 1.13]	0.59	[0.34; 1.02]	1.15	[0.46; 2.83]	0.51	[0.18; 1.48]	-1.23	0.2169
Amoxicillin:Cefixime+Probenecid	0	0	1.44	[0.19; 11.08]	.	.	1.44	[0.19; 11.08]	.	.	.	.
Amoxicillin:Cefotaxime	0	0	1.51	[0.37; 6.10]	.	.	1.51	[0.37; 6.10]	.	.	.	.
Amoxicillin:Ceftriaxone	0	0	0.59	[0.28; 1.21]	.	.	0.59	[0.28; 1.21]	.	.	.	.
Amoxicillin:Ceftriaxone+Amoxicillin	0	0	0.67	[0.25; 1.82]	.	.	0.67	[0.25; 1.82]	.	.	.	.
Amoxicillin:Ceftriaxone+Doxycycline	0	0	0.45	[0.20; 1.05]	.	.	0.45	[0.20; 1.05]	.	.	.	.
Amoxicillin:Cefuroxime	0	0	1.47	[0.70; 3.06]	.	.	1.47	[0.70; 3.06]	.	.	.	.
Amoxicillin:Doxycycline	1	0.64	0.96	[0.55; 1.66]	1.39	[0.70; 2.78]	0.49	[0.20; 1.22]	2.85	[0.91; 8.96]	1.79	0.0735
Amoxicillin:Minocycline	0	0	0.16	[0.03; 0.77]	.	.	0.16	[0.03; 0.77]	.	.	.	.
Amoxicillin:Penicillin	1	0.68	1.34	[0.74; 2.43]	1.38	[0.67; 2.84]	1.27	[0.45; 3.63]	1.08	[0.30; 3.86]	0.12	0.9067
Amoxicillin:Tetracycline	0	0	0.71	[0.24; 2.12]	.	.	0.71	[0.24; 2.12]	.	.	.	.
Amoxicillin+Probenecid:Azithromycin	1	0.60	2.29	[0.69; 7.61]	3.15	[0.67; 14.86]	1.42	[0.21; 9.48]	2.21	[0.19; 25.63]	0.64	0.5251
Amoxicillin+Probenecid:Cefixime+Probenecid	0	0	4.68	[0.47; 46.52]	.	.	4.68	[0.47; 46.52]	.	.	.	.
Amoxicillin+Probenecid:Cefotaxime	0	0	4.90	[0.82; 29.15]	.	.	4.90	[0.82; 29.15]	.	.	.	.
Amoxicillin+Probenecid:Ceftriaxone	0	0	1.91	[0.53; 6.86]	.	.	1.91	[0.53; 6.86]	.	.	.	.
Amoxicillin+Probenecid:Ceftriaxone+Amoxicillin	0	0	2.17	[0.51; 9.29]	.	.	2.17	[0.51; 9.29]	.	.	.	.
Amoxicillin+Probenecid:Ceftriaxone+Doxycycline	0	0	1.48	[0.39; 5.66]	.	.	1.48	[0.39; 5.66]	.	.	.	.
Amoxicillin+Probenecid:Cefuroxime	0	0	4.77	[1.32; 17.17]	.	.	4.77	[1.32; 17.17]	.	.	.	.
Amoxicillin+Probenecid:Doxycycline	2	0.71	3.11	[0.95; 10.17]	2.95	[0.72; 12.02]	3.53	[0.39; 32.26]	0.84	[0.06; 11.49]	-0.13	0.8936
Amoxicillin+Probenecid:Minocycline	0	0	0.51	[0.07; 3.56]	.	.	0.51	[0.07; 3.56]	.	.	.	.
Amoxicillin+Probenecid:Penicillin	0	0	4.36	[1.22; 15.52]	.	.	4.36	[1.22; 15.52]	.	.	.	.
Amoxicillin+Probenecid:Tetracycline	0	0	2.32	[0.51; 10.51]	.	.	2.32	[0.51; 10.51]	.	.	.	.
Azithromycin:Cefixime+Probenecid	0	0	2.04	[0.26; 15.78]	.	.	2.04	[0.26; 15.78]	.	.	.	.
Azithromycin:Cefotaxime	0	0	2.14	[0.52; 8.74]	.	.	2.14	[0.52; 8.74]	.	.	.	.
Azithromycin:Ceftriaxone	0	0	0.83	[0.40; 1.74]	.	.	0.83	[0.40; 1.74]	.	.	.	.
Azithromycin:Ceftriaxone+Amoxicillin	0	0	0.95	[0.35; 2.60]	.	.	0.95	[0.35; 2.60]	.	.	.	.
Azithromycin:Ceftriaxone+Doxycycline	0	0	0.64	[0.28; 1.50]	.	.	0.64	[0.28; 1.50]	.	.	.	.
Azithromycin:Cefuroxime	0	0	2.08	[0.99; 4.38]	.	.	2.08	[0.99; 4.38]	.	.	.	.
Azithromycin:Doxycycline	4	0.46	1.36	[0.77; 2.38]	0.76	[0.33; 1.74]	2.24	[1.04; 4.84]	0.34	[0.11; 1.05]	-1.88	0.0604
Azithromycin:Minocycline	0	0	0.22	[0.05; 1.10]	.	.	0.22	[0.05; 1.10]	.	.	.	.
Azithromycin:Penicillin	2	0.34	1.90	[1.02; 3.54]	2.99	[1.02; 8.72]	1.51	[0.70; 3.24]	1.98	[0.53; 7.39]	1.02	0.3088
Azithromycin:Tetracycline	0	0	1.01	[0.34; 3.02]	.	.	1.01	[0.34; 3.02]	.	.	.	.
Cefixime+Probenecid:Cefotaxime	0	0	1.05	[0.10; 11.22]	.	.	1.05	[0.10; 11.22]	.	.	.	.
Cefixime+Probenecid:Ceftriaxone	0	0	0.41	[0.06; 2.75]	.	.	0.41	[0.06; 2.75]	.	.	.	.
Cefixime+Probenecid:Ceftriaxone+Amoxicillin	1	1.00	0.46	[0.08; 2.75]	0.46	[0.08; 2.75]	.	.	.	.	.	.
Cefixime+Probenecid:Ceftriaxone+Doxycycline	0	0	0.32	[0.04; 2.50]	.	.	0.32	[0.04; 2.50]	.	.	.	.
Cefixime+Probenecid:Cefuroxime	0	0	1.02	[0.13; 7.75]	.	.	1.02	[0.13; 7.75]	.	.	.	.
Cefixime+Probenecid:Doxycycline	0	0	0.66	[0.09; 4.76]	.	.	0.66	[0.09; 4.76]	.	.	.	.
Cefixime+Probenecid:Minocycline	0	0	0.11	[0.01; 1.35]	.	.	0.11	[0.01; 1.35]	.	.	.	.
Cefixime+Probenecid:Penicillin	0	0	0.93	[0.12; 7.17]	.	.	0.93	[0.12; 7.17]	.	.	.	.
Cefixime+Probenecid:Tetracycline	0	0	0.50	[0.06; 4.39]	.	.	0.50	[0.06; 4.39]	.	.	.	.
Cefotaxime:Ceftriaxone	1	0.24	0.39	[0.10; 1.59]	0.87	[0.05; 15.28]	0.30	[0.06; 1.52]	2.87	[0.11; 77.33]	0.63	0.5300
Cefotaxime:Ceftriaxone+Amoxicillin	0	0	0.44	[0.09; 2.12]	.	.	0.44	[0.09; 2.12]	.	.	.	.
Cefotaxime:Ceftriaxone+Doxycycline	0	0	0.30	[0.07; 1.36]	.	.	0.30	[0.07; 1.36]	.	.	.	.
Cefotaxime:Cefuroxime	0	0	0.97	[0.23; 4.14]	.	.	0.97	[0.23; 4.14]	.	.	.	.
Cefotaxime:Doxycycline	0	0	0.63	[0.16; 2.48]	.	.	0.63	[0.16; 2.48]	.	.	.	.
Cefotaxime:Minocycline	0	0	0.10	[0.01; 0.74]	.	.	0.10	[0.01; 0.74]	.	.	.	.
Cefotaxime:Penicillin	2	0.81	0.89	[0.24; 3.24]	0.73	[0.17; 3.06]	2.09	[0.11; 40.53]	0.35	[0.01; 9.37]	-0.63	0.5300
Cefotaxime:Tetracycline	0	0	0.47	[0.09; 2.48]	.	.	0.47	[0.09; 2.48]	.	.	.	.
Ceftriaxone:Ceftriaxone+Amoxicillin	1	1.00	1.14	[0.57; 2.27]	1.14	[0.57; 2.27]	.	.	.	.	.	.
Ceftriaxone:Ceftriaxone+Doxycycline	0	0	0.78	[0.35; 1.73]	.	.	0.78	[0.35; 1.73]	.	.	.	.
Ceftriaxone:Cefuroxime	0	0	2.50	[1.25; 4.98]	.	.	2.50	[1.25; 4.98]	.	.	.	.
Ceftriaxone:Doxycycline	2	0.95	1.63	[1.00; 2.66]	1.64	[1.00; 2.71]	1.40	[0.14; 13.86]	1.17	[0.11; 12.27]	0.13	0.8933
Ceftriaxone:Minocycline	0	0	0.27	[0.05; 1.38]	.	.	0.27	[0.05; 1.38]	.	.	.	.
Ceftriaxone:Penicillin	1	0.06	2.29	[1.11; 4.72]	4.35	[0.20; 93.86]	2.20	[1.05; 4.64]	1.97	[0.08; 46.57]	0.42	0.6730
Ceftriaxone:Tetracycline	0	0	1.22	[0.42; 3.50]	.	.	1.22	[0.42; 3.50]	.	.	.	.
Ceftriaxone+Amoxicillin:Ceftriaxone+Doxycycline	0	0	0.68	[0.24; 1.95]	.	.	0.68	[0.24; 1.95]	.	.	.	.
Ceftriaxone+Amoxicillin:Cefuroxime	0	0	2.20	[0.83; 5.81]	.	.	2.20	[0.83; 5.81]	.	.	.	.
Ceftriaxone+Amoxicillin:Doxycycline	0	0	1.43	[0.62; 3.33]	.	.	1.43	[0.62; 3.33]	.	.	.	.
Ceftriaxone+Amoxicillin:Minocycline	0	0	0.24	[0.04; 1.39]	.	.	0.24	[0.04; 1.39]	.	.	.	.
Ceftriaxone+Amoxicillin:Penicillin	0	0	2.01	[0.74; 5.45]	.	.	2.01	[0.74; 5.45]	.	.	.	.
Ceftriaxone+Amoxicillin:Tetracycline	0	0	1.07	[0.30; 3.77]	.	.	1.07	[0.30; 3.77]	.	.	.	.
Ceftriaxone+Doxycycline:Cefuroxime	0	0	3.23	[1.45; 7.17]	.	.	3.23	[1.45; 7.17]	.	.	.	.
Ceftriaxone+Doxycycline:Doxycycline	1	1.00	2.10	[1.12; 3.96]	2.10	[1.12; 3.96]	.	.	.	.	.	.
Ceftriaxone+Doxycycline:Minocycline	0	0	0.35	[0.06; 1.89]	.	.	0.35	[0.06; 1.89]	.	.	.	.
Ceftriaxone+Doxycycline:Penicillin	0	0	2.95	[1.27; 6.88]	.	.	2.95	[1.27; 6.88]	.	.	.	.
Ceftriaxone+Doxycycline:Tetracycline	0	0	1.57	[0.51; 4.87]	.	.	1.57	[0.51; 4.87]	.	.	.	.
Cefuroxime:Doxycycline	2	1.00	0.65	[0.40; 1.06]	0.65	[0.40; 1.06]	.	.	.	.	.	.
Cefuroxime:Minocycline	0	0	0.11	[0.02; 0.56]	.	.	0.11	[0.02; 0.56]	.	.	.	.
Cefuroxime:Penicillin	0	0	0.91	[0.44; 1.92]	.	.	0.91	[0.44; 1.92]	.	.	.	.
Cefuroxime:Tetracycline	0	0	0.49	[0.17; 1.40]	.	.	0.49	[0.17; 1.40]	.	.	.	.
Doxycycline:Minocycline	0	0	0.17	[0.03; 0.79]	.	.	0.17	[0.03; 0.79]	.	.	.	.
Doxycycline:Penicillin	3	0.79	1.40	[0.80; 2.46]	1.13	[0.60; 2.12]	3.22	[0.94; 11.06]	0.35	[0.09; 1.41]	-1.48	0.1395
Doxycycline:Tetracycline	1	1.00	0.75	[0.29; 1.91]	0.75	[0.29; 1.91]	.	.	.	.	.	.
Minocycline:Penicillin	1	1.00	8.50	[1.96; 36.79]	8.50	[1.96; 36.79]	.	.	.	.	.	.
Minocycline:Tetracycline	0	0	4.52	[0.73; 28.12]	.	.	4.52	[0.73; 28.12]	.	.	.	.
Penicillin:Tetracycline	0	0	0.53	[0.18; 1.59]	.	.	0.53	[0.18; 1.59]	.	.	.	.

Legend:
comparison - Treatment comparison
k - Number of studies providing direct evidence
prop - Direct evidence proportion
nma - Estimated treatment effect (OR) in network meta-analysis
direct - Estimated treatment effect (OR) derived from direct evidence
indir. - Estimated treatment effect (OR) derived from indirect evidence
RoR - Ratio of Ratios (direct versus indirect)
z - z-value of test for disagreement (direct versus indirect)
p-value - p-value of test for disagreement (direct versus indirect)
>

FIG S3 Pairwise comparison and inconsistency test of safety for antibiotics treating LD

Random effects model:

comparison	k	prop	nma	95%-CI	direct	95%-CI	indir.	95%-CI	RoR	95%-CI	z	p-value
Amoxicillin(1.5g):Amoxicillin(1.5g)+Probenecid(1.5g)	0	0	0.61	[0.14; 2.60]	.	.	0.61	[0.14; 2.60]	.			
Amoxicillin(1.5g):Azithromycin(0.25g)	0	0	2.02	[0.48; 8.45]	.	.	2.02	[0.48; 8.45]	.			
Amoxicillin(1.5g):Azithromycin(0.5g)	1	1.00	0.59	[0.34; 1.02]	0.59	[0.34; 1.02]			.			
Amoxicillin(1.5g):Cefotaxime(6g)	0	0	0.98	[0.05; 19.55]	.	.	0.98	[0.05; 19.55]	.			
Amoxicillin(1.5g):Ceftriaxone(1g)	0	0	0.43	[0.02; 12.17]	.	.	0.43	[0.02; 12.17]	.			
Amoxicillin(1.5g):Ceftriaxone(2g)	0	0	0.85	[0.36; 1.99]	.	.	0.85	[0.36; 1.99]	.			
Amoxicillin(1.5g):Ceftriaxone(2g)+Amoxicillin(2g)	0	0	0.97	[0.32; 2.89]	.	.	0.97	[0.32; 2.89]	.			
Amoxicillin(1.5g):Doxycycline(0.2g)	1	1.00	1.39	[0.70; 2.78]	1.39	[0.70; 2.78]			.			
Amoxicillin(1.5g):Doxycycline(0.2g)	0	0	0.40	[0.12; 1.34]	.	.	0.40	[0.12; 1.34]	.			
Amoxicillin(1.5g):Penicillin(12g)	0	0	1.38	[0.24; 7.90]	.	.	1.38	[0.24; 7.90]	.			
Amoxicillin(1.5g):Penicillin(1megaunit)	0	0	6.52	[0.65; 65.73]	.	.	6.52	[0.65; 65.73]	.			
Amoxicillin(1.5g):Penicillin(20megaunits)	0	0	0.71	[0.03; 19.72]	.	.	0.71	[0.03; 19.72]	.			
Amoxicillin(1.5g):Penicillin(3.9g)	1	1.00	1.38	[0.67; 2.84]	1.38	[0.67; 2.84]			.			
Amoxicillin(1.5g):Penicillin(3megaunits)	0	0	1.88	[0.51; 6.97]	.	.	1.88	[0.51; 6.97]	.			
Amoxicillin(1.5g)+Probenecid(1.5g):Azithromycin(0.25g)	1	0.76	3.33	[0.86; 12.90]	3.15	[0.67; 14.86]	3.99	[0.25; 63.42]	0.79	[0.03; 18.81]	-0.15	0.8838
Amoxicillin(1.5g)+Probenecid(1.5g):Azithromycin(0.5g)	0	0	0.97	[0.20; 4.62]	.	.	0.97	[0.20; 4.62]	.			
Amoxicillin(1.5g)+Probenecid(1.5g):Cefotaxime(6g)	0	0	1.62	[0.07; 39.07]	.	.	1.62	[0.07; 39.07]	.			
Amoxicillin(1.5g)+Probenecid(1.5g):Ceftriaxone(1g)	0	0	0.71	[0.02; 27.25]	.	.	0.71	[0.02; 27.25]	.			
Amoxicillin(1.5g)+Probenecid(1.5g):Ceftriaxone(2g)	0	0	1.40	[0.35; 5.57]	.	.	1.40	[0.35; 5.57]	.			
Amoxicillin(1.5g)+Probenecid(1.5g):Ceftriaxone(2g)+Amoxicillin(2g)	0	0	1.60	[0.34; 7.46]	.	.	1.60	[0.34; 7.46]	.			
Amoxicillin(1.5g)+Probenecid(1.5g):Doxycycline(0.2g)	2	0.84	2.30	[0.64; 8.33]	2.95	[0.72; 12.02]	0.63	[0.03; 15.58]	4.66	[0.14; 153.75]	0.86	0.3883
Amoxicillin(1.5g)+Probenecid(1.5g):Doxycycline(0.2g)	0	0	0.66	[0.10; 4.37]	.	.	0.66	[0.10; 4.37]	.			
Amoxicillin(1.5g)+Probenecid(1.5g):Penicillin(12g)	0	0	2.27	[0.29; 17.78]	.	.	2.27	[0.29; 17.78]	.			
Amoxicillin(1.5g)+Probenecid(1.5g):Penicillin(1megaunit)	0	0	10.77	[0.92; 126.70]	.	.	10.77	[0.92; 126.70]	.			
Amoxicillin(1.5g)+Probenecid(1.5g):Penicillin(20megaunits)	0	0	1.18	[0.04; 38.73]	.	.	1.18	[0.04; 38.73]	.			
Amoxicillin(1.5g)+Probenecid(1.5g):Penicillin(3.9g)	0	0	2.27	[0.52; 9.91]	.	.	2.27	[0.52; 9.91]	.			
Amoxicillin(1.5g)+Probenecid(1.5g):Penicillin(3megaunits)	0	0	3.10	[0.44; 22.03]	.	.	3.10	[0.44; 22.03]	.			
Azithromycin(0.25g):Azithromycin(0.5g)	0	0	0.29	[0.06; 1.35]	.	.	0.29	[0.06; 1.35]	.			
Azithromycin(0.25g):Cefotaxime(6g)	0	0	0.49	[0.02; 11.57]	.	.	0.49	[0.02; 11.57]	.			
Azithromycin(0.25g):Ceftriaxone(1g)	0	0	0.21	[0.01; 8.08]	.	.	0.21	[0.01; 8.08]	.			
Azithromycin(0.25g):Ceftriaxone(2g)	0	0	0.42	[0.11; 1.62]	.	.	0.42	[0.11; 1.62]	.			
Azithromycin(0.25g):Ceftriaxone(2g)+Amoxicillin(2g)	0	0	0.48	[0.11; 2.18]	.	.	0.48	[0.11; 2.18]	.			
Azithromycin(0.25g):Doxycycline(0.2g)	2	0.93	0.69	[0.20; 2.42]	0.89	[0.24; 3.27]	0.02	[0.00; 2.49]	45.66	[0.30; 6902.90]	1.49	0.1356
Azithromycin(0.25g):Doxycycline(0.2g)	0	0	0.20	[0.03; 1.28]	.	.	0.20	[0.03; 1.28]	.			
Azithromycin(0.25g):Penicillin(12g)	0	0	0.68	[0.09; 5.23]	.	.	0.68	[0.09; 5.23]	.			
Azithromycin(0.25g):Penicillin(1megaunit)	1	0.88	3.23	[0.31; 33.32]	2.22	[0.19; 26.63]	54.17	[0.06; 49521.90]	0.04	[0.00; 58.12]	-0.86	0.3883
Azithromycin(0.25g):Penicillin(20megaunits)	0	0	0.35	[0.01; 11.48]	.	.	0.35	[0.01; 11.48]	.			
Azithromycin(0.25g):Penicillin(3.9g)	0	0	0.68	[0.16; 2.89]	.	.	0.68	[0.16; 2.89]	.			
Azithromycin(0.25g):Penicillin(3megaunits)	0	0	0.93	[0.13; 6.48]	.	.	0.93	[0.13; 6.48]	.			
Azithromycin(0.5g):Cefotaxime(6g)	0	0	1.67	[0.08; 35.09]	.	.	1.67	[0.08; 35.09]	.			
Azithromycin(0.5g):Ceftriaxone(1g)	0	0	0.74	[0.03; 19.80]	.	.	0.74	[0.03; 19.80]	.			
Azithromycin(0.5g):Ceftriaxone(2g)	0	0	1.45	[0.52; 4.01]	.	.	1.45	[0.52; 4.01]	.			
Azithromycin(0.5g):Ceftriaxone(2g)+Amoxicillin(2g)	0	0	1.65	[0.48; 5.63]	.	.	1.65	[0.48; 5.63]	.			
Azithromycin(0.5g):Doxycycline(0.2g)	0	0	2.38	[0.98; 5.77]	.	.	2.38	[0.98; 5.77]	.			
Azithromycin(0.5g):Doxycycline(0.2g)	2	1.00	0.68	[0.23; 1.99]	0.68	[0.23; 1.99]			.			
Azithromycin(0.5g):Penicillin(12g)	0	0	2.35	[0.38; 14.69]	.	.	2.35	[0.38; 14.69]	.			
Azithromycin(0.5g):Penicillin(1megaunit)	0	0	11.12	[1.03; 119.79]	.	.	11.12	[1.03; 119.79]	.			
Azithromycin(0.5g):Penicillin(20megaunits)	0	0	1.22	[0.04; 35.24]	.	.	1.22	[0.04; 35.24]	.			
Azithromycin(0.5g):Penicillin(3.9g)	0	0	2.34	[0.94; 5.85]	.	.	2.34	[0.94; 5.85]	.			
Azithromycin(0.5g):Penicillin(3megaunits)	1	1.00	3.20	[0.98; 10.49]	3.20	[0.98; 10.49]			.			
Cefotaxime(6g):Ceftriaxone(1g)	0	0	0.44	[0.00; 39.02]	.	.	0.44	[0.00; 39.02]	.			
Cefotaxime(6g):Ceftriaxone(2g)	1	1.00	0.87	[0.05; 15.28]	0.87	[0.05; 15.28]			.			
Cefotaxime(6g):Ceftriaxone(2g)+Amoxicillin(2g)	0	0	0.99	[0.05; 18.88]	.	.	0.99	[0.05; 18.88]	.			
Cefotaxime(6g):Doxycycline(0.2g)	0	0	1.42	[0.08; 26.21]	.	.	1.42	[0.08; 26.21]	.			
Cefotaxime(6g):Doxycycline(0.2g)	0	0	0.41	[0.02; 10.26]	.	.	0.41	[0.02; 10.26]	.			
Cefotaxime(6g):Penicillin(12g)	0	0	1.41	[0.05; 39.11]	.	.	1.41	[0.05; 39.11]	.			
Cefotaxime(6g):Penicillin(1megaunit)	0	0	6.66	[0.17; 257.03]	.	.	6.66	[0.17; 257.03]	.			
Cefotaxime(6g):Penicillin(20megaunits)	2	1.00	0.73	[0.17; 3.06]	0.73	[0.17; 3.06]			.			
Cefotaxime(6g):Penicillin(3.9g)	0	0	1.40	[0.07; 28.20]	.	.	1.40	[0.07; 28.20]	.			
Cefotaxime(6g):Penicillin(3megaunits)	0	0	1.92	[0.07; 50.28]	.	.	1.92	[0.07; 50.28]	.			
Ceftriaxone(1g):Ceftriaxone(2g)	0	0	1.97	[0.06; 61.80]	.	.	1.97	[0.06; 61.80]	.			
Ceftriaxone(1g):Ceftriaxone(2g)+Amoxicillin(2g)	0	0	2.24	[0.07; 75.36]	.	.	2.24	[0.07; 75.36]	.			
Ceftriaxone(1g):Doxycycline(0.2g)	0	0	3.23	[0.11; 97.87]	.	.	3.23	[0.11; 97.87]	.			
Ceftriaxone(1g):Doxycycline(0.2g)	0	0	0.92	[0.03; 29.47]	.	.	0.92	[0.03; 29.47]	.			
Ceftriaxone(1g):Penicillin(12g)	0	0	3.19	[0.07; 138.37]	.	.	3.19	[0.07; 138.37]	.			
Ceftriaxone(1g):Penicillin(1megaunit)	0	0	15.13	[0.26; 877.59]	.	.	15.13	[0.26; 877.59]	.			
Ceftriaxone(1g):Penicillin(20megaunits)	0	0	1.65	[0.01; 183.49]	.	.	1.65	[0.01; 183.49]	.			
Ceftriaxone(1g):Penicillin(3.9g)	0	0	3.19	[0.10; 97.19]	.	.	3.19	[0.10; 97.19]	.			
Ceftriaxone(1g):Penicillin(3megaunits)	1	1.00	4.35	[0.20; 93.86]	4.35	[0.20; 93.86]			.			
Ceftriaxone(2g):Ceftriaxone(2g)+Amoxicillin(2g)	1	1.00	1.14	[0.57; 2.27]	1.14	[0.57; 2.27]			.			
Ceftriaxone(2g):Doxycycline(0.2g)	2	1.00	1.64	[1.00; 2.71]	1.64	[1.00; 2.71]			.			
Ceftriaxone(2g):Doxycycline(0.2g)	0	0	0.47	[0.11; 2.06]	.	.	0.47	[0.11; 2.06]	.			
Ceftriaxone(2g):Penicillin(12g)	0	0	1.62	[0.30; 8.71]	.	.	1.62	[0.30; 8.71]	.			
Ceftriaxone(2g):Penicillin(1megaunit)	0	0	7.68	[0.80; 73.70]	.	.	7.68	[0.80; 73.70]	.			
Ceftriaxone(2g):Penicillin(20megaunits)	0	0	0.84	[0.03; 20.79]	.	.	0.84	[0.03; 20.79]	.			
Ceftriaxone(2g):Penicillin(3.9g)	0	0	1.62	[0.67; 3.89]	.	.	1.62	[0.67; 3.89]	.			
Ceftriaxone(2g):Penicillin(3megaunits)	0	0	2.21	[0.46; 10.55]	.	.	2.21	[0.46; 10.55]	.			
Ceftriaxone(2g)+Amoxicillin(2g):Doxycycline(0.2g)	0	0	1.44	[0.62; 3.37]	.	.	1.44	[0.62; 3.37]	.			
Ceftriaxone(2g)+Amoxicillin(2g):Doxycycline(0.2g)	0	0	0.41	[0.08; 2.10]	.	.	0.41	[0.08; 2.10]	.			
Ceftriaxone(2g)+Amoxicillin(2g):Penicillin(12g)	0	0	1.42	[0.23; 8.75]	.	.	1.42	[0.23; 8.75]	.			
Ceftriaxone(2g)+Amoxicillin(2g):Penicillin(1megaunit)	0	0	6.74	[0.63; 71.66]	.	.	6.74	[0.63; 71.66]	.			
Ceftriaxone(2g)+Amoxicillin(2g):Penicillin(20megaunits)	0	0	0.74	[0.03; 19.63]	.	.	0.74	[0.03; 19.63]	.			
Ceftriaxone(2g)+Amoxicillin(2g):Penicillin(3.9g)	0	0	1.42	[0.47; 4.33]	.	.	1.42	[0.47; 4.33]	.			
Ceftriaxone(2g)+Amoxicillin(2g):Penicillin(3megaunits)	0	0	1.94	[0.35; 10.70]	.	.	1.94	[0.35; 10.70]	.			
Doxycycline(0.2g):Doxycycline(0.2g)	0	0	0.29	[0.07; 1.15]	.	.	0.29	[0.07; 1.15]	.			
Doxycycline(0.2g):Penicillin(12g)	1	1.00	0.99	[0.20; 4.92]	0.99	[0.20; 4.92]			.			
Doxycycline(0.2g):Penicillin(1megaunit)	1	0.97	4.68	[0.52; 42.43]	5.56	[0.59; 52.16]	0.02	[0.00; 5997.61]	283.03	[0.00; 105310560.69]	0.86	0.3883
Doxycycline(0.2g):Penicillin(20megaunits)	0	0	0.51	[0.02; 13.16]	.	.	0.51	[0.02; 13.16]	.			
Doxycycline(0.2g):Penicillin(3.9g)	1	1.00	0.99	[0.48; 2.02]	0.99	[0.48; 2.02]			.			
Doxycycline(0.2g):Penicillin(3megaunits)	0	0	1.35	[0.31; 5.92]	.	.	1.35	[0.31; 5.92]	.			
Doxycycline(0.2g) :Penicillin(12g)	0	0	3.46	[0.41; 28.95]	.	.	3.46	[0.41; 28.95]	.			
Doxycycline(0.2g) :Penicillin(1megaunit)	0	0	16.38	[1.21; 222.31]	.	.	16.38	[1.21; 222.31]	.			
Doxycycline(0.2g) :Penicillin(20megaunits)	0	0	1.79	[0.05; 61.34]	.	.	1.79	[0.05; 61.34]	.			
Doxycycline(0.2g) :Penicillin(3.9g)	0	0	3.45	[0.84; 14.14]	.	.	3.45	[0.84; 14.14]	.			
Doxycycline(0.2g) :Penicillin(3megaunits)	0	0	4.71	[0.95; 23.35]	.	.	4.71	[0.95; 23.35]	.			
Penicillin(12g):Penicillin(1megaunit)	0	0	4.74	[0.31; 72.41]	.	.	4.74	[0.31; 72.41]	.			
Penicillin(12g):Penicillin(20megaunits)	0	0	0.52	[0.01; 19.38]	.	.	0.52	[0.01; 19.38]	.			
Penicillin(12g):Penicillin(3.9g)	0	0	1.00	[0.17; 5.79]	.	.	1.00	[0.17; 5.79]	.			
Penicillin(12g):Penicillin(3megaunits)	0	0	1.36	[0.15; 12.10]	.	.	1.36	[0.15; 12.10]	.			
Penicillin(1megaunit):Penicillin(20megaunits)	0	0	0.11	[0.00; 5.54]	.	.	0.11	[0.00; 5.54]	.			
Penicillin(1megaunit):Penicillin(3.9g)	0	0	0.21	[0.02; 2.14]	.	.	0.21	[0.02; 2.14]	.			
Penicillin(1megaunit):Penicillin(3megaunits)	0	0	0.29	[0.02; 4.10]	.	.	0.29	[0.02; 4.10]	.			
Penicillin(20megaunits):Penicillin(3.9g)	0	0	1.93	[0.07; 53.60]	.	.	1.93	[0.07; 53.60]	.			
Penicillin(20megaunits):Penicillin(3megaunits)	0	0	2.63	[0.07; 93.29]	.	.	2.63	[0.07; 93.29]	.			
Penicillin(3.9g):Penicillin(3megaunits)	0	0	1.36	[0.31; 6.11]	.	.	1.36	[0.31; 6.11]	.			

Legend:

- comparison - Treatment comparison
- k - Number of studies providing direct evidence
- prop - Direct evidence proportion
- nma - Estimated treatment effect (OR) in network meta-analysis
- direct - Estimated treatment effect (OR) derived from direct evidence
- indir. - Estimated treatment effect (OR) derived from indirect evidence
- RoR - Ratio of Ratios (direct versus indirect)
- z - z-value of test for disagreement (direct versus indirect)
- p-value - p-value of test for disagreement (direct versus indirect)

>

FIG S4 Pairwise comparison and inconsistency test of safety for different daily dosages of antibiotics treating LD

Random effects model:

comparison	k	prop	nma	95%-CI	direct	95%-CI	indir.	95%-CI	RoR	95%-CI	z	p-value
Amoxicillin:Azithromycin	1	1.00	0.20	[0.01; 4.24]	0.20	[0.01; 4.24]	.	.	.	.	.	.
Amoxicillin:Ceftriaxone	0	0	6.71	[0.02; 2044.46]	.	.	6.71	[0.02; 2044.46]	.	.	.	.
Amoxicillin:Cefuroxime	1	1.00	7.22	[0.37; 139.15]	7.22	[0.37; 139.15]	.	.	.	.	.	.
Amoxicillin:Clarithromycin	1	1.00	0.21	[0.01; 4.44]	0.21	[0.01; 4.44]	.	.	.	.	.	.
Amoxicillin:Penicillin	0	0	7.38	[0.05; 1020.17]	.	.	7.38	[0.05; 1020.17]	.	.	.	.
Azithromycin:Ceftriaxone	0	0	33.57	[0.05; 21944.40]	.	.	33.57	[0.05; 21944.40]	.	.	.	.
Azithromycin:Cefuroxime	0	0	36.11	[0.51; 2534.78]	.	.	36.11	[0.51; 2534.78]	.	.	.	.
Azithromycin:Clarithromycin	0	0	1.04	[0.01; 78.42]	.	.	1.04	[0.01; 78.42]	.	.	.	.
Azithromycin:Penicillin	0	0	36.92	[0.11; 12163.81]	.	.	36.92	[0.11; 12163.81]	.	.	.	.
Ceftriaxone:Cefuroxime	0	0	1.08	[0.01; 143.63]	.	.	1.08	[0.01; 143.63]	.	.	.	.
Ceftriaxone:Clarithromycin	0	0	0.03	[0.00; 20.32]	.	.	0.03	[0.00; 20.32]	.	.	.	.
Ceftriaxone:Penicillin	1	1.00	1.10	[0.06; 20.01]	1.10	[0.06; 20.01]	.	.	.	.	.	.
Cefuroxime:Clarithromycin	0	0	0.03	[0.00; 2.03]	.	.	0.03	[0.00; 2.03]	.	.	.	.
Cefuroxime:Penicillin	1	1.00	1.02	[0.02; 52.66]	1.02	[0.02; 52.66]	.	.	.	.	.	.
Clarithromycin:Penicillin	0	0	35.52	[0.11; 11748.42]	.	.	35.52	[0.11; 11748.42]	.	.	.	.

Legend:
comparison - Treatment comparison
k - Number of studies providing direct evidence
prop - Direct evidence proportion
nma - Estimated treatment effect (OR) in network meta-analysis
direct - Estimated treatment effect (OR) derived from direct evidence
indir. - Estimated treatment effect (OR) derived from indirect evidence
RoR - Ratio of Ratios (direct versus indirect)
z - z-value of test for disagreement (direct versus indirect)
p-value - p-value of test for disagreement (direct versus indirect)

FIG S5 Pairwise comparison and inconsistency test of efficacy for antibiotics treating children's LD

Random effects model:

comparison	k	prop	nma	95%-CI	direct	95%-CI	indir.	95%-CI	RoR	95%-CI	z	p-value
Cefotaxime:Ceftriaxone	1	0.56	0.60	[0.17; 2.10]	0.75	[0.14; 4.01]	0.45	[0.07; 2.97]	1.68	[0.13; 21.19]	0.40	0.6869
Cefotaxime:Doxycycline	0	0	3.86	[0.84; 17.78]	.	.	3.86	[0.84; 17.78]	.	.	.	.
Cefotaxime:Penicillin	2	0.87	3.61	[1.52; 8.57]	3.37	[1.33; 8.52]	5.67	[0.54; 59.76]	0.59	[0.05; 7.48]	-0.40	0.6869
Ceftriaxone:Doxycycline	0	0	6.46	[1.09; 38.21]	.	.	6.46	[1.09; 38.21]	.	.	.	.
Ceftriaxone:Penicillin	2	0.57	6.05	[1.73; 21.17]	7.55	[1.44; 39.56]	4.49	[0.66; 30.50]	1.68	[0.13; 21.19]	0.40	0.6869
Doxycycline:Penicillin	1	1.00	0.94	[0.27; 3.30]	0.94	[0.27; 3.30]	.	.	.	.	.	.

Legend:
comparison - Treatment comparison
k - Number of studies providing direct evidence
prop - Direct evidence proportion
nma - Estimated treatment effect (OR) in network meta-analysis
direct - Estimated treatment effect (OR) derived from direct evidence
indir. - Estimated treatment effect (OR) derived from indirect evidence
RoR - Ratio of Ratios (direct versus indirect)
z - z-value of test for disagreement (direct versus indirect)
p-value - p-value of test for disagreement (direct versus indirect)

FIG S6 Pairwise comparison and inconsistency test of efficacy for injectable antibiotics treating LD

Random effects model:

comparison	k	prop	nma	95%-CI	direct	95%-CI	indir.	95%-CI	RoR	95%-CI	z	p-value
Amoxicillin:Amoxicillin+probenecid	0	0	1.99	[0.28; 14.30]	.	.	1.99	[0.28; 14.30]	.	.	.	.
Amoxicillin:Azithromycin	1	0.89	2.17	[0.71; 6.59]	2.30	[0.71; 7.47]	1.35	[0.05; 38.48]	1.70	[0.05; 59.25]	0.29	0.7692
Amoxicillin:Cefuroxime	0	0	5.05	[0.85; 30.07]	.	.	5.05	[0.85; 30.07]	.	.	.	.
Amoxicillin:Doxycycline	1	0.12	4.38	[1.06; 18.11]	0.93	[0.02; 52.84]	5.45	[1.20; 24.82]	0.17	[0.00; 12.77]	-0.80	0.4215
Amoxicillin:Erythromycin	0	0	2.14	[0.32; 14.35]	.	.	2.14	[0.32; 14.35]	.	.	.	.
Amoxicillin:Penicillin	1	0.18	3.57	[0.85; 14.93]	3.50	[0.12; 99.87]	3.59	[0.74; 17.46]	0.97	[0.02; 39.69]	-0.01	0.9890
Amoxicillin:Tetracycline	0	0	1.30	[0.22; 7.54]	.	.	1.30	[0.22; 7.54]	.	.	.	.
Amoxicillin+probenecid:Azithromycin	1	0.69	1.09	[0.21; 5.72]	1.23	[0.17; 9.00]	0.83	[0.04; 16.49]	1.48	[0.04; 53.59]	0.21	0.8305
Amoxicillin+probenecid:Cefuroxime	0	0	2.54	[0.38; 16.93]	.	.	2.54	[0.38; 16.93]	.	.	.	.
Amoxicillin+probenecid:Doxycycline	2	0.91	2.20	[0.46; 10.47]	2.17	[0.42; 11.10]	2.62	[0.01; 465.20]	0.83	[0.00; 189.26]	-0.07	0.9456
Amoxicillin+probenecid:Erythromycin	0	0	1.08	[0.12; 9.41]	.	.	1.08	[0.12; 9.41]	.	.	.	.
Amoxicillin+probenecid:Penicillin	0	0	1.80	[0.30; 10.80]	.	.	1.80	[0.30; 10.80]	.	.	.	.
Amoxicillin+probenecid:Tetracycline	0	0	0.65	[0.09; 4.92]	.	.	0.65	[0.09; 4.92]	.	.	.	.
Azithromycin:Cefuroxime	0	0	2.33	[0.55; 9.83]	.	.	2.33	[0.55; 9.83]	.	.	.	.
Azithromycin:Doxycycline	3	0.82	2.02	[0.78; 5.23]	2.66	[0.93; 7.62]	0.59	[0.06; 5.49]	4.47	[0.38; 52.38]	1.19	0.2329
Azithromycin:Erythromycin	0	0	0.99	[0.20; 4.91]	.	.	0.99	[0.20; 4.91]	.	.	.	.
Azithromycin:Penicillin	2	0.73	1.65	[0.60; 4.50]	1.48	[0.46; 4.77]	2.23	[0.32; 15.59]	0.66	[0.07; 6.44]	-0.35	0.7232
Azithromycin:Tetracycline	0	0	0.60	[0.14; 2.50]	.	.	0.60	[0.14; 2.50]	.	.	.	.
Cefuroxime:Doxycycline	2	1.00	0.87	[0.29; 2.55]	0.87	[0.29; 2.55]	.	.	.	.	.	.
Cefuroxime:Erythromycin	0	0	0.42	[0.06; 2.92]	.	.	0.42	[0.06; 2.92]	.	.	.	.
Cefuroxime:Penicillin	0	0	0.71	[0.16; 3.21]	.	.	0.71	[0.16; 3.21]	.	.	.	.
Cefuroxime:Tetracycline	0	0	0.26	[0.04; 1.48]	.	.	0.26	[0.04; 1.48]	.	.	.	.
Doxycycline:Erythromycin	0	0	0.49	[0.10; 2.42]	.	.	0.49	[0.10; 2.42]	.	.	.	.
Doxycycline:Penicillin	2	0.55	0.82	[0.28; 2.35]	2.36	[0.57; 9.80]	0.22	[0.04; 1.07]	10.75	[1.28; 90.59]	2.18	0.0289
Doxycycline:Tetracycline	1	0.37	0.30	[0.07; 1.18]	0.10	[0.01; 0.97]	0.56	[0.10; 3.15]	0.18	[0.01; 3.14]	-1.18	0.2394
Erythromycin:Penicillin	1	0.95	1.67	[0.45; 6.16]	1.40	[0.37; 5.34]	57.06	[0.14; 23720.11]	0.02	[0.00; 11.81]	-1.18	0.2394
Erythromycin:Tetracycline	1	0.95	0.61	[0.16; 2.24]	0.72	[0.19; 2.75]	0.02	[0.00; 7.35]	40.72	[0.08; 19572.41]	1.18	0.2394
Penicillin:Tetracycline	1	0.80	0.36	[0.11; 1.15]	0.52	[0.14; 1.87]	0.09	[0.01; 1.19]	5.58	[0.32; 97.84]	1.18	0.2394

Legend:
comparison - Treatment comparison
k - Number of studies providing direct evidence
prop - Direct evidence proportion
nma - Estimated treatment effect (OR) in network meta-analysis
direct - Estimated treatment effect (OR) derived from direct evidence
indir. - Estimated treatment effect (OR) derived from indirect evidence
RoR - Ratio of Ratios (direct versus indirect)
z - z-value of test for disagreement (direct versus indirect)
p-value - p-value of test for disagreement (direct versus indirect)
>

FIG S7 Pairwise comparison and inconsistency test of efficacy for oral antibiotics treating LD

Random effects model:

comparison	k	prop	nma	95%-CI	direct	95%-CI	indir.	95%-CI	RoR	95%-CI	z	p-value
Amoxicillin(1.5g):Amoxicillin(1.5g)+Probenecid(1.5g)	0	0	3.28	[0.39; 27.76]	.	.	3.28	[0.39; 27.76]	.	.	.	.
Amoxicillin(1.5g):Azithromycin(0.25g)	0	0	3.71	[0.52; 26.26]	.	.	3.71	[0.52; 26.26]	.	.	.	.
Amoxicillin(1.5g):Azithromycin(0.5g)	1	0.97	2.14	[1.05; 4.38]	2.30	[1.11; 4.75]	0.19	[0.00; 13.46]	12.10	[0.16; 911.43]	1.13	0.2581
Amoxicillin(1.5g):Doxycycline(0.2g)	1	0.17	7.32	[1.44; 37.28]	0.93	[0.02; 47.42]	11.22	[1.88; 67.01]	0.08	[0.00; 6.22]	-1.13	0.2581
Amoxicillin(1.5g):Penicillin(1megaunit)	0	0	15.64	[2.06; 118.55]	.	.	15.64	[2.06; 118.55]	.	.	.	.
Amoxicillin(1.5g):Penicillin(3.9g)	1	0.69	9.83	[0.68; 142.86]	3.50	[0.14; 87.57]	98.71	[0.80; 12134.89]	0.04	[0.00; 11.58]	-1.13	0.2581
Amoxicillin(1.5g):Penicillin(3megaunits)	0	0	1.37	[0.32; 5.86]	.	.	1.37	[0.32; 5.86]	.	.	.	.
Amoxicillin(1.5g)+Probenecid(1.5g):Azithromycin(0.25g)	1	0.77	1.13	[0.24; 5.33]	1.23	[0.21; 7.15]	0.86	[0.03; 22.05]	1.44	[0.04; 57.87]	0.19	0.8472
Amoxicillin(1.5g)+Probenecid(1.5g):Azithromycin(0.5g)	0	0	0.65	[0.08; 5.09]	.	.	0.65	[0.08; 5.09]	.	.	.	.
Amoxicillin(1.5g)+Probenecid(1.5g):Doxycycline(0.2g)	2	0.95	2.24	[0.56; 8.94]	2.23	[0.54; 9.23]	2.41	[0.01; 1087.63]	0.92	[0.00; 490.68]	-0.02	0.9802
Amoxicillin(1.5g)+Probenecid(1.5g):Penicillin(1megaunit)	0	0	4.78	[0.82; 27.90]	.	.	4.78	[0.82; 27.90]	.	.	.	.
Amoxicillin(1.5g)+Probenecid(1.5g):Penicillin(3.9g)	0	0	3.00	[0.15; 61.10]	.	.	3.00	[0.15; 61.10]	.	.	.	.
Amoxicillin(1.5g)+Probenecid(1.5g):Penicillin(3megaunits)	0	0	0.42	[0.04; 4.65]	.	.	0.42	[0.04; 4.65]	.	.	.	.
Azithromycin(0.25g):Azithromycin(0.5g)	0	0	0.58	[0.09; 3.72]	.	.	0.58	[0.09; 3.72]	.	.	.	.
Azithromycin(0.25g):Doxycycline(0.2g)	2	0.99	1.97	[0.67; 5.84]	2.01	[0.68; 5.98]	0.16	[0.00; 39706.49]	12.55	[0.00; 3260978.07]	0.40	0.6909
Azithromycin(0.25g):Penicillin(1megaunit)	1	0.79	4.21	[1.11; 15.95]	4.25	[0.95; 19.07]	4.08	[0.23; 72.75]	1.04	[0.04; 26.84]	0.02	0.9802
Azithromycin(0.25g):Penicillin(3.9g)	0	0	2.65	[0.15; 47.55]	.	.	2.65	[0.15; 47.55]	.	.	.	.
Azithromycin(0.25g):Penicillin(3megaunits)	0	0	0.37	[0.04; 3.50]	.	.	0.37	[0.04; 3.50]	.	.	.	.
Azithromycin(0.5g):Doxycycline(0.2g)	1	0.86	3.42	[0.75; 15.50]	4.88	[0.95; 25.00]	0.40	[0.01; 22.03]	12.10	[0.16; 911.43]	1.13	0.2581
Azithromycin(0.5g):Penicillin(1megaunit)	0	0	7.30	[1.05; 50.49]	.	.	7.30	[1.05; 50.49]	.	.	.	.
Azithromycin(0.5g):Penicillin(3.9g)	0	0	4.58	[0.31; 67.57]	.	.	4.58	[0.31; 67.57]	.	.	.	.
Azithromycin(0.5g):Penicillin(3megaunits)	1	1.00	0.64	[0.18; 2.27]	0.64	[0.18; 2.27]	.	.	.	.	.	.
Doxycycline(0.2g):Penicillin(1megaunit)	1	0.90	2.14	[0.64; 7.13]	2.12	[0.60; 7.57]	2.24	[0.05; 104.09]	0.95	[0.02; 54.25]	-0.02	0.9802
Doxycycline(0.2g):Penicillin(3.9g)	1	0.69	1.34	[0.09; 19.50]	3.77	[0.15; 94.40]	0.13	[0.00; 16.43]	28.25	[0.09; 9249.16]	1.13	0.2581
Doxycycline(0.2g):Penicillin(3megaunits)	0	0	0.19	[0.03; 1.34]	.	.	0.19	[0.03; 1.34]	.	.	.	.
Penicillin(1megaunit):Penicillin(3.9g)	0	0	0.63	[0.03; 11.83]	.	.	0.63	[0.03; 11.83]	.	.	.	.
Penicillin(1megaunit):Penicillin(3megaunits)	0	0	0.09	[0.01; 0.88]	.	.	0.09	[0.01; 0.88]	.	.	.	.
Penicillin(3.9g):Penicillin(3megaunits)	0	0	0.14	[0.01; 2.72]	.	.	0.14	[0.01; 2.72]	.	.	.	.

Legend:
comparison - Treatment comparison
k - Number of studies providing direct evidence
prop - Direct evidence proportion
nma - Estimated treatment effect (OR) in network meta-analysis
direct - Estimated treatment effect (OR) derived from direct evidence
indir. - Estimated treatment effect (OR) derived from indirect evidence
RoR - Ratio of Ratios (direct versus indirect)
z - z-value of test for disagreement (direct versus indirect)
p-value - p-value of test for disagreement (direct versus indirect)
>

FIG S8 Pairwise comparison and inconsistency test of efficacy for different daily dosages of oral antibiotics treating LD

```
Random effects model:

                          comparison k prop  nma        95%-CI direct        95%-CI indir.        95%-CI  RoR        95%-CI     z p-value
  Amoxicillin:Amoxicillin+probenecid 0    0 0.32 [0.07;  1.37]    .              .   0.32 [0.07;  1.37]    .              .      .      .
          Amoxicillin:Azithromycin 1 0.62 0.73 [0.34;  1.58]  0.59 [0.22;  1.55]   1.05 [0.31;  3.63] 0.56 [0.12;  2.69] -0.73  0.4665
            Amoxicillin:Cefuroxime 0    0 1.30 [0.42;  4.00]    .              .   1.30 [0.42;  4.00]    .              .      .      .
           Amoxicillin:Doxycycline 1 0.63 0.88 [0.38;  2.03]  1.39 [0.49;  4.00]   0.39 [0.10;  1.56] 3.57 [0.63; 20.38]  1.43  0.1520
            Amoxicillin:Penicillin 1 0.68 1.51 [0.62;  3.65]  1.38 [0.47;  4.03]   1.82 [0.38;  8.71] 0.75 [0.11;  5.03] -0.29  0.7706
          Amoxicillin:Tetracycline 0    0 0.65 [0.15;  2.90]    .              .   0.65 [0.15;  2.90]    .              .      .      .
 Amoxicillin+probenecid:Azithromycin 1 0.61 2.32 [0.59;  9.10]  3.15 [0.55; 18.02]   1.44 [0.16; 12.89] 2.19 [0.13; 36.19]  0.55  0.5825
   Amoxicillin+probenecid:Cefuroxime 0    0 4.13 [0.90; 18.96]    .              .   4.13 [0.90; 18.96]    .              .      .      .
  Amoxicillin+probenecid:Doxycycline 2 0.76 2.78 [0.74; 10.50]  2.79 [0.61; 12.78]   2.76 [0.18; 41.79] 1.01 [0.04; 22.79]  0.01  0.9948
   Amoxicillin+probenecid:Penicillin 0    0 4.78 [1.06; 21.44]    .              .   4.78 [1.06; 21.44]    .              .      .      .
 Amoxicillin+probenecid:Tetracycline 0    0 2.07 [0.34; 12.68]    .              .   2.07 [0.34; 12.68]    .              .      .      .
           Azithromycin:Cefuroxime 0    0 1.78 [0.60;  5.25]    .              .   1.78 [0.60;  5.25]    .              .      .      .
          Azithromycin:Doxycycline 3 0.57 1.20 [0.54;  2.62]  0.81 [0.29;  2.31]   1.98 [0.60;  6.53] 0.41 [0.08;  2.00] -1.10  0.2697
           Azithromycin:Penicillin 2 0.48 2.05 [0.86;  4.90]  2.94 [0.84; 10.30]   1.47 [0.44;  4.92] 2.00 [0.35; 11.39]  0.78  0.4355
         Azithromycin:Tetracycline 0    0 0.89 [0.21;  3.84]    .              .   0.89 [0.21;  3.84]    .              .      .      .
            Cefuroxime:Doxycycline 2 1.00 0.67 [0.32;  1.42]  0.67 [0.32;  1.42]     .              .    .              .      .      .
             Cefuroxime:Penicillin 0    0 1.16 [0.37;  3.62]    .              .   1.16 [0.37;  3.62]    .              .      .      .
           Cefuroxime:Tetracycline 0    0 0.50 [0.12;  2.12]    .              .   0.50 [0.12;  2.12]    .              .      .      .
            Doxycycline:Penicillin 2 0.78 1.72 [0.73;  4.07]  1.32 [0.50;  3.51]   4.33 [0.69; 27.05] 0.31 [0.04;  2.44] -1.12  0.2630
          Doxycycline:Tetracycline 1 1.00 0.75 [0.22;  2.55]  0.75 [0.22;  2.55]     .              .    .              .      .      .
            Penicillin:Tetracycline 0    0 0.43 [0.10;  1.95]    .              .   0.43 [0.10;  1.95]    .              .      .      .

Legend:
 comparison - Treatment comparison
 k          - Number of studies providing direct evidence
 prop       - Direct evidence proportion
 nma        - Estimated treatment effect (OR) in network meta-analysis
 direct     - Estimated treatment effect (OR) derived from direct evidence
 indir.     - Estimated treatment effect (OR) derived from indirect evidence
 RoR        - Ratio of Ratios (direct versus indirect)
 z          - z-value of test for disagreement (direct versus indirect)
 p-value    - p-value of test for disagreement (direct versus indirect)
```

FIG S9 Pairwise comparison and inconsistency test of safety for oral antibiotics treating LD

```
Random effects model:

                                           comparison k prop  nma       95%-CI direct       95%-CI indir.          95%-CI   RoR
95%-CI     z p-value
     Amoxicillin(1.5g):Amoxicillin(1.5g)+Probenecid(1.5g) 0    0 0.47 [0.06;   3.73]     .             .   0.47 [0.06;
3.73]     .          .    .    .
                   Amoxicillin(1.5g):Azithromycin(0.25g) 0    0 1.42 [0.19;  10.72]     .             .   1.42 [0.19;
10.72]     .          .    .    .
                    Amoxicillin(1.5g):Azithromycin(0.5g) 1 0.73 0.81 [0.23;   2.85]  0.59 [0.13;  2.55]   1.95 [0.17;     22.29]  0.30 [0.02;
5.16] -0.83  0.4072
                     Amoxicillin(1.5g):Doxycycline(0.2g) 1 0.71 0.99 [0.27;   3.58]  1.39 [0.30;  6.41]   0.42 [0.04;      4.62]  3.33 [0.19;
57.25]  0.83  0.4072
                  Amoxicillin(1.5g):Penicillin(1megaunit) 0    0 4.43 [0.26;  76.96]     .             .   4.43 [0.26;
76.96]     .          .    .    .
                      Amoxicillin(1.5g):Penicillin(3.9g) 1 0.93 1.16 [0.26;   5.11]  1.38 [0.29;  6.42]   0.12 [0.00;     31.64] 11.60 [0.04;
3813.71]  0.83  0.4072
                 Amoxicillin(1.5g):Penicillin(3megaunits) 0    0 2.59 [0.29;  23.37]     .             .   2.59 [0.29;
23.37]     .          .    .    .
    Amoxicillin(1.5g)+Probenecid(1.5g):Azithromycin(0.25g) 1 0.74 2.99 [0.51;  17.57]  3.15 [0.40; 24.80]   2.58 [0.08;     81.68]  1.22 [0.02;
68.29]  0.10  0.9224
     Amoxicillin(1.5g)+Probenecid(1.5g):Azithromycin(0.5g) 0    0 1.71 [0.20;  14.62]     .             .   1.71 [0.20;
14.62]     .          .    .    .
      Amoxicillin(1.5g)+Probenecid(1.5g):Doxycycline(0.2g) 2 0.88 2.09 [0.41;  10.49]  2.58 [0.46; 14.44]   0.46 [0.00;     46.52]  5.62 [0.04;
779.18]  0.69  0.4925
  Amoxicillin(1.5g)+Probenecid(1.5g):Penicillin(1megaunit) 0    0 9.36 [0.52; 168.85]     .             .   9.36 [0.52;
168.85]     .          .    .    .
       Amoxicillin(1.5g)+Probenecid(1.5g):Penicillin(3.9g) 0    0 2.44 [0.27;  21.90]     .             .   2.44 [0.27;
21.90]     .          .    .    .
 Amoxicillin(1.5g)+Probenecid(1.5g):Penicillin(3megaunits) 0    0 5.47 [0.33;  90.32]     .             .   5.47 [0.33;
90.32]     .          .    .    .
                  Azithromycin(0.25g):Azithromycin(0.5g) 0    0 0.57 [0.07;   4.70]     .             .   0.57 [0.07;
4.70]     .          .    .    .
                   Azithromycin(0.25g):Doxycycline(0.2g) 2 0.93 0.70 [0.15;   3.33]  0.92 [0.18;  4.62]   0.02 [0.00;      6.60] 57.90 [0.11;
29900.28]  1.27  0.2029
              Azithromycin(0.25g):Penicillin(1megaunit) 1 0.88 3.13 [0.22;  44.62]  2.22 [0.13; 37.71]  39.15 [0.02; 85468.79]  0.06 [0.00;
205.30] -0.69  0.4925
                    Azithromycin(0.25g):Penicillin(3.9g) 0    0 0.82 [0.09;   7.05]     .             .   0.82 [0.09;
7.05]     .          .    .    .
             Azithromycin(0.25g):Penicillin(3megaunits) 0    0 1.83 [0.11;  29.32]     .             .   1.83 [0.11;
29.32]     .          .    .    .
                    Azithromycin(0.5g):Doxycycline(0.2g) 1 0.55 1.22 [0.30;   5.02]  0.71 [0.11;  4.77]   2.38 [0.29;     19.77]  0.30 [0.02;
5.16] -0.83  0.4072
                Azithromycin(0.5g):Penicillin(1megaunit) 0    0 5.48 [0.30; 100.99]     .             .   5.48 [0.30;
100.99]     .          .    .    .
                     Azithromycin(0.5g):Penicillin(3.9g) 0    0 1.43 [0.24;   8.48]     .             .   1.43 [0.24;
8.48]     .          .    .    .
              Azithromycin(0.5g):Penicillin(3megaunits) 1 1.00 3.20 [0.53;  19.46]  3.20 [0.53;
19.46]     .          .    .          .    .    .
                Doxycycline(0.2g):Penicillin(1megaunit) 1 0.95 4.49 [0.35;  57.36]  5.56 [0.40; 76.32]   0.11 [0.00;   6127.60] 51.49 [0.00;
3983198.81]  0.69  0.4925
                     Doxycycline(0.2g):Penicillin(3.9g) 1 0.93 1.17 [0.27;   5.16]  0.99 [0.21;  4.59]  11.52 [0.04;   3131.34]  0.09 [0.00;
28.63] -0.83  0.4072
               Doxycycline(0.2g):Penicillin(3megaunits) 0    0 2.62 [0.26;  25.95]     .             .   2.62 [0.26;
25.95]     .          .    .    .
                Penicillin(1megaunit):Penicillin(3.9g) 0    0 0.26 [0.01;   4.98]     .             .   0.26 [0.01;
4.98]     .          .    .    .
           Penicillin(1megaunit):Penicillin(3megaunits) 0    0 0.58 [0.02;  17.99]     .             .   0.58 [0.02;
17.99]     .          .    .    .
                Penicillin(3.9g):Penicillin(3megaunits) 0    0 2.24 [0.18;  28.23]     .             .   2.24 [0.18;
28.23]     .          .    .    .

Legend:
 comparison - Treatment comparison
 k          - Number of studies providing direct evidence
 prop       - Direct evidence proportion
 nma        - Estimated treatment effect (OR) in network meta-analysis
 direct     - Estimated treatment effect (OR) derived from direct evidence
 indir.     - Estimated treatment effect (OR) derived from indirect evidence
 RoR        - Ratio of Ratios (direct versus indirect)
 z          - z-value of test for disagreement (direct versus indirect)
 p-value    - p-value of test for disagreement (direct versus indirect)
>
```

FIG S10 Pairwise comparison and inconsistency test of safety for different daily dosages of oral antibiotics treating LD

Random effects model:

comparison	k	prop	nma	95%-CI	direct	95%-CI	indir.	95%-CI	RoR	95%-CI	z	p-value
Amoxicillin:Azithromycin	0	0	0.37	[0.01; 18.68]	.	.	0.37	[0.01; 18.68]	.	.	.	.
Amoxicillin:Cefotaxime	0	0	0.58	[0.01; 23.53]	.	.	0.58	[0.01; 23.53]	.	.	.	.
Amoxicillin:Ceftriaxone	0	0	0.24	[0.01; 9.74]	.	.	0.24	[0.01; 9.74]	.	.	.	.
Amoxicillin:Doxycycline	1	0.95	1.59	[0.07; 38.02]	1.72	[0.07; 44.58]	0.37	[0.00; 533621.59]	4.63	[0; 9613457.1]	0.21	0.8364
Amoxicillin:Penicillin	1	0.71	1.76	[0.06; 50.01]	1.40	[0.03; 75.42]	3.03	[0.01; 1442.92]	0.46	[0; 711.6]	-0.21	0.8364
Azithromycin:Cefotaxime	0	0	1.57	[0.06; 37.90]	.	.	1.57	[0.06; 37.90]	.	.	.	.
Azithromycin:Ceftriaxone	0	0	0.65	[0.03; 15.71]	.	.	0.65	[0.03; 15.71]	.	.	.	.
Azithromycin:Doxycycline	1	1.00	4.31	[0.43; 43.35]	4.31	[0.43; 43.35]	.	.	.	.	.	.
Azithromycin:Penicillin	0	0	4.76	[0.30; 75.39]	.	.	4.76	[0.30; 75.39]	.	.	.	.
Cefotaxime:Ceftriaxone	0	0	0.41	[0.04; 3.90]	.	.	0.41	[0.04; 3.90]	.	.	.	.
Cefotaxime:Doxycycline	0	0	2.75	[0.31; 24.69]	.	.	2.75	[0.31; 24.69]	.	.	.	.
Cefotaxime:Penicillin	1	1.00	3.03	[0.62; 14.79]	3.03	[0.62; 14.79]	.	.	.	.	.	.
Ceftriaxone:Doxycycline	0	0	6.68	[0.74; 60.60]	.	.	6.68	[0.74; 60.60]	.	.	.	.
Ceftriaxone:Penicillin	2	1.00	7.37	[1.49; 36.44]	7.37	[1.49; 36.44]	.	.	.	.	.	.
Doxycycline:Penicillin	2	1.00	1.10	[0.24; 5.04]	1.10	[0.24; 5.04]	.	.	.	.	.	.

Legend:
comparison - Treatment comparison
k - Number of studies providing direct evidence
prop - Direct evidence proportion
nma - Estimated treatment effect (OR) in network meta-analysis
direct - Estimated treatment effect (OR) derived from direct evidence
indir. - Estimated treatment effect (OR) derived from indirect evidence
RoR - Ratio of Ratios (direct versus indirect)
z - z-value of test for disagreement (direct versus indirect)
p-value - p-value of test for disagreement (direct versus indirect)

FIG S11 Pairwise comparison and inconsistency test of efficacy for antibiotics treating LA

Random effects model:

comparison	k	prop	nma	95%-CI	direct	95%-CI	indir.	95%-CI	RoR	95%-CI	z	p-value
Cefotaxime:Ceftriaxone	1	0.54	1.06	[0.23; 4.91]	0.75	[0.09; 6.00]	1.60	[0.17; 15.43]	0.47	[0.02; 10.13]	-0.48	0.6282
Cefotaxime:Doxycycline	0	0	1.16	[0.20; 6.67]	.	.	1.16	[0.20; 6.67]	.	.	.	.
Cefotaxime:Penicillin	2	0.69	2.46	[0.59; 10.23]	3.12	[0.56; 17.38]	1.46	[0.11; 18.71]	2.14	[0.10; 46.26]	0.48	0.6282
Ceftriaxone:Doxycycline	1	0.68	1.10	[0.30; 3.99]	0.54	[0.11; 2.59]	4.83	[0.50; 46.60]	0.11	[0.01; 1.75]	-1.56	0.1187
Ceftriaxone:Penicillin	2	0.44	2.32	[0.64; 8.47]	5.13	[0.73; 36.08]	1.24	[0.22; 7.01]	4.13	[0.30; 56.09]	1.07	0.2865
Doxycycline:Penicillin	2	0.65	2.11	[0.57; 7.85]	0.99	[0.19; 5.01]	8.86	[0.96; 82.25]	0.11	[0.01; 1.75]	-1.56	0.1187

Legend:
comparison - Treatment comparison
k - Number of studies providing direct evidence
prop - Direct evidence proportion
nma - Estimated treatment effect (OR) in network meta-analysis
direct - Estimated treatment effect (OR) derived from direct evidence
indir. - Estimated treatment effect (OR) derived from indirect evidence
RoR - Ratio of Ratios (direct versus indirect)
z - z-value of test for disagreement (direct versus indirect)
p-value - p-value of test for disagreement (direct versus indirect)

FIG S12 Pairwise comparison and inconsistency test of efficacy for antibiotics treating LNB

Random effects model:

comparison	k	prop	nma	95%-CI	direct	95%-CI	indir.	95%-CI	RoR	95%-CI	z	p-value
Cefotaxime(6g):Ceftriaxone(2g)	1	1.00	0.75	[0.05; 12.13]	0.75	[0.05; 12.13]	.	.	.	.	.	.
Cefotaxime(6g):Doxycycline(0.1g)	0	0	3.46	[0.12; 98.21]	.	.	3.46	[0.12; 98.21]	.	.	.	.
Cefotaxime(6g):Doxycycline(0.2g)	0	0	0.40	[0.01; 16.21]	.	.	0.40	[0.01; 16.21]	.	.	.	.
Cefotaxime(6g):Penicillin(12g)	0	0	0.54	[0.00; 192.90]	.	.	0.54	[0.00; 192.90]	.	.	.	.
Cefotaxime(6g):Penicillin(20megaunits)	2	1.00	3.24	[0.37; 28.08]	3.24	[0.37; 28.08]	.	.	.	.	.	.
Ceftriaxone(2g):Doxycycline(0.1g)	0	0	4.61	[0.06; 358.27]	.	.	4.61	[0.06; 358.27]	.	.	.	.
Ceftriaxone(2g):Doxycycline(0.2g)	1	1.00	0.54	[0.05; 6.09]	0.54	[0.05; 6.09]	.	.	.	.	.	.
Ceftriaxone(2g):Penicillin(12g)	0	0	0.72	[0.00; 127.57]	.	.	0.72	[0.00; 127.57]	.	.	.	.
Ceftriaxone(2g):Penicillin(20megaunits)	0	0	4.32	[0.13; 146.33]	.	.	4.32	[0.13; 146.33]	.	.	.	.
Doxycycline(0.1g):Doxycycline(0.2g)	0	0	0.12	[0.00; 17.03]	.	.	0.12	[0.00; 17.03]	.	.	.	.
Doxycycline(0.1g):Penicillin(12g)	0	0	0.16	[0.00; 135.17]	.	.	0.16	[0.00; 135.17]	.	.	.	.
Doxycycline(0.1g):Penicillin(20megaunits)	1	1.00	0.94	[0.07; 12.03]	0.94	[0.07; 12.03]	.	.	.	.	.	.
Doxycycline(0.2g):Penicillin(12g)	1	1.00	1.34	[0.01; 129.51]	1.34	[0.01; 129.51]	.	.	.	.	.	.
Doxycycline(0.2g):Penicillin(20megaunits)	0	0	8.01	[0.11; 577.85]	.	.	8.01	[0.11; 577.85]	.	.	.	.
Penicillin(12g):Penicillin(20megaunits)	0	0	5.98	[0.01; 3129.88]	.	.	5.98	[0.01; 3129.88]	.	.	.	.

Legend:
comparison - Treatment comparison
k - Number of studies providing direct evidence
prop - Direct evidence proportion
nma - Estimated treatment effect (OR) in network meta-analysis
direct - Estimated treatment effect (OR) derived from direct evidence
indir. - Estimated treatment effect (OR) derived from indirect evidence
RoR - Ratio of Ratios (direct versus indirect)
z - z-value of test for disagreement (direct versus indirect)
p-value - p-value of test for disagreement (direct versus indirect)

FIG S13 Pairwise comparison and inconsistency test of efficacy for different daily dosages of antibiotics treating LNB

Random effects model:

comparison	k	prop	nma	95%-CI	direct	95%-CI	indir.	95%-CI	RoR	95%-CI	z	p-value
Amoxicillin:Amoxicillin+Probenecid	0	0	2.07	[0.35; 12.25]	.	.	2.07	[0.35; 12.25]	.	.	.	.
Amoxicillin:Azithromycin	1	0.92	2.20	[0.88; 5.49]	2.30	[0.88; 5.98]	1.28	[0.05; 33.44]	1.80	[0.06; 54.05]	0.34	0.7348
Amoxicillin:Ceftriaxone	0	0	2.51	[0.46; 13.60]	.	.	2.51	[0.46; 13.60]	.	.	.	.
Amoxicillin:Ceftriaxone+Doxycycline	0	0	5.44	[1.19; 24.92]	.	.	5.44	[1.19; 24.92]	.	.	.	.
Amoxicillin:Cefuroxime	0	0	5.51	[1.18; 25.66]	.	.	5.51	[1.18; 25.66]	.	.	.	.
Amoxicillin:Doxycycline	1	0.09	4.70	[1.42; 15.60]	0.93	[0.02; 49.81]	5.53	[1.57; 19.44]	0.17	[0.00; 10.94]	-0.84	0.4023
Amoxicillin:Erythromycin	0	0	2.41	[0.47; 12.49]	.	.	2.41	[0.47; 12.49]	.	.	.	.
Amoxicillin:Minocycline	0	0	4.54	[0.07; 304.14]	.	.	4.54	[0.07; 304.14]	.	.	.	.
Amoxicillin:Penicillin	1	0.14	3.91	[1.14; 13.46]	3.50	[0.13; 92.97]	3.98	[1.05; 15.14]	0.88	[0.03; 30.30]	-0.07	0.9423
Amoxicillin:Tetracycline	0	0	1.50	[0.33; 6.91]	.	.	1.50	[0.33; 6.91]	.	.	.	.
Amoxicillin+Probenecid:Azithromycin	1	0.68	1.06	[0.23; 4.93]	1.23	[0.19; 7.96]	0.77	[0.05; 11.63]	1.59	[0.06; 42.75]	0.28	0.7823
Amoxicillin+Probenecid:Ceftriaxone	0	0	1.21	[0.17; 8.88]	.	.	1.21	[0.17; 8.88]	.	.	.	.
Amoxicillin+Probenecid:Ceftriaxone+Doxycycline	0	0	2.63	[0.47; 14.83]	.	.	2.63	[0.47; 14.83]	.	.	.	.
Amoxicillin+Probenecid:Cefuroxime	0	0	2.66	[0.47; 15.24]	.	.	2.66	[0.47; 15.24]	.	.	.	.
Amoxicillin+Probenecid:Doxycycline	2	0.91	2.27	[0.53; 9.73]	2.20	[0.48; 10.08]	3.18	[0.02; 436.49]	0.69	[0.00; 119.18]	-0.14	0.8879
Amoxicillin+Probenecid:Erythromycin	0	0	1.16	[0.16; 8.29]	.	.	1.16	[0.16; 8.29]	.	.	.	.
Amoxicillin+Probenecid:Minocycline	0	0	2.19	[0.03; 169.05]	.	.	2.19	[0.03; 169.05]	.	.	.	.
Amoxicillin+Probenecid:Penicillin	0	0	1.89	[0.36; 9.86]	.	.	1.89	[0.36; 9.86]	.	.	.	.
Amoxicillin+Probenecid:Tetracycline	0	0	0.73	[0.11; 4.61]	.	.	0.73	[0.11; 4.61]	.	.	.	.
Azithromycin:Ceftriaxone	0	0	1.15	[0.27; 4.90]	.	.	1.15	[0.27; 4.90]	.	.	.	.
Azithromycin:Ceftriaxone+Doxycycline	0	0	2.48	[0.72; 8.58]	.	.	2.48	[0.72; 8.58]	.	.	.	.
Azithromycin:Cefuroxime	0	0	2.51	[0.71; 8.87]	.	.	2.51	[0.71; 8.87]	.	.	.	.
Azithromycin:Doxycycline	4	0.82	2.14	[0.95; 4.84]	2.87	[1.17; 7.06]	0.55	[0.08; 3.81]	5.23	[0.62; 44.19]	1.52	0.1289
Azithromycin:Erythromycin	0	0	1.10	[0.27; 4.47]	.	.	1.10	[0.27; 4.47]	.	.	.	.
Azithromycin:Minocycline	0	0	2.07	[0.03; 126.92]	.	.	2.07	[0.03; 126.92]	.	.	.	.
Azithromycin:Penicillin	2	0.70	1.78	[0.73; 4.36]	1.44	[0.50; 4.19]	2.93	[0.57; 15.10]	0.49	[0.07; 3.48]	-0.71	0.4775
Azithromycin:Tetracycline	0	0	0.68	[0.19; 2.41]	.	.	0.68	[0.19; 2.41]	.	.	.	.
Ceftriaxone:Ceftriaxone+Doxycycline	0	0	2.17	[0.39; 11.88]	.	.	2.17	[0.39; 11.88]	.	.	.	.
Ceftriaxone:Cefuroxime	0	0	2.19	[0.39; 12.21]	.	.	2.19	[0.39; 12.21]	.	.	.	.
Ceftriaxone:Doxycycline	1	0.25	1.87	[0.45; 7.75]	0.92	[0.05; 16.13]	2.36	[0.46; 12.14]	0.39	[0.01; 10.58]	-0.56	0.5764
Ceftriaxone:Erythromycin	0	0	0.96	[0.18; 5.00]	.	.	0.96	[0.18; 5.00]	.	.	.	.
Ceftriaxone:Minocycline	0	0	1.81	[0.03; 120.36]	.	.	1.81	[0.03; 120.36]	.	.	.	.
Ceftriaxone:Penicillin	1	0.84	1.56	[0.46; 5.26]	1.81	[0.48; 6.88]	0.71	[0.03; 14.48]	2.56	[0.09; 69.38]	0.56	0.5764
Ceftriaxone:Tetracycline	0	0	0.60	[0.13; 2.82]	.	.	0.60	[0.13; 2.82]	.	.	.	.
Ceftriaxone+Doxycycline:Cefuroxime	0	0	1.01	[0.26; 3.88]	.	.	1.01	[0.26; 3.88]	.	.	.	.
Ceftriaxone+Doxycycline:Doxycycline	1	1.00	0.86	[0.34; 2.20]	0.86	[0.34; 2.20]	.	.	.	.	.	.
Ceftriaxone+Doxycycline:Erythromycin	0	0	0.44	[0.08; 2.37]	.	.	0.44	[0.08; 2.37]	.	.	.	.
Ceftriaxone+Doxycycline:Minocycline	0	0	0.83	[0.01; 57.08]	.	.	0.83	[0.01; 57.08]	.	.	.	.
Ceftriaxone+Doxycycline:Penicillin	0	0	0.72	[0.19; 2.65]	.	.	0.72	[0.19; 2.65]	.	.	.	.
Ceftriaxone+Doxycycline:Tetracycline	0	0	0.28	[0.06; 1.28]	.	.	0.28	[0.06; 1.28]	.	.	.	.
Cefuroxime:Doxycycline	2	1.00	0.85	[0.33; 2.23]	0.85	[0.33; 2.23]	.	.	.	.	.	.
Cefuroxime:Erythromycin	0	0	0.44	[0.08; 2.37]	.	.	0.44	[0.08; 2.37]	.	.	.	.
Cefuroxime:Minocycline	0	0	0.82	[0.01; 56.69]	.	.	0.82	[0.01; 56.69]	.	.	.	.
Cefuroxime:Penicillin	0	0	0.71	[0.19; 2.67]	.	.	0.71	[0.19; 2.67]	.	.	.	.
Cefuroxime:Tetracycline	0	0	0.27	[0.06; 1.29]	.	.	0.27	[0.06; 1.29]	.	.	.	.
Doxycycline:Erythromycin	0	0	0.51	[0.13; 2.06]	.	.	0.51	[0.13; 2.06]	.	.	.	.
Doxycycline:Minocycline	0	0	0.97	[0.02; 59.52]	.	.	0.97	[0.02; 59.52]	.	.	.	.
Doxycycline:Penicillin	2	0.49	0.83	[0.33; 2.07]	2.33	[0.63; 8.53]	0.31	[0.08; 1.10]	7.60	[1.23; 47.20]	2.18	0.0294
Doxycycline:Tetracycline	1	0.31	0.32	[0.09; 1.08]	0.10	[0.01; 0.88]	0.54	[0.13; 2.37]	0.18	[0.01; 2.53]	-1.27	0.2048
Erythromycin:Minocycline	0	0	1.88	[0.03; 122.23]	.	.	1.88	[0.03; 122.23]	.	.	.	.
Erythromycin:Penicillin	1	0.96	1.62	[0.53; 5.00]	1.40	[0.44; 4.42]	60.48	[0.20; 18200.95]	0.02	[0.00; 7.81]	-1.27	0.2048
Erythromycin:Tetracycline	1	0.96	0.62	[0.20; 1.92]	0.72	[0.23; 2.28]	0.02	[0.00; 5.02]	43.13	[0.13; 14522.49]	1.27	0.2048
Minocycline:Penicillin	1	1.00	0.86	[0.02; 47.82]	0.86	[0.02; 47.82]	.	.	.	.	.	.
Minocycline:Tetracycline	0	0	0.33	[0.01; 20.72]	.	.	0.33	[0.01; 20.72]	.	.	.	.
Penicillin:Tetracycline	1	0.83	0.38	[0.14; 1.04]	0.52	[0.17; 1.53]	0.09	[0.01; 1.03]	5.46	[0.40; 75.21]	1.27	0.2048

Legend:

- comparison - Treatment comparison
- k - Number of studies providing direct evidence
- prop - Direct evidence proportion
- nma - Estimated treatment effect (OR) in network meta-analysis
- direct - Estimated treatment effect (OR) derived from direct evidence
- indir. - Estimated treatment effect (OR) derived from indirect evidence
- RoR - Ratio of Ratios (direct versus indirect)
- z - z-value of test for disagreement (direct versus indirect)
- p-value - p-value of test for disagreement (direct versus indirect)

FIG S14 Pairwise comparison and inconsistency test of efficacy for antibiotics treating EM

```
Random effects model:

                                          comparison k prop   nma          95%-CI direct        95%-CI indir.          95%-CI   RoR
95%-CI     z p-value
     Amoxicillin(1.5g):Amoxicillin(1.5g)+Probenecid(1.5g) 0    0  0.41 [0.01;   26.87]      .              .  0.41 [0.01;
26.87]     .
                    Amoxicillin(1.5g):Azithromycin(0.25g) 0    0  0.47 [0.01;   27.85]      .              .  0.47 [0.01;
27.85]     .
                     Amoxicillin(1.5g):Azithromycin(0.5g) 1 1.00  2.30 [1.11;    4.75]   2.30 [1.11;
4.75]      .
                       Amoxicillin(1.5g):Ceftriaxone(1g) 0    0  0.81 [0.12;    5.28]      .              .  0.81 [0.12;
5.28]     .
                       Amoxicillin(1.5g):Ceftriaxone(2g) 0    0  1.01 [0.01;  125.60]      .              .  1.01 [0.01;
125.60]    .
                     Amoxicillin(1.5g):Doxycycline(0.2g) 1 1.00  0.93 [0.02;   47.42]   0.93 [0.02;
47.42]     .
                    Amoxicillin(1.5g):Doxycycline(0.2g)   0    0 10.91 [2.43;   49.03]      .              . 10.91 [2.43;
49.03]     .
                  Amoxicillin(1.5g):Penicillin(1megaunit) 0    0  1.98 [0.03;  121.32]      .              .  1.98 [0.03;
121.32]    .
                      Amoxicillin(1.5g):Penicillin(3.9g) 1 1.00  3.50 [0.14;   87.57]   3.50 [0.14;
87.57]     .
                 Amoxicillin(1.5g):Penicillin(3megaunits) 0    0  1.47 [0.34;    6.32]      .              .  1.47 [0.34;
6.32]     .
   Amoxicillin(1.5g)+Probenecid(1.5g):Azithromycin(0.25g) 1 0.77  1.13 [0.24;    5.33]   1.23 [0.21;  7.15]   0.86 [0.03;    22.05]  1.44 [0.04;
57.87]  0.19  0.8472
    Amoxicillin(1.5g)+Probenecid(1.5g):Azithromycin(0.5g) 0    0  5.55 [0.08;  382.62]      .              .  5.55 [0.08;
382.62]    .
       Amoxicillin(1.5g)+Probenecid(1.5g):Ceftriaxone(1g) 0    0  1.95 [0.02;  189.07]      .              .  1.95 [0.02;
189.07]    .
       Amoxicillin(1.5g)+Probenecid(1.5g):Ceftriaxone(2g) 0    0  2.43 [0.11;   54.95]      .              .  2.43 [0.11;
54.95]     .
     Amoxicillin(1.5g)+Probenecid(1.5g):Doxycycline(0.2g) 2 0.95  2.24 [0.56;    8.94]   2.23 [0.54;  9.23]   2.41 [0.01;  1087.63]  0.92 [0.00;
490.68] -0.02  0.9802
   Amoxicillin(1.5g)+Probenecid(1.5g):Doxycycline(0.2g)   0    0 26.30 [0.31; 2216.63]      .              . 26.30 [0.31;
2216.63]    .
  Amoxicillin(1.5g)+Probenecid(1.5g):Penicillin(1megaunit) 0    0  4.78 [0.82;   27.90]      .              .  4.78 [0.82;
27.90]     .
      Amoxicillin(1.5g)+Probenecid(1.5g):Penicillin(3.9g) 0    0  8.43 [0.25;  280.77]      .              .  8.43 [0.25;
280.77]    .
 Amoxicillin(1.5g)+Probenecid(1.5g):Penicillin(3megaunits) 0    0  3.54 [0.04;  293.84]      .              .  3.54 [0.04;
293.84]    .
                   Azithromycin(0.25g):Azithromycin(0.5g) 0    0  4.89 [0.08;  309.03]      .              .  4.89 [0.08;
309.03]    .
                      Azithromycin(0.25g):Ceftriaxone(1g) 0    0  1.72 [0.02;  153.73]      .              .  1.72 [0.02;
153.73]    .
                      Azithromycin(0.25g):Ceftriaxone(2g) 0    0  2.14 [0.11;   42.96]      .              .  2.14 [0.11;
42.96]     .
                    Azithromycin(0.25g):Doxycycline(0.2g) 2 0.99  1.97 [0.67;    5.84]   2.01 [0.68;  5.98]   0.16 [0.00; 39706.49] 12.55 [0.00;
3260978.07]  0.40  0.6909
                  Azithromycin(0.25g):Doxycycline(0.2g)   0    0 23.21 [0.30; 1797.62]      .              . 23.21 [0.30;
1797.62]    .
                Azithromycin(0.25g):Penicillin(1megaunit) 1 0.79  4.21 [1.11;   15.95]   4.25 [0.95; 19.07]   4.08 [0.23;    72.75]  1.04 [0.04;
26.84]  0.02  0.9802
                    Azithromycin(0.25g):Penicillin(3.9g) 0    0  7.44 [0.25;  222.53]      .              .  7.44 [0.25;
222.53]    .
              Azithromycin(0.25g):Penicillin(3megaunits) 0    0  3.12 [0.04;  238.23]      .              .  3.12 [0.04;
238.23]    .
                       Azithromycin(0.5g):Ceftriaxone(1g) 0    0  0.35 [0.06;    1.99]      .              .  0.35 [0.06;
1.99]     .
                       Azithromycin(0.5g):Ceftriaxone(2g) 0    0  0.44 [0.00;   57.66]      .              .  0.44 [0.00;
57.66]     .
                     Azithromycin(0.5g):Doxycycline(0.2g) 0    0  0.40 [0.01;   22.03]      .              .  0.40 [0.01;
22.03]     .
                    Azithromycin(0.5g):Doxycycline(0.2g)   2 1.00  4.74 [1.27;   17.70]   4.74 [1.27;
17.70]     .
                 Azithromycin(0.5g):Penicillin(1megaunit) 0    0  0.86 [0.01;   56.21]      .              .  0.86 [0.01;
56.21]     .
                      Azithromycin(0.5g):Penicillin(3.9g) 0    0  1.52 [0.06;   41.27]      .              .  1.52 [0.06;
41.27]     .
               Azithromycin(0.5g):Penicillin(3megaunits) 1 1.00  0.64 [0.18;    2.27]   0.64 [0.18;
2.27]      .
                         Ceftriaxone(1g):Ceftriaxone(2g) 0    0  1.25 [0.01;  220.80]      .              .  1.25 [0.01;
220.80]    .
                       Ceftriaxone(1g):Doxycycline(0.2g) 0    0  1.15 [0.01;   89.63]      .              .  1.15 [0.01;
89.63]     .
                     Ceftriaxone(1g):Doxycycline(0.2g)   0    0 13.48 [1.53;  118.67]      .              . 13.48 [1.53;
118.67]    .
                    Ceftriaxone(1g):Penicillin(1megaunit) 0    0  2.45 [0.03;  225.47]      .              .  2.45 [0.03;
225.47]    .
                         Ceftriaxone(1g):Penicillin(3.9g) 0    0  4.32 [0.10;  179.70]      .              .  4.32 [0.10;
179.70]    .
                  Ceftriaxone(1g):Penicillin(3megaunits) 1 1.00  1.81 [0.56;    5.89]   1.81 [0.56;
5.89]      .
                       Ceftriaxone(2g):Doxycycline(0.2g) 1 1.00  0.92 [0.06;   15.06]   0.92 [0.06;
15.06]     .
                     Ceftriaxone(2g):Doxycycline(0.2g)   0    0 10.83 [0.07; 1698.08]      .              . 10.83 [0.07;
1698.08]    .
                    Ceftriaxone(2g):Penicillin(1megaunit) 0    0  1.97 [0.09;   41.26]      .              .  1.97 [0.09;
41.26]     .
                         Ceftriaxone(2g):Penicillin(3.9g) 0    0  3.47 [0.05;  246.76]      .              .  3.47 [0.05;
246.76]    .
                  Ceftriaxone(2g):Penicillin(3megaunits) 0    0  1.46 [0.01;  225.50]      .              .  1.46 [0.01;
225.50]    .
                    Doxycycline(0.2g):Doxycycline(0.2g)   0    0 11.76 [0.17;  794.06]      .              . 11.76 [0.17;
794.06]    .
                  Doxycycline(0.2g):Penicillin(1megaunit) 1 0.90  2.14 [0.64;    7.13]   2.12 [0.60;  7.57]   2.24 [0.05;   104.09]  0.95 [0.02;
54.25] -0.02  0.9802
                       Doxycycline(0.2g):Penicillin(3.9g) 1 1.00  3.77 [0.15;   94.40]   3.77 [0.15;
94.40]     .
                Doxycycline(0.2g):Penicillin(3megaunits) 0    0  1.58 [0.02;  105.18]      .              .  1.58 [0.02;
105.18]    .
               Doxycycline(0.2g)  :Penicillin(1megaunit) 0    0  0.18 [0.00;   14.51]      .              .  0.18 [0.00;
14.51]     .
                    Doxycycline(0.2g)  :Penicillin(3.9g) 0    0  0.32 [0.01;   11.21]      .              .  0.32 [0.01;
11.21]     .
              Doxycycline(0.2g)  :Penicillin(3megaunits) 0    0  0.13 [0.02;    0.84]      .              .  0.13 [0.02;
0.84]     .
                  Penicillin(1megaunit):Penicillin(3.9g) 0    0  1.77 [0.06;   54.99]      .              .  1.77 [0.06;
54.99]     .
             Penicillin(1megaunit):Penicillin(3megaunits) 0    0  0.74 [0.01;   58.36]      .              .  0.74 [0.01;
58.36]     .
                 Penicillin(3.9g):Penicillin(3megaunits) 0    0  0.42 [0.01;   14.41]      .              .  0.42 [0.01;
14.41]     .

Legend:
 comparison - Treatment comparison
 k          - Number of studies providing direct evidence
 prop       - Direct evidence proportion
 nma        - Estimated treatment effect (OR) in network meta-analysis
 direct     - Estimated treatment effect (OR) derived from direct evidence
 indir.     - Estimated treatment effect (OR) derived from indirect evidence
 RoR        - Ratio of Ratios (direct versus indirect)
 z          - z-value of test for disagreement (direct versus indirect)
 p-value    - p-value of test for disagreement (direct versus indirect)
>
```

FIG S15 Pairwise comparison and inconsistency test of efficacy for different daily dosages of antibiotics treating EM

Random effects model:

comparison	k	prop	nma	95%-CI	direct	95%-CI	indir.	95%-CI	Diff	95%-CI	z	p-value
Amoxicillin:Azithromycin	0	0	5.48	[-9.63; 20.59]	.	.	5.48	[-9.63; 20.59]	.	.	.	.
Amoxicillin:Doxycycline	1	0.35	2.78	[-12.18; 17.74]	-9.65	[-34.95; 15.65]	9.46	[-9.08; 28.01]	-19.11	[-50.49; 12.26]	-1.19	0.2324
Amoxicillin:Erythromycin	0	0	2.19	[-13.50; 17.89]	.	.	2.19	[-13.50; 17.89]	.	.	.	.
Amoxicillin:Penicillin	1	0.98	4.86	[-9.56; 19.29]	6.15	[-8.43; 20.73]	-55.13	[-154.66; 44.39]	61.28	[-39.31; 161.87]	1.19	0.2324
Amoxicillin:Tetracycline	0	0	6.89	[-8.19; 21.96]	.	.	6.89	[-8.19; 21.96]	.	.	.	.
Azithromycin:Doxycycline	3	0.97	-2.70	[-6.01; 0.62]	-2.18	[-5.54; 1.19]	-19.25	[-38.29; -0.21]	17.07	[-2.26; 36.41]	1.73	0.0835
Azithromycin:Erythromycin	0	0	-3.29	[-10.65; 4.07]	.	.	-3.29	[-10.65; 4.07]	.	.	.	.
Azithromycin:Penicillin	1	0.62	-0.62	[-5.44; 4.21]	-1.90	[-8.01; 4.21]	1.51	[-6.35; 9.37]	-3.41	[-13.37; 6.54]	-0.67	0.5015
Azithromycin:Tetracycline	0	0	1.41	[-3.81; 6.63]	.	.	1.41	[-3.81; 6.63]	.	.	.	.
Doxycycline:Erythromycin	0	0	-0.59	[-7.55; 6.37]	.	.	-0.59	[-7.55; 6.37]	.	.	.	.
Doxycycline:Penicillin	2	0.58	2.08	[-2.32; 6.48]	-0.59	[-6.37; 5.18]	5.76	[-1.01; 12.54]	-6.36	[-15.26; 2.55]	-1.40	0.1618
Doxycycline:Tetracycline	1	0.68	4.10	[-0.32; 8.52]	6.10	[0.74; 11.46]	-0.17	[-8.01; 7.66]	6.27	[-3.22; 15.77]	1.30	0.1952
Erythromycin:Penicillin	1	0.93	2.67	[-3.59; 8.93]	3.80	[-2.69; 10.29]	-12.49	[-36.27; 11.29]	16.29	[-8.36; 40.94]	1.30	0.1952
Erythromycin:Tetracycline	1	0.92	4.69	[-1.61; 11.00]	3.50	[-3.06; 10.06]	19.24	[-3.65; 42.13]	-15.74	[-39.55; 8.07]	-1.30	0.1952
Penicillin:Tetracycline	1	0.63	2.02	[-2.56; 6.61]	-0.30	[-6.08; 5.48]	5.97	[-1.56; 13.51]	-6.27	[-15.77; 3.22]	-1.30	0.1952

Legend:
- comparison - Treatment comparison
- k - Number of studies providing direct evidence
- prop - Direct evidence proportion
- nma - Estimated treatment effect (MD) in network meta-analysis
- direct - Estimated treatment effect (MD) derived from direct evidence
- indir. - Estimated treatment effect (MD) derived from indirect evidence
- Diff - Difference between direct and indirect treatment estimates
- z - z-value of test for disagreement (direct versus indirect)
- p-value - p-value of test for disagreement (direct versus indirect)

FIG S16 Pairwise comparison and inconsistency test of therapy duration for antibiotics treating EM

Random effects model:

comparison	k	prop	nma	95%-CI	direct	95%-CI	indir.	95%-CI	RoR	95%-CI	z	p-value
Amoxicillin:Amoxicillin+Probenecid	0	0	0.31	[0.08; 1.22]	.	.	0.31	[0.08; 1.22]	.	.	.	.
Amoxicillin:Azithromycin	1	0.63	0.75	[0.38; 1.45]	0.59	[0.25; 1.35]	1.13	[0.38; 3.38]	0.52	[0.13; 2.06]	-0.93	0.3522
Amoxicillin:Ceftriaxone	0	0	0.47	[0.15; 1.44]	.	.	0.47	[0.15; 1.44]	.	.	.	.
Amoxicillin:Ceftriaxone+Doxycycline	0	0	0.40	[0.13; 1.28]	.	.	0.40	[0.13; 1.28]	.	.	.	.
Amoxicillin:Cefuroxime	0	0	1.27	[0.48; 3.40]	.	.	1.27	[0.48; 3.40]	.	.	.	.
Amoxicillin:Doxycycline	1	0.62	0.85	[0.41; 1.76]	1.39	[0.55; 3.53]	0.38	[0.12; 1.25]	3.63	[0.81; 16.27]	1.68	0.0921
Amoxicillin:Minocycline	0	0	0.17	[0.03; 1.03]	.	.	0.17	[0.03; 1.03]	.	.	.	.
Amoxicillin:Penicillin	1	0.68	1.48	[0.67; 3.24]	1.38	[0.53; 3.58]	1.72	[0.43; 6.88]	0.80	[0.15; 4.32]	-0.26	0.7964
Amoxicillin:Tetracycline	0	0	0.63	[0.17; 2.43]	.	.	0.63	[0.17; 2.43]	.	.	.	.
Amoxicillin+Probenecid:Azithromycin	1	0.60	2.44	[0.66; 8.93]	3.15	[0.59; 16.77]	1.65	[0.21; 12.95]	1.91	[0.13; 27.23]	0.48	0.6318
Amoxicillin+Probenecid:Ceftriaxone	0	0	1.52	[0.32; 7.12]	.	.	1.52	[0.32; 7.12]	.	.	.	.
Amoxicillin+Probenecid:Ceftriaxone+Doxycycline	0	0	1.32	[0.28; 6.24]	.	.	1.32	[0.28; 6.24]	.	.	.	.
Amoxicillin+Probenecid:Cefuroxime	0	0	4.15	[0.99; 17.43]	.	.	4.15	[0.99; 17.43]	.	.	.	.
Amoxicillin+Probenecid:Doxycycline	2	0.74	2.78	[0.78; 9.93]	2.84	[0.65; 12.48]	2.60	[0.21; 31.78]	1.10	[0.06; 20.07]	0.06	0.9512
Amoxicillin+Probenecid:Minocycline	0	0	0.57	[0.07; 4.78]	.	.	0.57	[0.07; 4.78]	.	.	.	.
Amoxicillin+Probenecid:Penicillin	0	0	4.82	[1.17; 19.90]	.	.	4.82	[1.17; 19.90]	.	.	.	.
Amoxicillin+Probenecid:Tetracycline	0	0	2.07	[0.38; 11.34]	.	.	2.07	[0.38; 11.34]	.	.	.	.
Azithromycin:Ceftriaxone	0	0	0.62	[0.21; 1.87]	.	.	0.62	[0.21; 1.87]	.	.	.	.
Azithromycin:Ceftriaxone+Doxycycline	0	0	0.54	[0.18; 1.66]	.	.	0.54	[0.18; 1.66]	.	.	.	.
Azithromycin:Cefuroxime	0	0	1.71	[0.66; 4.39]	.	.	1.71	[0.66; 4.39]	.	.	.	.
Azithromycin:Doxycycline	4	0.58	1.14	[0.58; 2.25]	0.76	[0.31; 1.86]	2.00	[0.70; 5.71]	0.38	[0.10; 1.51]	-1.37	0.1703
Azithromycin:Minocycline	0	0	0.23	[0.04; 1.37]	.	.	0.23	[0.04; 1.37]	.	.	.	.
Azithromycin:Penicillin	2	0.43	1.98	[0.91; 4.31]	2.96	[0.90; 9.70]	1.46	[0.52; 4.10]	2.03	[0.42; 9.78]	0.88	0.3791
Azithromycin:Tetracycline	0	0	0.85	[0.23; 3.17]	.	.	0.85	[0.23; 3.17]	.	.	.	.
Ceftriaxone:Ceftriaxone+Doxycycline	0	0	0.87	[0.25; 3.03]	.	.	0.87	[0.25; 3.03]	.	.	.	.
Ceftriaxone:Cefuroxime	0	0	2.73	[0.91; 8.21]	.	.	2.73	[0.91; 8.21]	.	.	.	.
Ceftriaxone:Doxycycline	1	0.93	1.83	[0.76; 4.41]	1.78	[0.71; 4.44]	2.56	[0.10; 64.52]	0.70	[0.02; 19.95]	-0.21	0.8323
Ceftriaxone:Minocycline	0	0	0.37	[0.05; 2.61]	.	.	0.37	[0.05; 2.61]	.	.	.	.
Ceftriaxone:Penicillin	1	0.13	3.17	[1.03; 9.72]	4.35	[0.19; 99.93]	3.03	[0.91; 10.05]	1.44	[0.05; 41.19]	0.21	0.8323
Ceftriaxone:Tetracycline	0	0	1.36	[0.33; 5.69]	.	.	1.36	[0.33; 5.69]	.	.	.	.
Ceftriaxone+Doxycycline:Cefuroxime	0	0	3.15	[1.04; 9.52]	.	.	3.15	[1.04; 9.52]	.	.	.	.
Ceftriaxone+Doxycycline:Doxycycline	1	1.00	2.10	[0.86; 5.12]	2.10	[0.86; 5.12]	.	.	.	.	.	.
Ceftriaxone+Doxycycline:Minocycline	0	0	0.43	[0.06; 3.09]	.	.	0.43	[0.06; 3.09]	.	.	.	.
Ceftriaxone+Doxycycline:Penicillin	0	0	3.65	[1.14; 11.71]	.	.	3.65	[1.14; 11.71]	.	.	.	.
Ceftriaxone+Doxycycline:Tetracycline	0	0	1.57	[0.37; 6.59]	.	.	1.57	[0.37; 6.59]	.	.	.	.
Cefuroxime:Doxycycline	2	1.00	0.67	[0.35; 1.29]	0.67	[0.35; 1.29]	.	.	.	.	.	.
Cefuroxime:Minocycline	0	0	0.14	[0.02; 0.90]	.	.	0.14	[0.02; 0.90]	.	.	.	.
Cefuroxime:Penicillin	0	0	1.16	[0.43; 3.16]	.	.	1.16	[0.43; 3.16]	.	.	.	.
Cefuroxime:Tetracycline	0	0	0.50	[0.14; 1.84]	.	.	0.50	[0.14; 1.84]	.	.	.	.
Doxycycline:Minocycline	0	0	0.20	[0.04; 1.19]	.	.	0.20	[0.04; 1.19]	.	.	.	.
Doxycycline:Penicillin	2	0.73	1.74	[0.82; 3.69]	1.26	[0.52; 3.05]	4.15	[0.96; 17.89]	0.30	[0.06; 1.68]	-1.37	0.1716
Doxycycline:Tetracycline	1	1.00	0.75	[0.24; 2.30]	0.75	[0.24; 2.30]	.	.	.	.	.	.
Minocycline:Penicillin	1	1.00	8.50	[1.73; 41.78]	8.50	[1.73; 41.78]	.	.	.	.	.	.
Minocycline:Tetracycline	0	0	3.65	[0.45; 29.57]	.	.	3.65	[0.45; 29.57]	.	.	.	.
Penicillin:Tetracycline	0	0	0.43	[0.11; 1.67]	.	.	0.43	[0.11; 1.67]	.	.	.	.

Legend:
- comparison - Treatment comparison
- k - Number of studies providing direct evidence
- prop - Direct evidence proportion
- nma - Estimated treatment effect (OR) in network meta-analysis
- direct - Estimated treatment effect (OR) derived from direct evidence
- indir. - Estimated treatment effect (OR) derived from indirect evidence
- RoR - Ratio of Ratios (direct versus indirect)
- z - z-value of test for disagreement (direct versus indirect)
- p-value - p-value of test for disagreement (direct versus indirect)

>

FIG S17 Pairwise comparison and inconsistency test of safety for antibiotics treating EM

Random effects model:

comparison	k	prop	nma	95%-CI	direct	95%-CI	indir.	95%-CI	RoR	95%-CI	z	p-value
Amoxicillin(1.5g):Amoxicillin(1.5g)+Probenecid(1.5g)	0	0	0.65	[0.09; 4.49]	.		0.65	[0.09; 4.49]	.		.	.
Amoxicillin(1.5g):Azithromycin(0.25g)	0	0	2.01	[0.30; 13.45]	.		2.01	[0.30; 13.45]	.		.	.
Amoxicillin(1.5g):Azithromycin(0.5g)	1	1.00	0.59	[0.18; 1.90]	0.59	[0.18; 1.90]	.		.		.	.
Amoxicillin(1.5g):Ceftriaxone(1g)	0	0	0.43	[0.01; 19.09]	.		0.43	[0.01; 19.09]	.		.	.
Amoxicillin(1.5g):Ceftriaxone(2g)	0	0	0.78	[0.14; 4.51]	.		0.78	[0.14; 4.51]	.		.	.
Amoxicillin(1.5g):Doxycycline(0.2g)	1	1.00	1.39	[0.40; 4.83]	1.39	[0.40; 4.83]	.		.		.	.
Amoxicillin(1.5g):Doxycycline(0.2g)	0	0	0.40	[0.07; 2.30]	.		0.40	[0.07; 2.30]	.		.	.
Amoxicillin(1.5g):Penicillin(1megaunit)	0	0	6.34	[0.42; 95.47]	.		6.34	[0.42; 95.47]	.		.	.
Amoxicillin(1.5g):Penicillin(3.9g)	1	1.00	1.38	[0.39; 4.86]	1.38	[0.39; 4.86]	.		.		.	.
Amoxicillin(1.5g):Penicillin(3megaunits)	0	0	1.88	[0.26; 13.39]	.		1.88	[0.26; 13.39]	.		.	.
Amoxicillin(1.5g)+Probenecid(1.5g):Azithromycin(0.25g)	1	0.75	3.10	[0.62; 15.53]	3.15	[0.49; 20.33]	2.97	[0.12; 72.38]	1.06	[0.03; 42.83]	0.03	0.9749
Amoxicillin(1.5g)+Probenecid(1.5g):Azithromycin(0.5g)	0	0	0.91	[0.09; 8.76]	.		0.91	[0.09; 8.76]	.		.	.
Amoxicillin(1.5g)+Probenecid(1.5g):Ceftriaxone(1g)	0	0	0.67	[0.01; 47.14]	.		0.67	[0.01; 47.14]	.		.	.
Amoxicillin(1.5g)+Probenecid(1.5g):Ceftriaxone(2g)	0	0	1.21	[0.18; 8.38]	.		1.21	[0.18; 8.38]	.		.	.
Amoxicillin(1.5g)+Probenecid(1.5g):Doxycycline(0.2g)	2	0.87	2.16	[0.49; 9.56]	2.70	[0.55; 13.35]	0.51	[0.01; 29.78]	5.28	[0.07; 416.85]	0.75	0.4553
Amoxicillin(1.5g)+Probenecid(1.5g):Doxycycline(0.2g)	0	0	0.61	[0.04; 8.41]	.		0.61	[0.04; 8.41]	.		.	.
Amoxicillin(1.5g)+Probenecid(1.5g):Penicillin(1megaunit)	0	0	9.82	[0.65; 149.24]	.		9.82	[0.65; 149.24]	.		.	.
Amoxicillin(1.5g)+Probenecid(1.5g):Penicillin(3.9g)	0	0	2.13	[0.30; 14.96]	.		2.13	[0.30; 14.96]	.		.	.
Amoxicillin(1.5g)+Probenecid(1.5g):Penicillin(3megaunits)	0	0	2.90	[0.18; 45.91]	.		2.90	[0.18; 45.91]	.		.	.
Azithromycin(0.25g):Azithromycin(0.5g)	0	0	0.29	[0.03; 2.74]	.		0.29	[0.03; 2.74]	.		.	.
Azithromycin(0.25g):Ceftriaxone(1g)	0	0	0.22	[0.00; 14.94]	.		0.22	[0.00; 14.94]	.		.	.
Azithromycin(0.25g):Ceftriaxone(2g)	0	0	0.39	[0.06; 2.61]	.		0.39	[0.06; 2.61]	.		.	.
Azithromycin(0.25g):Doxycycline(0.2g)	2	0.93	0.70	[0.16; 2.94]	0.91	[0.20; 4.04]	0.02	[0.00; 4.43]	53.48	[0.17; 16989.89]	1.35	0.1758
Azithromycin(0.25g):Doxycycline(0.2g)	0	0	0.20	[0.01; 2.64]	.		0.20	[0.01; 2.64]	.		.	.
Azithromycin(0.25g):Penicillin(1megaunit)	1	0.88	3.16	[0.25; 39.54]	2.22	[0.15; 32.75]	43.65	[0.03; 67236.71]	0.05	[0.00; 126.41]	-0.75	0.4553
Azithromycin(0.25g):Penicillin(3.9g)	0	0	0.69	[0.10; 4.65]	.		0.69	[0.10; 4.65]	.		.	.
Azithromycin(0.25g):Penicillin(3megaunits)	0	0	0.94	[0.06; 14.43]	.		0.94	[0.06; 14.43]	.		.	.
Azithromycin(0.5g):Ceftriaxone(1g)	0	0	0.74	[0.02; 27.01]	.		0.74	[0.02; 27.01]	.		.	.
Azithromycin(0.5g):Ceftriaxone(2g)	0	0	1.34	[0.16; 11.01]	.		1.34	[0.16; 11.01]	.		.	.
Azithromycin(0.5g):Doxycycline(0.2g)	0	0	2.38	[0.43; 13.16]	.		2.38	[0.43; 13.16]	.		.	.
Azithromycin(0.5g):Doxycycline(0.2g)	2	1.00	0.67	[0.18; 2.51]	0.67	[0.18; 2.51]	.		.		.	.
Azithromycin(0.5g):Penicillin(1megaunit)	0	0	10.81	[0.56; 207.76]	.		10.81	[0.56; 207.76]	.		.	.
Azithromycin(0.5g):Penicillin(3.9g)	0	0	2.34	[0.42; 13.16]	.		2.34	[0.42; 13.16]	.		.	.
Azithromycin(0.5g):Penicillin(3megaunits)	1	1.00	3.20	[0.66; 15.45]	3.20	[0.66; 15.45]	.		.		.	.
Ceftriaxone(1g):Ceftriaxone(2g)	0	0	1.82	[0.03; 118.22]	.		1.82	[0.03; 118.22]	.		.	.
Ceftriaxone(1g):Doxycycline(0.2g)	0	0	3.23	[0.06; 174.57]	.		3.23	[0.06; 174.57]	.		.	.
Ceftriaxone(1g):Doxycycline(0.2g)	0	0	0.92	[0.02; 42.45]	.		0.92	[0.02; 42.45]	.		.	.
Ceftriaxone(1g):Penicillin(1megaunit)	0	0	14.70	[0.14; 1553.59]	.		14.70	[0.14; 1553.59]	.		.	.
Ceftriaxone(1g):Penicillin(3.9g)	0	0	3.19	[0.06; 173.17]	.		3.19	[0.06; 173.17]	.		.	.
Ceftriaxone(1g):Penicillin(3megaunits)	1	1.00	4.35	[0.17; 111.20]	4.35	[0.17; 111.20]	.		.		.	.
Ceftriaxone(2g):Doxycycline(0.2g)	1	1.00	1.78	[0.52; 6.10]	1.78	[0.52; 6.10]	.		.		.	.
Ceftriaxone(2g):Doxycycline(0.2g)	0	0	0.50	[0.04; 6.05]	.		0.50	[0.04; 6.05]	.		.	.
Ceftriaxone(2g):Penicillin(1megaunit)	0	0	8.09	[0.54; 121.17]	.		8.09	[0.54; 121.17]	.		.	.
Ceftriaxone(2g):Penicillin(3.9g)	0	0	1.75	[0.30; 10.22]	.		1.75	[0.30; 10.22]	.		.	.
Ceftriaxone(2g):Penicillin(3megaunits)	0	0	2.39	[0.17; 33.25]	.		2.39	[0.17; 33.25]	.		.	.
Doxycycline(0.2g):Doxycycline(0.2g)	0	0	0.28	[0.03; 2.45]	.		0.28	[0.03; 2.45]	.		.	.
Doxycycline(0.2g):Penicillin(1megaunit)	1	0.95	4.55	[0.41; 50.63]	5.56	[0.47; 65.48]	0.07	[0.00; 5480.65]	81.91	[0.00; 8638047.97]	0.75	0.4553
Doxycycline(0.2g):Penicillin(3.9g)	1	1.00	0.99	[0.28; 3.48]	0.99	[0.28; 3.48]	.		.		.	.
Doxycycline(0.2g):Penicillin(3megaunits)	0	0	1.35	[0.13; 13.76]	.		1.35	[0.13; 13.76]	.		.	.
Doxycycline(0.2g) :Penicillin(1megaunit)	0	0	16.04	[0.63; 407.05]	.		16.04	[0.63; 407.05]	.		.	.
Doxycycline(0.2g) :Penicillin(3.9g)	0	0	3.48	[0.40; 30.40]	.		3.48	[0.40; 30.40]	.		.	.
Doxycycline(0.2g) :Penicillin(3megaunits)	0	0	4.75	[0.61; 36.87]	.		4.75	[0.61; 36.87]	.		.	.
Penicillin(1megaunit):Penicillin(3.9g)	0	0	0.22	[0.01; 3.29]	.		0.22	[0.01; 3.29]	.		.	.
Penicillin(1megaunit):Penicillin(3megaunits)	0	0	0.30	[0.01; 8.42]	.		0.30	[0.01; 8.42]	.		.	.
Penicillin(3.9g):Penicillin(3megaunits)	0	0	1.36	[0.13; 14.11]	.		1.36	[0.13; 14.11]	.		.	.

Legend:
comparison - Treatment comparison
k - Number of studies providing direct evidence
prop - Direct evidence proportion
nma - Estimated treatment effect (OR) in network meta-analysis
direct - Estimated treatment effect (OR) derived from direct evidence
indir. - Estimated treatment effect (OR) derived from indirect evidence
RoR - Ratio of Ratios (direct versus indirect)
z - z-value of test for disagreement (direct versus indirect)
p-value - p-value of test for disagreement (direct versus indirect)
>

FIG S18 Pairwise comparison and inconsistency test of safety for different daily dosages of antibiotics treating EM

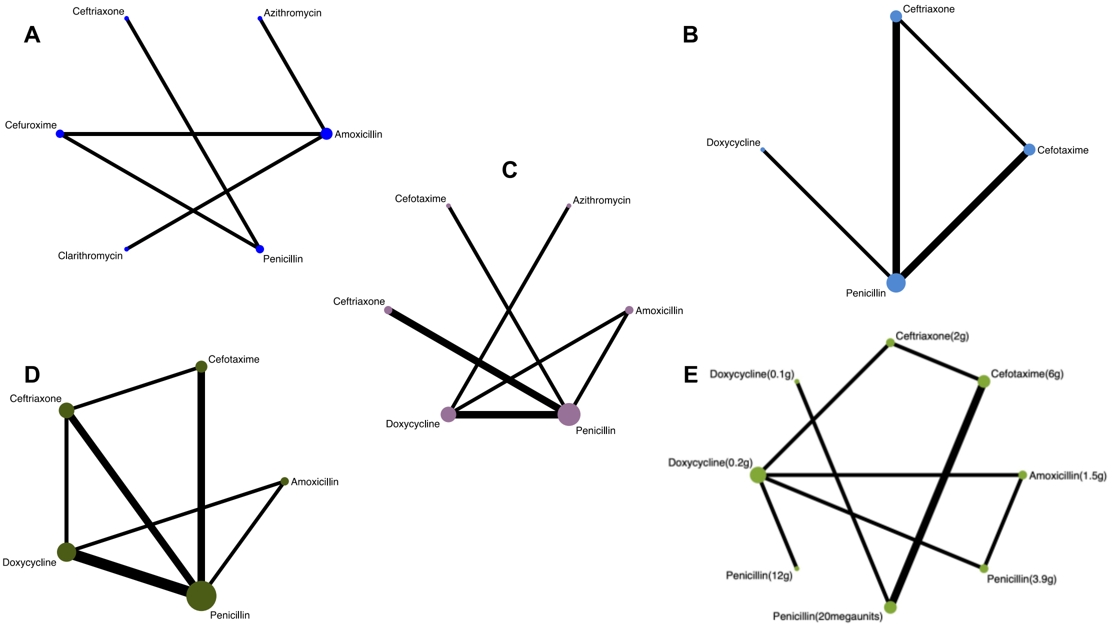

FIG S19 Network Meta-analysis Graphs of Treatments.

Line width is proportional to the number of studies comparing every pair of treatment. Size of every circle is proportional to the number of patients. A, network meta-analysis (NMA) graph of comparison of drugs' efficacy for treating children's LD. B, NMA graph of comparison of injectable drugs' efficacy for treating LD. C, NMA graph of comparison of drugs' efficacy for treating LA. D, NMA graph of comparison of drugs' efficacy for treating LNB. E, NMA graph of comparison of efficacy of different daily dosages for treating LNB.

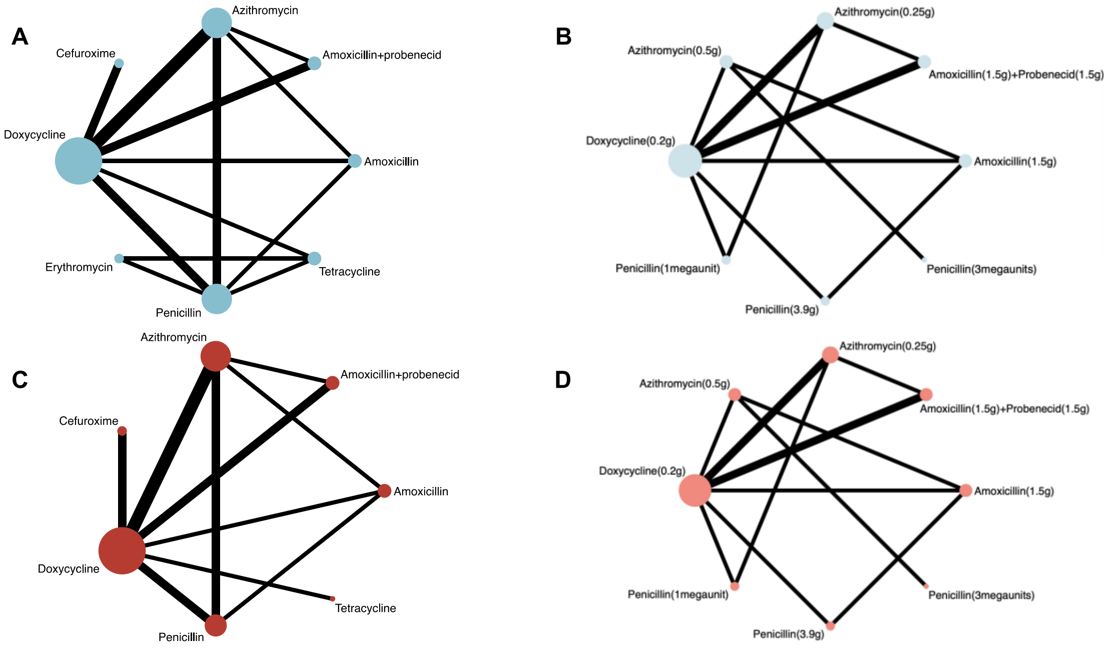

FIG S20 Network Meta-analysis Graphs of Oral Drugs.

Line width is proportional to the number of studies comparing every pair of treatment. Size of every circle is proportional to the number of patients. A, network meta-analysis (NMA) graph of comparison of oral drugs' efficacy for treating LD. B, NMA graph of comparison of efficacy of different daily dosages for treating LD. C, NMA graph of comparison of drugs' safety for treating LD. D, NMA graph of comparison of safety of different daily dosages for treating LD.

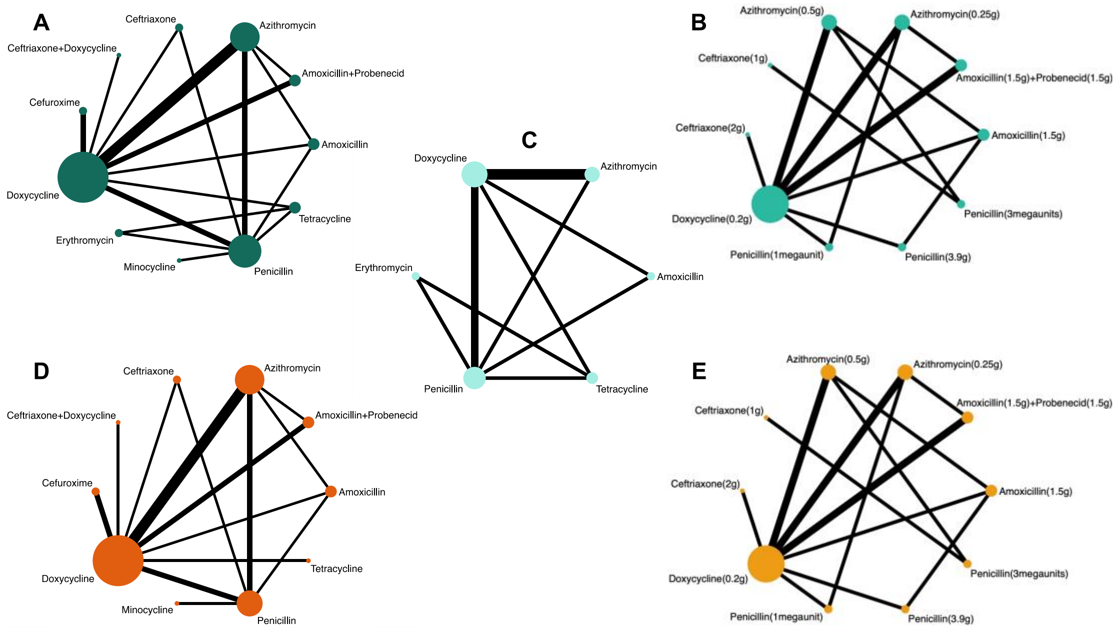

FIG S21 Network Meta-analysis Graphs of Drugs Treating EM.

Line width is proportional to the number of studies comparing every pair of treatment. Size of every circle is proportional to the number of patients. A, network meta-analysis (NMA) graph of comparison of drugs' efficacy for treating EM. B, NMA graph of comparison of efficacy of different daily dosages for treating EM. C, NMA graph of comparison of drugs' therapy duration for treating EM. D, NMA graph of comparison of drugs' safety for treating EM. E, NMA graph of comparison of safety of different daily dosages for treating EM.

Reference S

1. Arnez M, Radsel-Medvescek A, Pleterski-Rigler D, Ruzic-Sabljic E, Strle F. 1999. Comparison of cefuroxime axetil and phenoxymethyl penicillin for the treatment of children with solitary erythema migrans. Wiener Klinische Wochenschrift 111:916-922.
2. Luft BJ, Volkman DJ, Halperin JJ, Dattwyler RJ. 1988. New chemotherapeutic approaches in the treatment of Lyme borreliosis. Annals of the New York Academy of Sciences 539:352-361.
3. Ljøstad U, Skogvoll E, Eikeland R, Midgard R, Skarpaas T, Berg Å, Mygland Å. 2008. Oral doxycycline versus intravenous ceftriaxone for European Lyme neuroborreliosis: a multicentre, non-inferiority, double-blind, randomised trial. The Lancet Neurology 7:690-695.
4. Müllegger R, Millner M, Spork K, Stanek G. 1991. Penicillin G sodium and ceftriaxone in the treatment of neuroborreliosis in children—a prospective study. Infection 19:279-283.
5. Pfister H-W, Preac-Mursic V, Wilske B, Schielke E, Sörgel F, Einhaupl KM. 1991. Randomized comparison of ceftriaxone and cefotaxime in Lyme neuroborreliosis. Journal of Infectious Diseases 163:311-318.
6. Nizič T, Velikanje E, Ružić-Sabljić E, Arnež M. 2012. Solitary erythema migrans in children: comparison of treatment with clarithromycin and amoxicillin. Wiener klinische Wochenschrift 124:427-433.
7. Massarotti EM, Luger SW, Rahn DW, Messner RP, Wong JB, Johnson RC, Steere AC. 1992. Treatment of early Lyme disease. The American journal of medicine 92:396-403.
8. Dattwyler R, Volkman D, Halperin J, Luft B. 1988. Treatment of late Lyme borreliosis—randomised comparison of ceftriaxone and penicillin. The Lancet 331:1191-1194.
9. Kohlhepp W, Oschmann P, Mertens H-G. 1989. Treatment of Lyme borreliosis. Journal of neurology 236:464-469.
10. Steere AC, Hutchinson GJ, Rahn DW, SIGAL LH, CRAFT JE, DeSANNA ET, MALAWISTA SE. 1983. Treatment of the early manifestations of Lyme disease. Annals of Internal Medicine 99:22-26.
11. Weber K, Preac-Mursic V, Wilske B, Thurmayr R, Neubert U, Scherwitz C. 1990. A randomized trial of ceftriaxone versus oral penicillin for the treatment of early European Lyme borreliosis. Infection 18:91-96.
12. Dattwyler R, Volkman D, Conaty S, Platkin S, Luft B. 1990. Amoxycillin plus probenecid versus doxycycline for treatment of erythema migrans borreliosis. The Lancet 336:1404-1406.
13. Luft BJ, Dattwyler RJ, Johnson RC, Luger SW, Bosler EM, Rahn DW, Masters EJ, Grunwaldt E, Gadgil SD. 1996. Azithromycin compared with amoxicillin in the treatment of erythema migrans: a double-blind, randomized, controlled trial. Annals of internal medicine 124:785-791.
14. Arnež M, Ružic-Sabljic E. 2015. Azithromycin is equally effective as amoxicillin in children with solitary erythema migrans. The Pediatric infectious disease journal 34:1045-1048.
15. Strle F, Cimperman J, Maraspin V, Jereb M, Preac-Mursic V, Ružič E. 1993. Azithromycin versus doxycycline for treatment of erythema migrans: clinical and microbiological findings. Infection 21:83-88.
16. Hassler D, Zöller L, Haude M, Sonntag H, Hufnage H, Heinrich F. 1990. Cefotaxime versus penicillin in the late stage of Lyme disease—prospective, randomized therapeutic study. Infection 18:16-20.
17. Pfister H-W, Preac-Mursic V, Wilske B, Einhäupl KM. 1989. Cefotaxime vs penicillin G for acute neurologic manifestations in Lyme borreliosis: a prospective randomized study. Archives of neurology 46:1190-1194.

18. Dattwyler RJ, Luft BJ, Kunkel MJ, Finkel MF, Wormser GP, Rush TJ, Grunwaldt E, Agger WA, Franklin M, Oswald D. 1997. Ceftriaxone compared with doxycycline for the treatment of acute disseminated Lyme disease. New England Journal of Medicine 337:289-295.
19. Eppes SC, Childs JA. 2002. Comparative study of cefuroxime axetil versus amoxicillin in children with early Lyme disease. Pediatrics 109:1173-1177.
20. Barsic B, Maretic T, Majerus L, Strugar J. 2000. Comparison of azithromycin and doxycycline in the treatment of erythema migrans. Infection 28:153-156.
21. Nadelman RB, Luger SW, Frank E, Wisniewski M, Collins JJ, Wormser GP. 1992. Comparison of cefuroxime axetil and doxycycline in the treatment of early Lyme disease. Annals of internal medicine 117:273-280.
22. Luger SW, Paparone P, Wormser GP, Nadelman RB, Grunwaldt E, Gomez G, Wisniewski M, Collins JJ. 1995. Comparison of cefuroxime axetil and doxycycline in treatment of patients with early Lyme disease associated with erythema migrans. Antimicrobial agents and chemotherapy 39:661-667.
23. Karlsson M, Hammers-Berggren S, Lindquist L, Stiernstedt G, Svenungsson B. 1994. Comparison of intravenous penicillin G and oral doxycycline for treatment of Lyme neuroborreliosis. Neurology 44:1203-1203.
24. Oksi J, Nikoskelainen J, Viljanen MK. 1998. Comparison of oral cefixime and intravenous ceftriaxone followed by oral amoxicillin in disseminated Lyme borreliosis. European Journal of Clinical Microbiology and Infectious Diseases 17:715-719.
25. Eliassen K, Reiso H, Berild D, Lindbæk M. 2018. Comparison of phenoxymethylpenicillin, amoxicillin, and doxycycline for erythema migrans in general practice. A randomized controlled trial with a 1-year follow-up. Clinical Microbiology and Infection 24:1290-1296.
26. Nowakowski J, Nadelman RB, Forseter G, McKenna D, Wormser GP. 1995. Doxycycline versus tetracycline therapy for Lyme disease associated with erythema migrans. Journal of the American Academy of Dermatology 32:223-227.
27. Wormser GP, Ramanathan R, Nowakowski J, McKenna D, Holmgren D, Visintainer P, Dornbush R, Singh B, Nadelman RB. 2003. Duration of antibiotic therapy for early Lyme disease: a randomized, double-blind, placebo-controlled trial. Annals of internal medicine 138:697-704.
28. Oksi J, Nikoskelainen J, Hiekkanen H, Lauhio A, Peltomaa M, Pitkäranta A, Nyman D, Granlund H, Carlsson S-A, Seppälä I. 2007. Duration of antibiotic treatment in disseminated Lyme borreliosis: a double-blind, randomized, placebo-controlled, multicenter clinical study. European Journal of Clinical Microbiology & Infectious Diseases 26:571-581.
29. Strle F, Ružič E, Cimperman J. 1992. Erythema migrans: comparison of treatment with azithromycin, doxycycline and phenoxymethylpenicillin. Journal of Antimicrobial Chemotherapy 30:543-550.
30. Breier F, Kunz G, Klade H, Aberer E, Stanek G. 1996. Erythema migrans: three weeks treatment for prevention of late Lyme borreliosis. Infection 24:69-72.
31. Weber K, Wilske B, Preac-Mursic V, Thurmayr R. 1993. Azithromycin versus penicillin V for the treatment of early Lyme borreliosis. Infection 21:367-372.
